# Role of hydroxyurea therapy in the prevention of organ damage in sickle cell disease: a systematic review and meta-analysis

**DOI:** 10.1186/s13643-024-02461-z

**Published:** 2024-02-08

**Authors:** Naveen Khargekar, Anindita Banerjee, Shreyasi Athalye, Namrata Mahajan, Neha Kargutkar, Prashant Tapase, Manisha Madkaikar

**Affiliations:** 1 Department of Haematogenetics, ICMR-National Institute of Immunohaematology, 13th Floor, New MS Building, KEM Hospital Campus, Mumbai, Parel 400 012 India; 2 Department of Transfusion Transmitted Disease, ICMR-National Institute of Immunohaematology, 13th Floor, New MS Building, KEM Hospital Campus, Mumbai, Parel 400 012 India; 3 Department of Paediatric Immunology & Leukocyte Biology, ICMR-National Institute of Immunohaematology, 13th Floor, New MS Building, KEM Hospital Campus, Parel Mumbai, 400 012 India

**Keywords:** Sickle cell disease, Organ damage, Hydroxyurea, Meta-analysis

## Abstract

**Background:**

Hydroxyurea is an affordable drug that reduces vaso-occlusive crises and transfusion requirements in sickle cell disease. However, its effectiveness in preventing chronic organ damage is still unclear. This systematic review and meta-analysis aimed to evaluate the role of hydroxyurea in preventing organ morbidity.

**Method:**

We included original articles published in English from 1st January 1990 to 31st January 2023, reporting hydroxyurea therapy and organ damage from PubMed, Google Scholar, Scopus, and CrossRef databases. A total of 45 studies with 4681 sickle cell disease patients were evaluated for organ damage.

**Results:**

Our analysis showed that hydroxyurea intervention significantly lowered transcranial Doppler and tricuspid regurgitant velocity, with a standardized mean difference of − 1.03 (− 1.49; − 0.58); *I* ^2^ = 96% and − 1.37 (CI − 2.31, − 0.42); *I* ^2^ = 94%, respectively. Moreover, the pooled estimate for albuminuria showed a beneficial effect post-hydroxyurea therapy by reducing the risk of albuminuria by 58% (risk ratio of 0.42 (0.28; 0.63); *I* ^2^ = 28%).

**Conclusion:**

Our study found that a hydroxyurea dose above 20 mg/kg/day with a mean rise in HbF by 18.46% post-hydroxyurea therapy had a beneficial role in reducing transcranial doppler velocity, tricuspid regurgitant velocity, albuminuria, and splenic abnormality.

**Systematic review registration:**

PROSPERO CRD42023401187.

**Supplementary Information:**

The online version contains supplementary material available at 10.1186/s13643-024-02461-z.

## Introduction

 Sickle cell disease (SCD) is a monogenetic disorder caused by a point mutation in the 6th position of the β-chain of globin, leading to abnormal hemoglobin production [1]. SCD is a major public health problem affecting more than 3,000,000 births globally. Predominantly found in Sub-Saharan Africa and India, the number of SCD cases is expected to increase by 30% in these regions by 2050 due to the high birth rate [2–4].

Studies have shown that Asian sickle cell patients have a relatively milder clinical presentation, compared to Africans. The presence of alpha-thalassemia, persistent high fetal hemoglobin (HbF) levels, genetic factors (BLC11A, HBS1L-MYB, and HBB loci), hematological parameters, social circumstances, climatic and geographical variation affect the clinical severity in SCD [5–10].

Under hypoxic conditions, Haemoglobin S (HBS) polymerizes and undergoes a rapid change in the shape of erythrocytes, leading to membrane destabilization, chronic hemolysis, systemic inflammation, and endothelial dysfunction. This leads to activation of adhesion molecules like P selectin, and platelet accumulation which give rise to small vessel obstruction and organ damage [11]. This is exacerbated by the ischemia/reperfusion process (I/R), macrovascular hyperemia, and microvascular hypoperfusion referred to as perfusion paradox. This condition is extremely challenging for vital organs like the brain, kidney, and heart, which may fail to respond and adapt to the need for increased oxygen [12]. Considering the unique combination and capability of different organs in handling hypoxia, innate immune response, coagulability, inflammatory and oxidative stress, and genetic, ethnic, and environmental age-dependent drivers, the spectrum of organ damage in SCD is diverse [13]. Chronic hemolytic anemia and recurrent episodes of ischemia–reperfusion injury contribute to progressive organ dysfunction.

Currently, hydroxyurea is the only ideal and affordable drug with global availability and good clinical efficacy for treating SCD patients. Hydroxyurea is a potent HbF inducer and myelosuppressor by nature [14, 15]. The National Heart, Lung, and Blood Institute (NHLBI) has recommended it to all sickle cell anemia children above 9 months of age irrespective of the clinical severity [16]. Hydroxyurea has excellent oral bioavailability and is rapidly cleared from circulation with a half-life of 2–3 h in both children and adults [17]. It is a well-tolerated drug with a few short and long-term toxicities with the most common toxicity being reversible cytopenia [15].

In a multicentric randomized controlled trial with 20 mg/kg/day hydroxyurea versus placebo (BABYHUG Trial) among SCD children aged 9–18 months, the authors found that children on hydroxyurea had lower rates of acute crises and hospitalization [18]. Dose escalation to a maximum tolerated dose of hydroxyurea has been shown to elicit significantly better hematological and clinical response compared to a standard dose of 20 mg/kg/day [19].

Despite enough evidence of hydroxyurea preventing acute symptoms in SCD, there is a lack of clarity on whether and to what extent hydroxyurea prevents organ damage in SCD patients. Therefore, this systematic review and meta-analysis are planned to investigate whether hydroxyurea therapy in SCD patients reduces organ damage and to evaluate the influence of HbF level and hydroxyurea dose in the prevention of organ damage.

## Methods

### Search strategy and selection criteria

For this systematic review and meta-analysis, we searched PubMed, Google Scholar, CrossRef, and Scopus for articles evaluating organ damage in SCD patients treated with Hydroxyurea. We used the following search terms: “Sickle cell Disease” AND “Hydroxyurea” AND “Organ Damage” (see Appendix 1 for the full search strategy). We included all the published articles from 1st January 1990 to 31st January 2023. Original articles in the English language with Abstract and/or Full text of articles for sickle cell disease patients screened for organ damage were considered. The studies included case reports, retrospective studies, letters to editors, cross-sectional studies, cohort studies, and randomized controlled trials. Articles either in a foreign language or not containing relevant information or review articles were excluded. Articles that evaluated the effect of hydroxyurea in SCD patients without the mention of organ damage were also excluded from the study.

### Data analysis

Two reviewers AB and NK independently scrutinized and extracted the articles using Rayyan Software [20]. Conflicts and disagreements were resolved by discussion with the third reviewer, MM. The quality of the included studies was assessed using a modified Downs & Black checklist which scores each item as one point (yes) or zero (no), excluding the power question [21]. The total score determined the overall quality of the study, which was used to classify as good (25 and above), average (15–24), and poor (less than 15). Post-hoc power calculations were performed using G*power software and power scored using a 6-point scale [22].

The data was extracted by NK and AB using a standardized data format from studies that measured organ dysfunction/damage in SCD patients on hydroxyurea. Information was recorded in a customized electronic spreadsheet with details of authors, year of publication, study design, the study population country, type of publication, sample size, age, hydroxyurea dose, duration of hydroxyurea, HBF% at baseline, and follow-up and organ damage.

Data analysis was performed using the ‘meta’ and ‘metafor’ packages in R Studio, Build 576 with R for Windows. The software packages contain functions to estimate effect size with common effect and random effects, generate forest plots, and funnel plots, as well as sub-group and meta-regression analysis. The difference between the parameters for organ morbidity in SCD patients on hydroxyurea therapy and not on hydroxyurea therapy was calculated using a mean difference with a 95% confidence interval. The *I* ^2^ statistic was used to report the heterogeneity in the study, whereas Funnel plots were used to report publication bias. The Preferred Reporting Items for Systematic Reviews and Meta-Analysis (PRISMA) 2020 guidelines were followed throughout this systematic review and meta-analysis [23]. The review protocol was registered in the International Prospective Register of Systematic Reviews, PROSPERO.(https://www.crd.york.ac.uk/prospero/display_record.php?RecordID=401187, registration number: CRD42023401187, accessed on 14 March 2023).

## Results

**Fig. 1:**
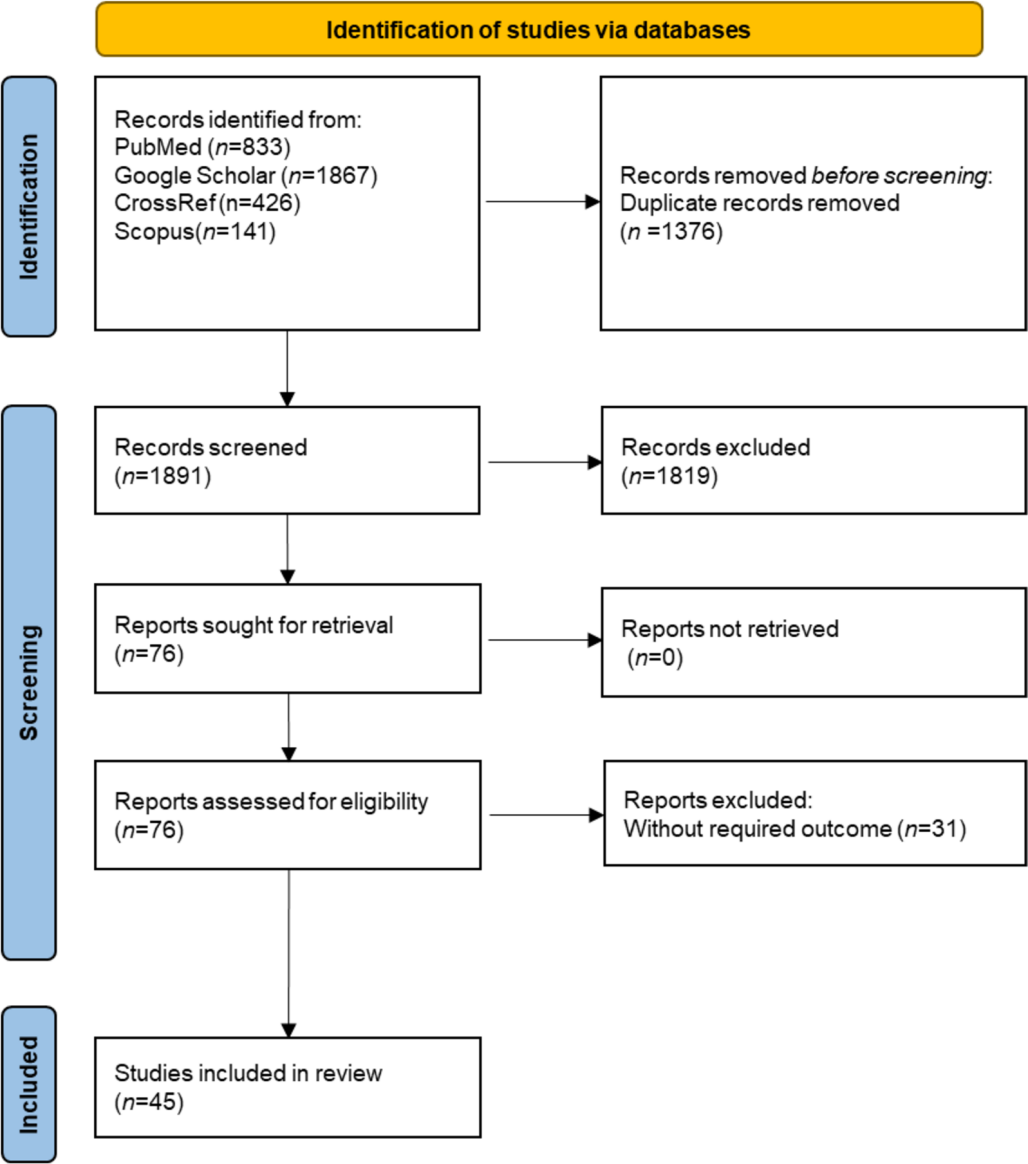
PRISMA 2020 flowchart diagram for the study selection process

We identified a total of 3267 articles with our search strategy. Of these, 1376 were removed as duplicates, and 1815 were excluded after screening titles and abstracts. Next, 76 articles were retrieved out of which 31 articles were excluded according to our exclusion criteria as mentioned. Finally, a total of 45 articles were included in the systematic review. The agreement between the reviewers was 82.5% (Cohen’s kappa 0.97) and 100% before and after conflict resolution. The flowchart of the screening procedure is depicted in Fig. 1 and study characteristics are detailed in Appendix 2.

1. Central nervous system

- Transcranial Doppler

**Fig. 2:**
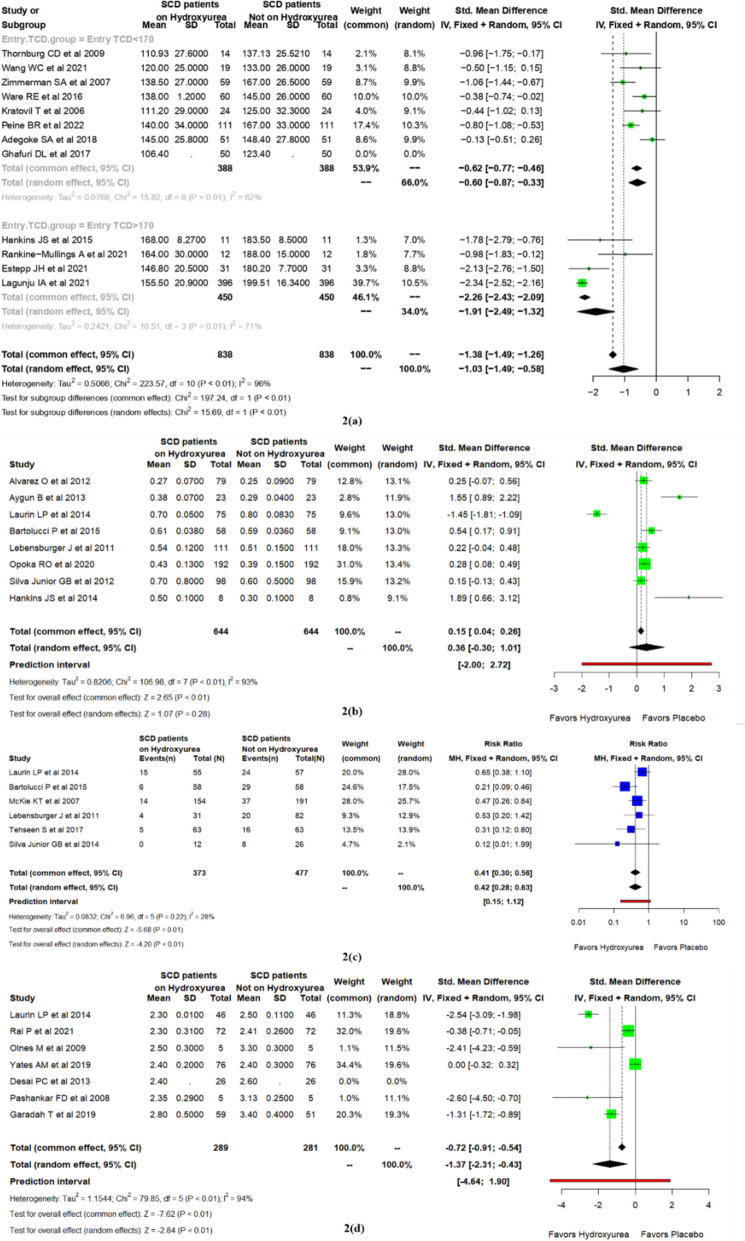
Forest plot of **a** difference in transcranial Doppler flow velocities, **b** difference in creatinine levels, **c** risk ratio of microalbuminuria, **d** difference in tricuspid regurgitant velocities in sickle cell disease patients with or without hydroxyurea treatment

Figure 2a shows the measurement of velocities of the cerebral artery by Transcranial Doppler (TCD)at baseline and post-hydroxyurea intervention of the included studies. The pooled estimates show a significant reduction in TCD velocity post-hydroxyurea treatment, with a standardized mean difference of – 1.03 (CI – 1.48, − 0.57; *p* < 0·0001). The mean dose of hydroxyurea was 24·54 mg/kg/day, and the mean HbF level was 21.55% post-hydroxyurea therapy [24–35]. On meta-regression, we found that the covariates, including HbF % at baseline, dose, and duration of hydroxyurea therapy, and percentage increase in HbF post-hydroxyurea therapy significantly influenced the reduction of TCD Velocity, (see Appendix 4). Further subgroup analysis with baseline HbF% levels and duration of hydroxyurea therapy showed that baseline HbF% > 10 and hydroxyurea therapy irrespective of duration significantly reduced the TCD velocity (see Appendix 6). Among the studies with baseline TCD values above 170 cm/s, there was a significant reduction in TCD values after hydroxyurea therapy with a standardized mean difference of − 1.91(CI − 2.49; − 1.32; *p* = 0.01). In a study by Abdullahi SU et al. among 220 SCD children with a median age of 7.2 years having abnormal TCD (TAMMC ≥ 200 cm/s), the TCD value dropped to normal levels (< 170 cm/s) after a median duration of hydroxyurea therapy of 2.4 years. The TCD drop to normal levels was 48.9% (92) in the low-dose hydroxyurea arm(10 mg/kg/day) and 71.4% (95) in the moderate-dose hydroxyurea arm (20 mg/kg/day) [36].

b) Stroke

Table 1 shows the incidence rate of stroke in SCD patients. The incidence rate of stroke in SCD patients was slightly higher in those who were on hydroxyurea, but this difference was not statistically significant. The studies’ mean dose of hydroxyurea and HbF levels post-hydroxyurea therapy ranged from 20 to 30 mg per kg per day and 14% to 23.1% respectively [27, 37–39].

**Table 1:** Incidence of stroke in SCD patients on hydroxyurea therapy and not on hydroxyurea therapy

Sr.no	Authors with year of publication	Mean age (months)	Not on hydroxyurea therapy			On hydroxyurea therapy			Hydroxyurea dose (mg/kg/day)
			*N*	Incidence of stroke /100-person year^a^	Mean HbF level	N	Incidence of stroke /100-person year	Mean HbF level	
1	Hankins JS et al. 2008	132	25	2.54	4	18	1.9	18.2	30
2	Wang WC et al. 2001	15	16	10	21.8	14	7.14	20.2	20
3	Nottage KA et al. 2016	113	50	4.04	5	30	1.1	14	20
4	Wang WC et al. 2021	149	19	3.82	8.2	9	11.10	23.1	23.8

Mean incidence rate of stroke in SCD not on hydroxyurea: 5.1, SD 3·3

Mean incidence rate of stoke on hydroxyurea: 5.31, SD 4.6

Unpaired *t*-test: *t* = 0.0742 df = 6 standard error of difference = 5.161

The two-tailed *P* value equals 0.9433

^a^Mean age of patients is considered as the total duration of non-exposure to hydroxyurea and the person-years is calculated by mean age in years X total number of patients

c) Cerebral oxygenation

Hydroxyurea also improves cerebral oxygen saturation in SCD patients. In a study by Tavakkoli F et al. involving 31 SCD patients, which measured near-infrared spectroscopy (NIRS) to determine cerebral oxygen saturation, it was observed that cerebral oxygen saturation was significantly higher in SCD patients on hydroxyurea therapy than in those not on hydroxyurea therapy (46.1 ± 6.6 vs 41.2 ± 7.6,* p* < 0.025) [40]. Another study by Karkoska K et al. showed that cerebral oxygen saturation significantly increased after 2 years of hydroxyurea therapy in 55 SCD patients (65 to 72%, *p* < 0.001) [41]. A study by Kapustin D et al. among 27 SCD patients, showed that the mean white matter cerebrovascular reactivity was higher in hydroxyurea-treated patients compared to those who were not treated. (0.10 ± 0.03 vs 0.07 ± 0.03, *p* = 0.08) [42].

A study by Puffer E et al. among 65 SCD children, showed that SCD children on hydroxyurea therapy performed better on verbal comprehension, fluid reasoning, and general cognitive ability compared to SCD children not on medication [43]. A case report by Grace R F et al. showed that cerebral artery stenosis was normalized in a 4-year-old SCD child after 4 years of hydroxyurea therapy (17 mg/kg/day) [44].

2) Renal

- Glomerular filtration rate

Table 2 shows the glomerular filtration rate (GFR) in SCD patients [24, 45–50]. The study by Alvarez O et al. reported an increase in GFR even after treatment with 20 mg/kg/day hydroxyurea, but this rise was significantly lower compared to age-matched SCD patients who were treated with a placebo [45]. In a study by Aygun B et al. in SCD patients with a mean age of 74 years, there was a mild reduction in GFR post-hydroxyurea therapy (*t* = 1.97, df 44 standard error of difference = 11.122, *p* = 0.054) [46]. The other 4 studies by Hankins JS et al., Silva Junior GB et al., Bartolucci P et al., and Laurin LP et al. were conducted in adult SCD patients in whom the GFR normally declines, making it difficult to conclude that hydroxyurea intervention reduced the GFR [47–50].

**Table 2:** GFR in SCD patients on hydroxyurea therapy and not on hydroxyurea therapy

Sr.no	Authors	*N*	Mean age (years)	GFR entry	On hydroxyurea (months)	GFR exit	Hydroxyurea dose (mg/kg/day)
				Mean (SD)		Mean (SD)	
1	Alvarez O et al. 2012	193	1.15	126.42 (38.8)^b^	24	146.64 (43·7)	20
2	Thornburg CD et al. 2009	14	2·.91	139.2 (19.5)^b^	24	144.3 (15.69)	28
3	Aygun B et al. 2013	23	7.4	167 (46)^a^	25	145 (27)	24.4
4	Hankins JS et al. 2014	8	17.1	140.7 (17.5)	72	117.7 (22.5)	26.6
5	Silva Junior GB et al. 2014	26	32.1	105 (30)^a^	NA	112 (21)	1000 g^c^
6	Bartolucci P et al. 2015	58	35	124.5 (33.3)^a^	6	120.5 (2.83)	15 mg
7	Laurin LP et al. 2014	70	37	151 (55)^a^	73	128 (58)	1207 mg^c^

^a^Estimated GFR

^b^DTPA

^c^Dose of hydroxyurea per day

b) Serum creatinine

Figure 2b shows the measurement of serum creatinine in SCD patients [45–53]. The pooled estimates showed a slight increase in serum creatinine level after hydroxyurea intervention 0.36 (CI – 0.3, 1.01; *p* = 0.28), but the difference was not statistically significant. The mean dose of hydroxyurea therapy was 21.20 mg/kg/day and the mean HbF level post-hydroxyurea therapy was 22.17%.

c) Urinary albumin

Figure 2c shows the measurement of urinary albumin levels in SCD patients [48–51, 54, 55]. The pooled estimates show a significant reduction in urinary excretion of albumin after hydroxyurea treatment with a risk ratio of 0.42 (0.28; 0.63); *I* ^2^ = 28%; *p* < 0·01. The mean dose of hydroxyurea therapy in these studies was 19.17 mg/kg/day and the mean exit HbF level was 13.55%. On meta-regression, the covariates; hydroxyurea dose, and percentage increase in HbF after hydroxyurea therapy significantly influenced the reduction of albuminuria, whereas baseline HbF% and duration of hydroxyurea therapy did not affect the outcomes, (see Appendix 4). Further sub-group analysis was performed with baseline HbF% (< 10 or > 10) and age of SCD patients (< 18 years and > 18 years) in which we observed that baseline HbF levels did not influence the reduction of albuminuria. In contrast, adult patients had lower albuminuria compared to SCD children, (see Appendix 6).

d) Serum cystatin

In a study conducted by Alvarez o et al. among 193 SCD patients with a mean age of 13.8 months on 20 mg/kg/day hydroxyurea, the serum cystatin level at baseline and 24 months after hydroxyurea therapy were 0.91 ± 0.17 mg/l and 0.92 ± 0.13 mg/l respectively [44]. A similar finding was found in the study by Ayugun B in 23 SCD patients with a mean age of 7.4 years on a mean dose of 24.4 mg/kg/day hydroxyurea for 25 months, the serum cystatin level at baseline and post hydroxyurea therapy was 0.72 ± 0.09 mg/l and 0.74 ± 0.13 mg/l [46]. There was no significant difference in serum cystatin level after initiation of hydroxyurea therapy in SCD patients.

3) Spleen

SCD patients have splenic dysfunction which increases with age. Table 3 shows the splenic abnormality in SCD patients on hydroxyurea therapy and not on hydroxyurea therapy. Studies conducted by Hankins JS et al. and Santos A et al. showed that there was more than 90% of SCD patients aged above 10 years had markedly decreased or absent splenic function [37, 56]. In a study by Hankins JS et al. involving 43 SCD patients, 14% of SCD children had complete recovery of splenic function after the maximum tolerated dose(MTD) of hydroxyurea for a median period of 2.6 years [37]. Another study by Nottage KA et al. involving 40 SCD patients(mean age of 9.1 years) treated with a median dose of 27 mg/kg/day hydroxyurea for 3 years, showed that 33% of SCD patients had improved splenic uptake [57]. A similar finding was observed in a study by Santos A. et al. where 6(42.85%) had mild to moderate improvement after hydroxyurea therapy with a maximum tolerated dose for 1 year [56]. Case reports of two SCD patients published by Susan Claster et al. in 1996, demonstrated that there was splenic regeneration in two adult SCD patients after hydroxyurea therapy [58].

**Table 3:** Splenic abnormality in SCD patients on hydroxyurea therapy and not on hydroxyurea therapy

Sr.no	Authors with year of publication	Total number of SCD patients	Splenic uptake abnormality at baseline^ab^	Splenic uptake abnormality after hydroxyurea treatment	Hydroxyurea dose	Duration of hydroxyurea therapy (years)
1	Hankins JS et al. 2008 [36]	43	95%	81.40%	MTD up to 30–35 mg/kg/day	2.6
2	Hankins JS et al. 2005 [59]	14	NA	78.50%	30 mg ± 1.2 mg/kg/day	4
3	Nottage KA et al. 2014 [57]	40	77·50%	67.50%	20 mg escalated to MTD	3
4	Wang WC et al. 2001 [37]	17	100%	94.11%	20 mg/kg/day	2
5	Santos A et al. 2002 [56]	21	100%	92.85	15 mg/kg/day with dose escalation	1
6	Wang WC et al. 2011 [60]	144	38%	27%	20 mg/kg/day	2

Any gain in spleen function after hydroxyurea treatment is considered as normal splenic function

^a^At baseline or in the non-hydroxyurea group/placebo group

^b^Decline in splenic uptake from normal to decreased or absent, or from decreased to absent)

In a study among 14 splenectomized SCD children (median age of 3.4 years) treated with hydroxyurea with a mean dose of 30 ± 1.2 mg/kg/day for 4 years, 43% of children had functional asplenia. There is a loss of splenic function in SCD patients even though they are on hydroxyurea therapy, but the loss was much lower compared to age-matched functional asplenia [58, 59]. In the BABY HUG Trial, for the pediatric SCD patients who were on 20 mg/kg/day, 19(27.14%) patients had decreased spleen function after 2 years compared to 28(37.8%) in the placebo group (*p* = 0·21) [60].

4) Cardiovascular

- Tricuspid regurgitation velocity

Figure 2d shows the measurement of Tricuspid regurgitant velocities in SCD patients [50, 61–66]. The pooled estimates show a significant reduction in TRV post-hydroxyurea treatment with a standardized mean difference of – 1.37(CI − 2·31, − 0.42; *p* = 0.004). The mean dose of hydroxyurea therapy in these studies was 22.66 mg/kg/day and the mean HbF level was 18.08%. Meta-regression of TRV showed that the covariates; HbF at baseline, and percentage increase in HbF after hydroxyurea therapy, significantly influenced the reduction of TRV velocity (see Appendix 4).

5) Avascular necrosis of hip joint

In a prospective study involving 40 SCD patients having a mean age of 12.9 ± 4.2 years at enrolment, 11(27.5%) had avascular necrosis (AVN) hip joints of varying severity. Post hydroxyurea therapy of 20 mg/kg/day, 2(6.9%) developed new AVN. Five (50%) of SCD patients who were on hydroxyurea for more than 5 years had the worst AVN hip joint [66, 67]. In another prospective study by Kris M. Mahadeo et al. among 257 SCD patients screened for osteonecrosis of the femoral head, the prevalence of avascular necrosis of the hip joint who were on hydroxyurea therapy was 18(21.68%) which was higher compared to the prevalence of AVN who were not on hydroxyurea therapy 8(8.08%) [68].

6) Retina

A study by Estepp JH et al., among 123 SCD children aged ≤ 19 years, revealed that 10.6% developed retinopathy. In SCD children who never developed retinopathy, hydroxyurea was initiated at a median age of 8.8 years with a median MTD of 26 mg/kg/day whereas, in SCD children who developed retinopathy, hydroxyurea was started at 10.6 years with a median MTD of 27 mg/kg/day. Children treated with hydroxyurea who never developed retinopathy had higher HbF levels (20.8%) at the last clinical follow-up compared to HbF levels (12.5%) at the time of diagnosis in children who developed retinopathy [69].

7) Respiratory system

SCD patients develop progressive changes in pulmonary function testing with decreased lung volumes and flows. The airflow limitation and airway hyperresponsiveness are associated with increased morbidity and premature death. In a study conducted among 56 SCD patients, hydroxyurea therapy for a mean period of 4.7 years showed significantly improved rates of decline in FEV1 and FEF25-75% and FVC [70]. In another study by Kotwal N et al., 62 SCD children (mean age 9.8 ± 3.8 years) were treated with hydroxyurea, and 30 SCD children (mean age 10.7 ± 4.9 years) were not on hydroxyurea. The authors observed a significant increase in forced vital capacity in the hydroxyurea group after 3 years of follow-up while children in the non-hydroxyurea group showed a decline in forced vital capacity after 2.6 years of follow-up (7.2 ± 17.1 vs 3.4 ± 18.2, *p* < 0.01) [71]. Hydroxyurea therapy in children with SCA leads to improvement in annual pulmonary function decline.

Meta-regression on the factors affecting the effect of hydroxyurea in preventing organ damage. The meta-regression results predict the protective role of hydroxyurea therapy on TCD velocity, albuminuria, and TRV of SCD patients (Appendix 4). In the case of TCD velocity, HbF baseline (*p* = 0.007), therapy duration (*p* < 0.001), percent increase in HbF (*p* < 0.001) as well as HU dose (*p* = 0.018) significantly affected the TCD velocities. However, in the case of albuminuria, only the HU dose (*p* < 0.001) and percent increase in HbF (*p* < 0.001) affected the albuminuria levels. Similarly, only the HbF levels at baseline (*p* < 0.001) and the percent increase in HbF (*p* < 0.002) affected the TRV levels in SCD patients.

## Discussion

This is the first systematic review to investigate the effects of hydroxyurea treatment on multi-organ dysfunction in individuals with SCD. Our review included 45 studies with a total sample size of 4681. Randomized controlled trials, cross-sectional studies, cohort studies, case–control studies, and case series were included in our meta-analysis. Of the 45 studies included, 11 were classified as poor (score less than 15), 28 were classified as average (score between 15 and 24) and 6 were classified as good (score of 25 and above). Effects of hydroxyurea were assessed before and after treatment in terms of different indicators of organ function.

For assessing brain infarction/stroke risk, TCD velocity in the cerebral artery above 200 cm/s is indicated as an increased risk of stroke in SCD patients. The pooled estimates of 12 studies that reported the TCD velocities before and after hydroxyurea therapy showed a significant decrease in TCD velocity in SCD patients. The results were influenced by the dose and duration of hydroxyurea therapy, baseline HbF%, and percentage rise in HbF levels. The mean dose of hydroxyurea ranged from 20 mg/kg/day to 27.9 mg/kg/day (mean 23.14 mg/kg/day) and the post-hydroxyurea therapy HbF levels ranged from 11.79% to 25.9% (mean 18.46%). Further subgroup analysis suggested that SCD patients who had higher baseline HbF levels had a more significant reduction in TCD velocity. Four studies measured the incidence of stroke in SCD patients before and after hydroxyurea therapy and the overall incidence of stroke was slightly higher in SCD patients on hydroxyurea therapy compared with those not on hydroxyurea therapies. The limitations to comparing the incidence of stroke in two groups were (a) mean age was considered as the total duration of non-exposure of hydroxyurea for calculating the incidence of stroke in person-years, (b) age is an important risk factor for stroke, as age increases, there is always a greater risk of stroke and there can be an increased incidence of stroke after treatment of the hydroxyurea group, (c) there is wide variability in clinical severity in SCD patients and there are SCD patients who are susceptible to stroke.

GFR, serum creatinine, and microalbuminuria are the parameters used to assess renal dysfunction. GFR in SCD increases from infancy till early adulthood and thereby declines to normal levels [68]. Studies in SCD patients below the mean age of 3 years showed a slight increase in GFR, but it was significantly lower compared to those who were not treated with hydroxyurea. This suggests that hydroxyurea intervention in younger patients potentially prevents a rise in GFR. However, the measurement of GFR as a marker for renal dysfunction in older SCD patients is not accurate, as the GFR normally declines after the second decade of life [72]. The meta-analysis of six studies reporting microalbuminuria levels before and after hydroxyurea therapy showed that hydroxyurea therapy significantly reduced the microalbuminuria (risk ratio 0.42 (0.28; 0.63); *I* ^2^ = 28%; *p* < 0.01). Furthermore, subgroup analysis showed that hydroxyurea therapy irrespective of baseline HbF level has a protective role against renal dysfunction. This protective role was more significant in adults compared to children. Pooled estimates from eight studies measuring serum creatinine before and after hydroxyurea therapy found a slight increase in mean creatinine levels after therapy with a standardized mean difference of 0.36(CI − 0.3, 1.01; *p* = 0.28). This can be explained by the fact that creatinine is a relatively late marker of renal damage and hydroxyurea therapy may or may not be beneficial once there is significant kidney dysfunction.

Sickle cell disease (SCD) can cause various cardiovascular complications such as pulmonary hypertension, left ventricular diastolic heart disease, myocardial infarction, and dysrhythmia. The pooled estimates of the 6 studies showed that the TRV was significantly reduced post-hydroxyurea therapy with a mean dose of 22.66 mg/kg/day and the mean HbF level post-hydroxyurea therapy was 18.08% suggesting that hydroxyurea therapy was beneficial in preventing cardiac dysfunction in SCD patients. TRV reduction was influenced by baseline and percentage rise in HbF. Concerning respiratory complications, there were only two studies that reported a beneficial effect of hydroxyurea in preventing the decline of pulmonary function test parameters.

Studies conducted by Hankins JS et al. 2008 Hankins JS et al. 2005 Nottage KA et al. 2014, Wang WC et al. 2001, Santos A et al. 2002, and Wang WC et al. 2011 showed a reduction in splenic uptake abnormality post-hydroxyurea therapy. In the BABY HUG Trial, the splenic abnormality was lower in the hydroxyurea group compared to the placebo group, but this difference was not statistically significant. Case reports also have shown splenic regeneration after hydroxyurea therapy. Overall, from the studies, we can conclude that hydroxyurea therapy helps in preserving splenic function to some extent, even though the results might not be statistically significant in RCT when compared to placebo.

There is limited evidence to suggest whether hydroxyurea therapy has any role in preventing liver dysfunction and retinopathy in SCD patients. In addition, there is limited evidence regarding the avascular necrosis of the hip joints in patients who are on hydroxyurea therapy. It has been postulated that hydroxyurea therapy increases fetal hemoglobin and hematocrit leading to increased blood viscosity and sickling in the microcirculation of the femoral head [68].

It is now evident that hydroxyurea in a dose of above 20 mg/kg/day (mean 23.14 mg/kg/day) with a rise in HbF% post hydroxyurea therapy of (mean 18.46%) prevents major organ dysfunction in the brain, kidney, heart, and spleen. All these RCTs are done on SCD patients in Africa or on those of African origin. Arab Indian haplotypes have higher baseline HbF levels and overall clinical severity in these patients is less even though some of them despite high HbF may have a severe phenotype. Suboptimal dose of hydroxyurea is a serious concern in the treatment of SCD patients and there is a need to evaluate the efficacy of suboptimal dose of hydroxyurea therapy in the prevention of organ complications, especially in patients with high baseline HbF.

Meta-regression analysis of multiple factors influencing the effectiveness of hydroxyurea therapy in preventing organ damage showed that few factors such as baseline levels of HbF, duration of hydroxyurea therapy, dose of hydroxyurea as well as percent increase in HbF levels post therapy significantly affected the outcome. However, these effects could only be studied in the TRV, TCD, and albuminuria outcomes. The other outcomes could not be analyzed due to a limited number of studies.

Our study has several limitations. Firstly, the included studies were diverse and differed in mean age, dose, and duration of hydroxyurea therapy. Secondly, the parameters studied for various organ dysfunction were not uniform and the duration and dose of hydroxyurea varied in each study. Thirdly, most of the studies were observational with very few randomized controlled trials conducted only on the African SCD population. Very few studies were available that evaluated avascular necrosis of the hip joint in SCD patients.

## Conclusion

Hydroxyurea has been proven through randomized controlled trials to be an effective drug in reducing acute sickle-related events in patients with sickle cell disease. Our meta-analysis has shown that hydroxyurea can reduce TCD velocity, TRV, and urinary albuminuria, potentially reducing organ damage. However, the role of hydroxyurea in preventing stroke is inconclusive and needs more evidence. In addition, the beneficial effect of hydroxyurea in dysfunction of the liver, retina, pulmonary system, and avascular necrosis of the hip joint needs to be further evaluated. Therefore we conclude that hydroxyurea therapy may be effective in preventing organ damage in Sickle cell disease patients.

### Supplementary Information

**Additional file 1: Appendix 1. **Search terms and search strategy. **Appendix 2.** Study characteristics of the included studies. **Appendix 3.** Mean hydroxyurea dose and exit HbF levels. **Appendix 4.** Meta-regression. **Appendix 5.** Funnel plots and sensitivity analysis. **Appendix 6.** Sub-group analysis. **Appendix 7.** Full-length studies excluded with reasons for exclusion. **Appendix 8.** Data quality of included studies. **Appendix 9.** PRISMA 2020 checklist.

## Data Availability

All data supporting the findings of this study are available within the paper and its Supplementary Information.

## References

[1] Inusa B, Hsu L, Kohli N (2019). Sickle cell disease—genetics, pathophysiology, clinical presentation and treatment. IJNS.

[2] Wastnedge E, Waters D, Patel S (2018). The global burden of sickle cell disease in children under five years of age: a systematic review and meta-analysis. J Glob Health.

[3] Sedrak A, Kondamudi NP. Sickle cell disease. In: StatPearls. Treasure Island (FL): StatPearls Publishing, 2023. http://www.ncbi.nlm.nih.gov/books/NBK482384/. Accessed 14 Mar 2023. 29494006

[4] Opoka RO, Ndugwa CM, Latham TS (2017). Novel use of hydroxyurea in an African Region with malaria (NOHARM): a trial for children with sickle cell anemia. Blood.

[5] Serjeant GR (1993). The clinical features of sickle cell disease. Baillière’s Clinical Haematology.

[6] Kulozik AE, Wainscoat JS, Serjeant GR (1986). Geographical survey of βS-globin gene haplotypes: evidence for an independent Asian origin of the sickle-cell mutation. Am J Hydroxyuream Genet.

[7] Mukherjee MB, Nadkarni AH, Gorakshakar AC, Ghosh K, Mohanty D, Colah RB (2010). Clinical, hematologic and molecular variability of sickle cell-β thalassemia in western India. Indian J Hydroxyuream Genet.

[8] Mukherjee MB, Lu CY, Ducrocq R (1997). Effect of alpha-thalassemia on sickle-cell anemia linked to the Arab-Indian haplotype in India. Am J Hematol.

[9] Pandey S, Pandey S, Mishra RM, Sharma M, Saxena R (2011). Genotypic influence of α-deletions on the phenotype of Indian sickle cell anemia patients. Korean J Hematol.

[10] Pandey SK, Pandey S, Ranjan R (2014). Phenotypic effect of α-globin gene numbers on Indian sickle β-thalassemia patients. J Clin Lab Anal.

[11] Belcher JD, Chen C, Nguyen J (2014). Heme triggers TLR4 signaling leading to endothelial cell activation and vaso-occlusion in murine sickle cell disease. Blood.

[12] Hebbel RP, Belcher JD, Vercellotti GM (2020). The multifaceted role of ischemia/reperfusion in sickle cell anemia. J Clin Invest.

[13] Allali S, Taylor M, Brice J, de Montalembert M (2021). Chronic organ injuries in children with sickle cell disease. Haematologica.

[14] Agrawal RK, Patel RK, Shah V, Nainiwal L, Trivedi B (2014). Hydroxyurea in sickle cell disease: drug review. Indian J Hematol Blood Transfus.

[15] McGann PT, Ware RE (2015). Hydroxyurea therapy for sickle cell anemia. Expert Opin Drug Saf.

[16] Yawn BP, Buchanan GR, Afenyi-Annan AN (2014). Management of sickle cell disease: summary of the 2014 evidence-based report by expert panel members. JAMA.

[17] Ware RE, Despotovic JM, Mortier NA (2011). Pharmacokinetics, pharmacodynamics, and pharmacogenetics of hydroxyurea treatment for children with sickle cell anemia. Blood.

[18] Thornburg CD, Files BA, Luo Z (2012). Impact of hydroxyurea on clinical events in the BABY HYDROXYUREAG trial. Blood.

[19] John CC, Opoka RO, Latham TS (2020). Hydroxyurea dose escalation for sickle cell anemia in Sub-Saharan Africa. N Engl J Med.

[20] Ouzzani M, Hammady H, Fedorowicz Z, Elmagarmid A (2016). Rayyan—a web and mobile app for systematic reviews. Syst Rev.

[21] Downs SH, Black N (1998). The feasibility of creating a checklist for the assessment of the methodological quality both of randomised and non-randomised studies of health care interventions. J Epidemiol Community Health.

[22] Faul F, Erdfelder E, Buchner A, Lang A-G (2009). Statistical power analyses using G*Power 3.1: Tests for correlation and regression analyses. Behavior Research Methods.

[23] Page MJ, McKenzie JE, Bossuyt PM (2021). The PRISMA 2020 statement: an updated guideline for reporting systematic reviews. BMJ.

[24] Thornburg CD, Dixon N, Burgett S (2009). A pilot study of hydroxyurea to prevent chronic organ damage in young children with sickle cell anemia. Pediatr Blood Cancer.

[25] Hankins JS, McCarville MB, Rankine-Mullings A (2015). Prevention of conversion to abnormal transcranial Doppler with hydroxyurea in sickle cell anemia: a phase III international randomized clinical trial. Am J Hematol.

[26] Rankine-Mullings A, Reid M, Soares D (2021). Hydroxycarbamide treatment reduces transcranial Doppler velocity in the absence of transfusion support in children with sickle cell anaemia, elevated transcranial Doppler velocity, and cerebral vasculopathy: the EXTEND trial. Br J Haematol.

[27] Wang WC, Zou P, Hwang SN (2021). Effects of hydroxyurea on brain function in children with sickle cell anemia. Pediatr Blood Cancer.

[28] Zimmerman SA, Schydroxyurealtz WH, Burgett S, Mortier NA, Ware RE (2007). Hydroxyurea therapy lowers transcranial Doppler flow velocities in children with sickle cell anemia. Blood.

[29] Ware RE, Davis BR, Schydroxyurealtz WH (2016). Hydroxycarbamide versus chronic transfusion for maintenance of transcranial doppler flow velocities in children with sickle cell anaemia-TCD With Transfusions Changing to Hydroxyurea (TWiTCH): a multicentre, open-label, phase 3, non-inferiority trial. Lancet.

[30] Kratovil T, Bulas D, Driscoll MC, Speller-Brown B, McCarter R, Minniti CP (2006). Hydroxyurea therapy lowers TCD velocities in children with sickle cell disease. Pediatr Blood Cancer.

[31] Peine BR, Callaghan MU, Callaghan JH, Glaros AK (2022). Prophylactic hydroxyurea treatment is associated with improved cerebral hemodynamics as a surrogate marker of stroke risk in sickle cell disease: a retrospective comparative analysis. J Clin Med.

[32] Adegoke SA, Macedo-Campos R de S, Braga JAP, Figueiredo MS (2018). Changes in transcranial doppler flow velocities in children with sickle cell disease: the impact of hydroxyurea therapy. J Stroke Cerebrovasc Dis.

[33] Estepp JH, Cong Z, Agodoa I (2021). What drives transcranial Doppler velocity improvement in paediatric sickle cell anaemia: analysis from the Sickle Cell Clinical Research and Intervention Program (SCCRIP) longitudinal cohort study. Br J Haematol.

[34] Ghafuri DL, Chaturvedi S, Rodeghier M, *et al.* Secondary benefit of maintaining normal transcranial Doppler velocities when using hydroxyurea for prevention of severe sickle cell anemia. *Pediatr**Blood Cancer* 2017; 64.:10.1002/pbc.26401. 10.1002/pbc.26401 28035747

[35] Lagunju IA, Labaeka A, Ibeh JN, Orimadegun AE, Brown BJ, Sodeinde OO (2021). Transcranial Doppler screening in Nigerian children with sickle cell disease: A 10-year longitudinal study on the SPPIBA cohort. Pediatr Blood Cancer.

[36] Hankins JS, Helton KJ, McCarville MB, Li C-S, Wang WC, Ware RE (2008). Preservation of spleen and brain function in children with sickle cell anemia treated with hydroxyurea. Pediatr Blood Cancer.

[37] Wang WC, Wynn LW, Rogers ZR, Scott JP, Lane PA, Ware RE (2001). A two-year pilot trial of hydroxyurea in very young children with sickle-cell anemia. J Pediatr.

[38] Abdullahi SU, Wudil BJ, Bello-Manga H (2021). Primary prevention of stroke in children with sickle cell anemia in sub-Saharan Africa: rationale and design of phase III randomized clinical trial. PediatrHematol Oncol.

[39] Nottage KA, Ware RE, Aygun B (2016). Hydroxycarbamide treatment and brain MRI/MRA findings in children with sickle cell anaemia. Br J Haematol.

[40] Tavakkoli F, Nahavandi M, Wyche MQ, Castro O (2005). Effects of hydroxyurea treatment on cerebral oxygenation in adult patients with sickle cell disease: an open-label pilot study. Clin Ther.

[41] Karkoska K, Quinn CT, Niss O, Pfeiffer A, Dong M, Vinks AA, McGann PT (2021). Hydroyxurea improves cerebral oxygen saturation in children with sickle cell anemia. Am J Hematol.

[42] Kapustin D, Leung J, Odame I, Williams S, Shroff M, Kassner A (2019). Hydroxycarbamide treatment in children with Sickle Cell Anaemia is associated with more intact white matter integrity: a quantitative MRI study. Br J Haematol.

[43] Puffer E, Schatz J, Roberts CW (2007). The association of oral hydroxyurea therapy with improved cognitive functioning in sickle cell disease. Child Neuropsychol.

[44] Grace RF, Su H, Sena L, Poussaint TY, Heeney MM, Gutierrez A (2010). Resolution of cerebral artery stenosis in a child with sickle cell anemia treated with hydroxyurea. Am J Hematol.

[45] Alvarez O, Miller ST, Wang WC (2012). Effect of hydroxyurea treatment on renal function parameters: results from the multi-center placebo-controlled BABY HYDROXYUREAG clinical trial for infants with sickle cell anemia. Pediatr Blood Cancer.

[46] Aygun B, Mortier NA, Smeltzer MP, Shydroxyurealkin BL, Hankins JS, Ware RE (2013). Hydroxyurea treatment decreases glomerular hyperfiltration in children with sickle cell anemia. Am J Hematol.

[47] Hankins JS, Aygun B, Nottage K (2014). From infancy to adolescence: fifteen years of continuous treatment with hydroxyurea in sickle cell anemia. Medicine (Baltimore).

[48] Silva Junior GB, Vieira APF, Couto Bem AX (2014). Proteinuria in adults with sickle-cell disease: the role of hydroxycarbamide(hydroxyurea) as a protective agent. Int J Clin Pharm.

[49] Bartolucci P, Habibi A, Stehlé T (2016). Six months of hydroxyurea reduces albuminuria in patients with sickle cell disease. J Am Soc Nephrol.

[50] Laurin L-P, Nachman PH, Desai PC, Ataga KI, Derebail VK (2014). Hydroxyurea is associated with lower prevalence of albuminuria in adults with sickle cell disease. Nephrol Dial Transplant.

[51] Lebensburger J, Johnson SM, Askenazi DJ, Rozario NL, Howard TH, Hilliard LM (2011). Protective role of hemoglobin and fetal hemoglobin in early kidney disease for children with sickle cell anemia. Am J Hematol.

[52] Opoka RO, Hydroxyureame HA, Latham TS (2020). Hydroxyurea to lower transcranial Doppler velocities and prevent primary stroke: the Uganda NOHARM sickle cell anemia cohort. Haematologica.

[53] Silva Junior GB, Libório AB, Vieira APF (2012). Evaluation of renal function in sickle cell disease patients in Brazil. Braz J Med Biol Res.

[54] McKie KT, Hanevold CD, Hernandez C, Waller JL, Ortiz L, McKie KM (2007). Prevalence, prevention, and treatment of microalbuminuria and proteinuria in children with sickle cell disease. J PediatrHematol Oncol.

[55] Tehseen S, Joiner CH, Lane PA, Yee ME. Changes in urine albumin to creatinine ratio with the initiation of hydroxyurea therapy among children and adolescents with sickle cell disease. Pediatr Blood Cancer 2017; 64. 10.1002/pbc.26665. 10.1002/pbc.26665 28612449

[56] Santos A, Pinheiro V, Anjos C (2002). Scintigraphic follow-up of the effects of therapy with hydroxyurea on splenic function in patients with sickle cell disease. Eur J Nucl Med Mol Imaging.

[57] Nottage KA, Ware RE, Winter B (2014). Predictors of splenic function preservation in children with sickle cell anemia treated with hydroxyurea. Eur J Haematol.

[58] Claster S, Vichinsky E (1996). First report of reversal of organ dysfunction in sickle cell anemia by the use of hydroxyurea: splenic regeneration. Blood.

[59] Hankins JS, Ware RE, Rogers ZR (2005). Long-term hydroxyurea therapy for infants with sickle cell anemia: the HYDROXYUREASOFT extension study. Blood.

[60] Wang WC, Ware RE, Miller ST (2011). Hydroxycarbamide in very young children with sickle-cell anaemia: a multicentre, randomised, controlled trial (BABY HYDROXYUREAG). Lancet.

[61] Rai P, Joshi VM, Goldberg JF (2021). Longitudinal effect of disease-modifying therapy on tricuspid regurgitant velocity in children with sickle cell anemia. Blood Adv.

[62] Olnes M, Chi A, Haney C (2009). Improvement in hemolysis and pulmonary arterial systolic pressure in adult patients with sickle cell disease during treatment with hydroxyurea. Am J Hematol.

[63] Yates AM, Joshi VM, Aygun B (2019). Elevated tricuspid regurgitation velocity in congenital hemolytic anemias: prevalence and laboratory correlates. Pediatr Blood Cancer.

[64] Desai PC, May RC, Jones SK (2013). Longitudinal study of echocardiography-derived tricuspid regurgitant jet velocity in sickle cell disease. Br J Haematol.

[65] Pashankar FD, Carbonella J, Bazzy-Asaad A, Friedman A (2009). Longitudinal follow up of elevated pulmonary artery pressures in children with sickle cell disease. Br J Haematol.

[66] Garadah T, Mandeel F, Jaradat A, Bin TK (2019). The effects of hydroxyurea therapy on the six-minute walk distance in patients with adult sickle cell anemia: an echocardiographic study. J Blood Med.

[67] Adekile AD, Gupta R, Al-Khayat A, Mohammed A, Atyani S, Thomas D (2019). Risk of avascular necrosis of the femoral head in children with sickle cell disease on hydroxyurea: MRI evaluation. Pediatr Blood Cancer.

[68] Mahadeo KM, Oyeku S, Taragin B (2011). Increased prevalence of osteonecrosis of the femoral head in children and adolescents with sickle-cell disease. Am J Hematol.

[69] Estepp JH, Smeltzer MP, Wang WC, Hoehn ME, Hankins JS, Aygun B (2013). Protection from sickle cell retinopathy is associated with elevated HbF levels and hydroxycarbamide use in children. Br J Haematol.

[70] McLaren A, Klingel M, Behera S, Odame I, Kirby-Allen M, Grasemann H (2017). Effect of hydroxyurea therapy on pulmonary function in children with sickle cell anemia. Am J Respir Crit Care Med.

[71] Kotwal N, Pillai DK, Darbari DS, Sun K, Koumbourlis AC (2022). Spirometric changes after initiation of hydroxyurea in children with sickle cell anemia. J Pediatr Hematol Oncol.

[72] Afangbedji N, Jerebtsova M (2022). Glomerular filtration rate abnormalities in sickle cell disease. Front Med (Lausanne).

