# Supplementary material for: Role of hydroxyurea therapy in the prevention of organ damage in sickle cell disease: a systematic review and meta-analysis
Source: Syst Rev. 2024 Feb 8;13:60. doi: 10.1186/s13643-024-02461-z (PMC10851507; doi:10.1186/s13643-024-02461-z)
Supplement: Supplementary file 1 — Additional file 1: Appendix 1. Search terms and search strategy. Appendix 2. Study characteristics of the included studies. Appendix 3. Mean hydroxyurea dose and exit HbF levels. Appendix 4. Meta-regression. Appendix 5. Funnel plots and sensitivity analysis. Appendix 6. Sub-group analysis. Appendix 7. Full-length studies excluded with reasons for exclusion. Appendix 8. Data quality of included studies. Appendix 9. PRISMA 2020 checklist. [file 13643_2024_2461_MOESM1_ESM.docx]

**Supplementary Information:**

| Sr. No. | Contents | Page Nos. |
| --- | --- | --- |
| 1) | Appendix I: Search Terms and Search Strategy | 2–3 |
| 2) | Appendix II: Study Characteristics of the Included Studies | 4–5 |
| 3) | Appnedix III: Mean Hydroxyurea Dose and Exit HbF levels | 6 |
| 4) | Appendix IV: Meta Regression | 6–8 |
| 5) | Appendix V: Funnel Plots and Sensitivity analysis | 9–12 |
| 6) | Appendix VI: Sub Group Analysis | 13–21 |
| 7) | Appendix VII: Full length studies excluded with reasons for exclusion | 22–23 |
| 8) | Appendix VIII: Data Quality of included studies | 24–33 |
| 9) | Appendix IX: PRISMA 2020 checklist | 34–37 |
|  | References | 37–41 |

**Appendix I: Search Terms and Search Strategy**

**PubMed:**

1. SCD/Sickle Cell Anemia/Sickle Cell Disease
2. Hydroxyurea/Hydroxycarbamide
3. Organ Damage/Organ Dysfunction
4. Renal Dysfunction/Kidney Dysfunction/Microalbuminuria/Glomerular Filtration Rate/GFR/Creatinine.
5. Stroke/Infarct/Cerebrovascular Events/TCD/Transcranial Doppler
6. Hepatic Dysfunction//Liver Dysfunction/Liver enzymes
7. Splenic Dysfunction/ Auto Splenectomy/Splenic Uptake/Splenic Regeneration.
8. Cardiac Dysfunction/Heart/TRV/Tricuspid regurgitant velocity
9. Retina Abnormalities/Eye Abnormalities
10. Avascular Necrosis of Hip joint/AVN/Skeletal Abnormalities.
11. 1 and 2
12. 3 or 4 or 5 or 6or 7 or 8 or 9 or 10
13. 11 and 12

**Google Scholar:**

1. SCD/Sickle Cell Anemia/Sickle Cell Disease
2. Hydroxyurea/Hydroxycarbamide
3. Organ Damage/Organ Dysfunction
4. Renal Dysfunction/Kidney Dysfunction/Microalbuminuria/Glomerular Filtration Rate/GFR/Creatinine.
5. Stroke/Infarct/Cerebrovascular Events/TCD/Transcranial Doppler
6. Hepatic Dysfunction//Liver Dysfunction/Liver enzymes
7. Splenic Dysfunction/ Auto Splenectomy/Splenic Uptake/Splenic Regeneration.
8. Cardiac Dysfunction/Heart/TRV/Tricuspid regurgitant velocity
9. Retina Abnormalities/Eye Abnormalities
10. Avascular Necrosis of Hip joint/AVN/Skeletal Abnormalities.
11. 1 and 2
12. 3 or 4 or 5 or 6or 7 or 8 or 9 or 10
13. 11 and 12

**Cross Ref:**

1. SCD/Sickle Cell Anemia/Sickle Cell Disease
2. Hydroxyurea/Hydroxycarbamide
3. Organ Damage/Organ Dysfunction
4. Renal Dysfunction/Kidney Dysfunction/Microalbuminuria/Glomerular Filtration Rate/GFR/Creatinine.
5. Stroke/Infarct/Cerebrovascular Events/TCD/Transcranial Doppler
6. Hepatic Dysfunction//Liver Dysfunction/Liver enzymes
7. Splenic Dysfunction/ Auto Splenectomy/Splenic Uptake/Splenic Regeneration.
8. Cardiac Dysfunction/Heart/TRV/Tricuspid regurgitant velocity
9. Retina Abnormalities/Eye Abnormalities
10. Avascular Necrosis of Hip joint/AVN/Skeletal Abnormalities.
11. 1 and 2
12. 3 or 4 or 5 or 6or 7 or 8 or 9 or 10
13. 11 and 12

**Scopus:**

1. SCD/Sickle Cell Anemia/Sickle Cell Disease
2. Hydroxyurea/Hydroxycarbamide
3. Organ Damage/Organ Dysfunction
4. Renal Dysfunction/Kidney Dysfunction/Microalbuminuria/Glomerular Filtration Rate/GFR/Creatinine.
5. Stroke/Infarct/Cerebrovascular Events/TCD/Transcranial Doppler
6. Hepatic Dysfunction//Liver Dysfunction/Liver enzymes
7. Splenic Dysfunction/ Auto Splenectomy/Splenic Uptake/Splenic Regeneration.
8. Cardiac Dysfunction/Heart/TRV/Tricuspid regurgitant velocity
9. Retina Abnormalities/Eye Abnormalities
10. Avascular Necrosis of Hip joint/AVN/Skeletal Abnormalities.
11. 1 and 2
12. 3 or 4 or 5 or 6or 7 or 8 or 9 or 10
13. 11 and 12

**Appendix II: Study Characteristics of the Included Studies.**

| **Sr. No.** | **Author and Year** | **Country** | **Study Design** | **Number of cases screened** | **Organ screened** | **Quality Score** | **Level of Evidence** |
| --- | --- | --- | --- | --- | --- | --- | --- |
| 1 | Claster S et al, 1996 | USA | Case Report | 2 | Spleen | 8 | 5 |
| 2 | Wang WC et al, 2001 | USA | Prospective Study | 28 | Spleen | 17 | 2 |
| 3 | Santos A et al, 2002 | Brazil | Prospective Study | 21 | Spleen | 12 | 2 |
| 4 | Hankins JS et al, 2005 | USA | Prospective Study | 21 | Spleen & Brain | 23 | 1 |
| 5 | Tavakkoli F et al, 2005 | USA | Prospective Study | 31 | Brain | 21 | 3 |
| 6 | Kratovil T et al, 2006 | USA | Prospective Study | 24 | Brain | 15 | 2 |
| 7 | Zimmerman SA et al, 2007 | USA | Prospective Study | 102 | Brain | 22 | 2 |
| 8 | Puffer E et al, 2007 | USA | Prospective Study | 65 | Brain | 13 | 3 |
| 9 | McKie KT et al, 2007 | Georgia | Prospective Study | 191 | Renal | 18 | 3 |
| 10 | Pashankar FD et al, 2009 | USA | Prospective Study | 62 | Heart | 18 | 2 |
| 11 | Hankins JS et al, 2008 | USA | Retrospective Study | 78 | Spleen & Brain | 16 | 3 |
| 12 | Olnes M et al, 2009 | USA | Case Series | 5 | Heart | 14 | 4 |
| 13 | Grace RF et al, 2010 | USA | Case Report | 1 | Brain | 13 | 5 |
| 14 | Thornburg CD et al, 2009 | USA | Prospective Study | 14 | Spleen | 16 | 2 |
| 15 | Mahadeo KM et al, 2011 | USA | Prospective Study | 257 | Hip Joint | 22 | 3 |
| 16 | Lebensburger J et al, 2011 | USA | Retrospective Study | 144 | Renal | 17 | 3 |
| 17 | Wang WC et al, 2011 | USA | Randomized Controlled Trial | 193 | Spleen | 24 | 1 |
| 18 | Alvarez O et al, 2012 | USA | Randomized Controlled Trial | 193 | Renal | 24 | 1 |
| 19 | Silva Junior GB et al, 2012 | Brazil | Retrospective Study | 98 | Renal | 15 | 3 |
| 20 | Desai PC et al, 2013 | USA | Prospective Study | 55 | Heart | 16 | 2 |
| 21 | Aygun B et al, 2013 | USA | Prospective Study | 23 | Renal | 11 | 2 |
| 22 | Estepp JH et al, 2013 | USA | Retrospective Study | 123 | Retina | 16 | 3 |
| 23 | Laurin L-P et al, 2014 | USA | Cross-Sectional Study | 149 | Renal & Heart | 14 | 3 |
| 24 | Silva Junior GB et al, 2014 | Brazil | Prospective Study | 26 | Renal | 15 | 3 |
| 25 | Hankins JS et al, 2014 | USA | Prospective Study | 28 | Renal | 18 | 2 |
| 26 | Nottage KA et al, 2014 | USA | Prospective Study | 40 | Spleen | 18 | 2 |
| 27 | Hankins JS et al, 2015 | USA, Brazil, Jamaica | Randomized Controlled Trial | 115 | Brain | 14 | 1 |
| 28 | Bartolucci P et al, 2016 | France | Prospective Study | 58 | Renal | 14 | 2 |
| 29 | Ware RE et al, 2016 | USA, Canada | Randomized Controlled Trial | 159 | Brain | 26 | 1 |
| 30 | Nottage KA et al, 2016 | USA | Prospective Study | 50 | Brain | 13 | 2 |
| 31 | Tehseen S et al, 2017 | USA | Retrospective Study | 63 | Renal | 17 | 3 |
| 32 | Adegoke SA et al, 2017 | Brazil | Retrospective Study | 100 | Brain | 23 | 3 |
| 33 | Ghafuri DL et al, 2017 | USA | Retrospective Study | 136 | Brain | 18 | 3 |
| 34 | Adekile AD et al, 2019 | Kuwait | Prospective Study | 40 | Hip Joint | 19 | 2 |
| 35 | Yates AM et al, 2019 | USA | Prospective Study | 343 | Heart | 26 | 3 |
| 36 | Garadah T et al, 2019 | Bahrain | Prospective Study | 110 | Heart | 20 | 3 |
| 37 | Kapustin D et al, 2019 | Canada | Prospective Study | 39 | Brain | 16 | 3 |
| 38 | Opoka RO et al, 2020 | Uganda | Prospective Study (Letter to editor) | 213 | Brain | 27 | 4 |
| 39 | Lagunju IA et al, 2021 | West Africa | Prospective Study | 396 | Brain | 26 | 2 |
| 40 | Rai P et al, 2021 | USA | Prospective Study | 204 | Heart | 26 | 2 |
| 41 | Rankine-Mullings A et al, 2021 | Jamaica | Prospective Study | 43 | Brain | 21 | 2 |
| 42 | Wang WC et al, 2021 | USA | Prospective Study | 29 | Brain | 18 | 2 |
| 43 | Estepp JH et al, 2021 | USA | Prospective Study | 196 | Brain | 18 | 2 |
| 44 | Karkoska K et al , 2021 | USA | Prospective Study | 84 | Brain | 14 | 2 |
| 45 | Peine BR et al, 2022 | USA | Retrospective Study | 329 | Brain | 25 | 3 |

**Appendix III: Mean Hydroxyurea Dose and Mean Exit HbF levels**

| **Study** | **HbF exit mean (g/dL)** | **HbF exit SD (g/dL)** | **HU Dose (mg/kg/day)** |
| --- | --- | --- | --- |
| **TRV** |  |  |  |
| Yates AM et al 2019 [63] | NA | NA | 24.7 |
| Pashankar FD et al 2009 [65] | NA | NA | 23.3 |
| Laurin LP et al 2014 [50] | 8.8 | NA | NA |
| Rai P 2021 [61] | 17.75 | 7.33 | 24.62 |
| Desai PC et al 2013 [64] | NA | NA | NA |
| Olnes M 2009 [62] | 22.9 | 9.4 | 18 |
| **Mean** | **18.08** | **7.52** | **22.66** |
| **Albumin** |  |  |  |
| Laurin LP et al 2014 [50] | 8.8 | 1.73 | NA |
| Bartolucci P et al 2015 [49] | 14.4 | 2.4 | 15 |
| McKie KT et al 2007 [54] | 12 | 7.09 | 22.5 |
| Lebensburger J et al 2011 [51] | 17.76 | 6.86 | NA |
| Silva Junior GB et al 2014 [48] | 11 | 6.8 | NA |
| Tehseen S et al 2017 [55] | 16.3 | 8.7 | 20 |
| **Mean** | **13.55** | **7.03** | **19.17** |
| **TCD** |  |  |  |
| Thornburg CD et al 2009 [24] | 25.9 | 6.6 | 20 |
| Hankins JS et al 2015 [25] | 18.8 | 5.1 | 25 |
| Rankine-Mullings A et al 2021 [26] | 18.1 | 10 | 25.4 |
| Wang WC et al 2021 [27] | 23.1 | 5.7 | 23.8 |
| Zimmerman SA et al 2007 [28] | 22.7 | 7.9 | 27.9 |
| Ware RE et al 2016 [29] | 24.4 | 7.9 | 20 |
| Kratovil T et al 2006 [30] | 11.79 | 3.6 | 23.3 |
| Estepp JH et al 2021 [33] | 20.8 | 6.9 | 27.8 |
| Peine BR et al 2022 [31] | NA | NA | 24.7 |
| Ghafuri DL et al 2017 [34] | NA | NA | 24.8 |
| Lagunju IA et al 2021 [35] | NA | NA | 27.2 |
| **Mean** | **21.55** | **8.08** | **24.54** |
| **Creatinine** |  |  |  |
| Alvarez O 2012 [45] | 22.49 | 8.66 | 20 |
| Aygun B et al 2013 [46] | 20.3 | 8.8 | 24.4 |
| Laurin LP et al 2014 [50] | 8.8 | NA | NA |
| Bartolucci P et al 2015 [49] | 14.4 | NA | 15 |
| Lebensburger J et al 2011 [51] | 17.76 | 6.86 | NA |
| Opoka RO et al 2020 [52] | 25.1 | 9.3 | 20 |
| Silva Junior GB et al 2012 [53] | NA | NA | NA |
| Hankins JS et al 2014 [47] | 15.1 | 6.9 | 26.6 |
| **Mean** | **22.17** | **9.07** | **21.20** |
| **Overall Mean (n= 20)** | **18.46** | **8.97** | **23.14** |

NA: Not Available

**Appendix IV: Meta Regression**

**Meta Regression with Baseline HbF, Hydroxyurea dose, Percentage increase in HbF from Baseline and Duration of Hydroxyurea Therapy.**

| **Covariate** | **Coefficient (CI, 95%)** | **Std. error** | **p-Value** |
| --- | --- | --- | --- |
| **Transcranial Doppler** |  |  |  |
| HbF Baseline | -0.13(-0.225,-0.035) | 0.048 | **0.007** |
| Therapy Duration | -0.025(-0.033,-0.016) | 0.005 | **< 0.001** |
| Percent Increase in HbF | 0.01(0.006,0.014) | 0.002 | **< 0.001** |
| HU dose | -0.164(-0.3,-0.028) | 0.069 | **0.018** |
| **Albuminuria** |  |  |  |
| HbF Baseline | -0·024(-0·069,0·021) | 0·023 | 0·294 |
| Therapy Duration | 0·001(-0·004,0·005) | 0·002 | 0·833 |
| Percent Increase in HbF | 0·006(0·003,0·009) | 0·001 | **< 0·001** |
| HU dose | -0·040(-0·058,-0·021) | 0·009 | **< 0·001** |
| **TRV** |  |  |  |
| HbF Baseline | -0·116(-0·153,-0·080) | 0·019 | **< 0·001** |
| Therapy Duration | -0·015(-0·039,0·008) | 0·012 | 0·202 |
| Percent Increase in HbF | 0·009(0·003,0·014) | 0·003 | **0·002** |
| HU dose | -0·283(-0·590,0·024) | 0·157 | 0·071 |

1. **For Transcranial Doppler velocity in SCD patients**


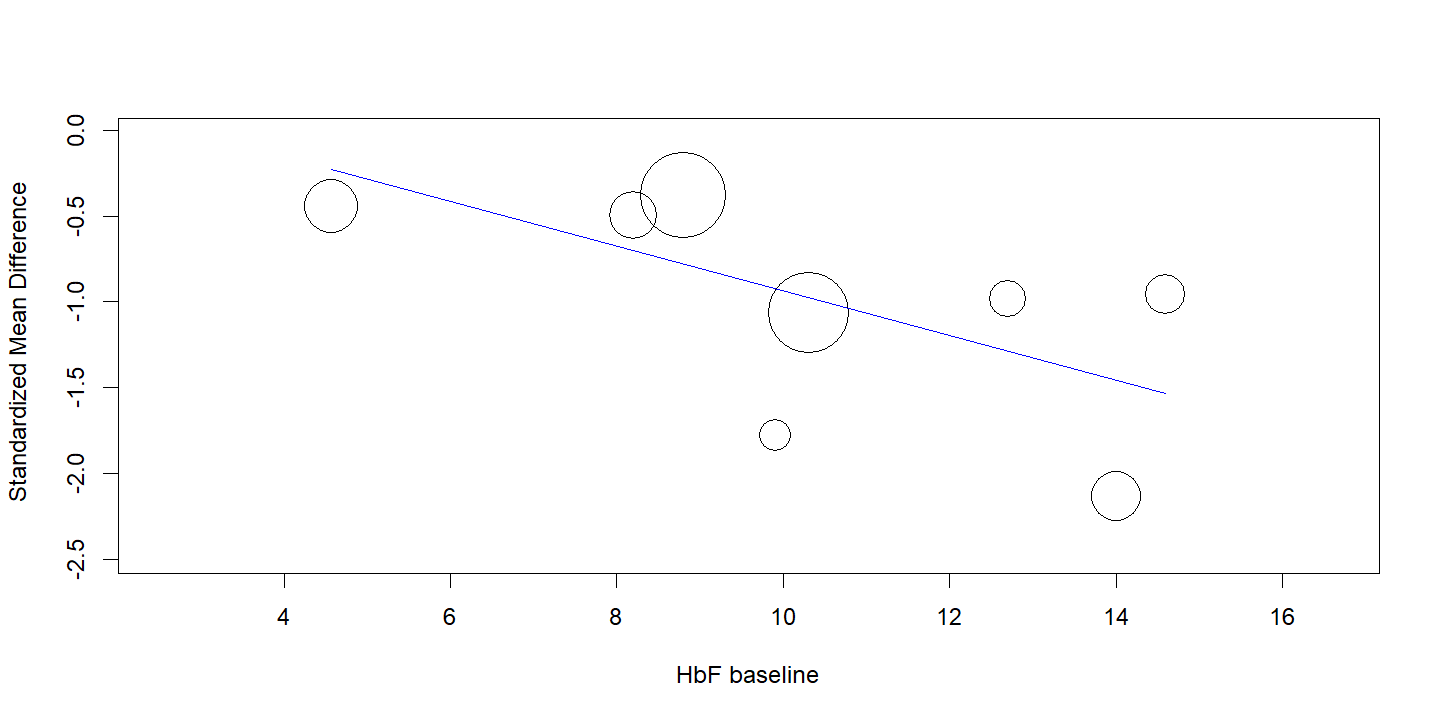

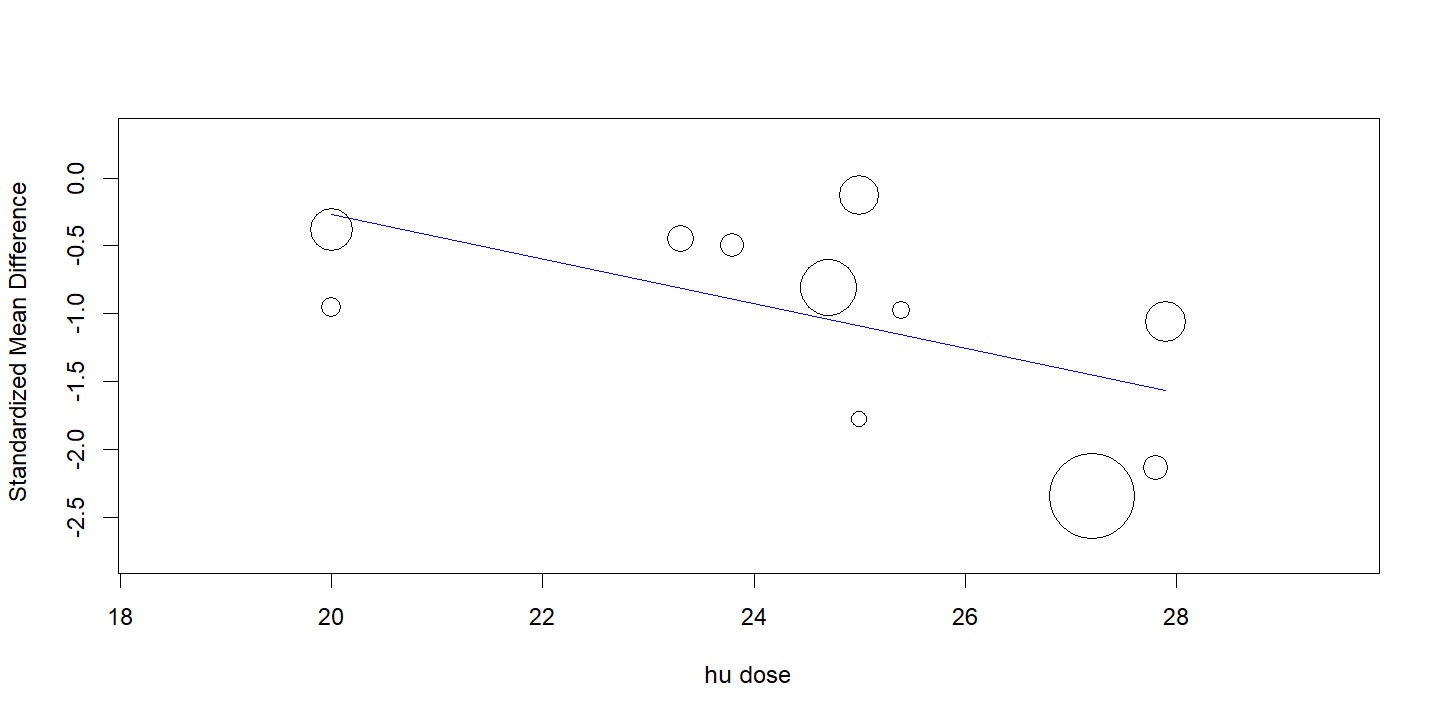

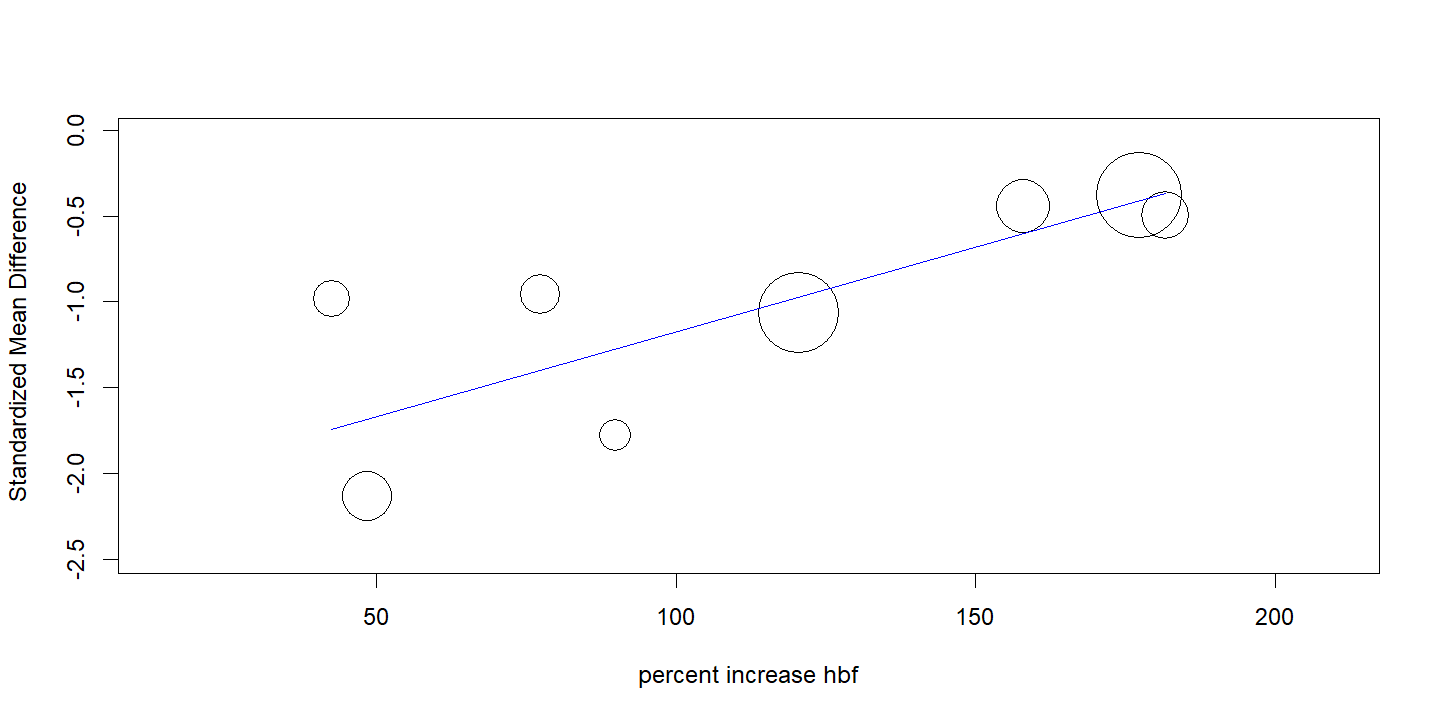

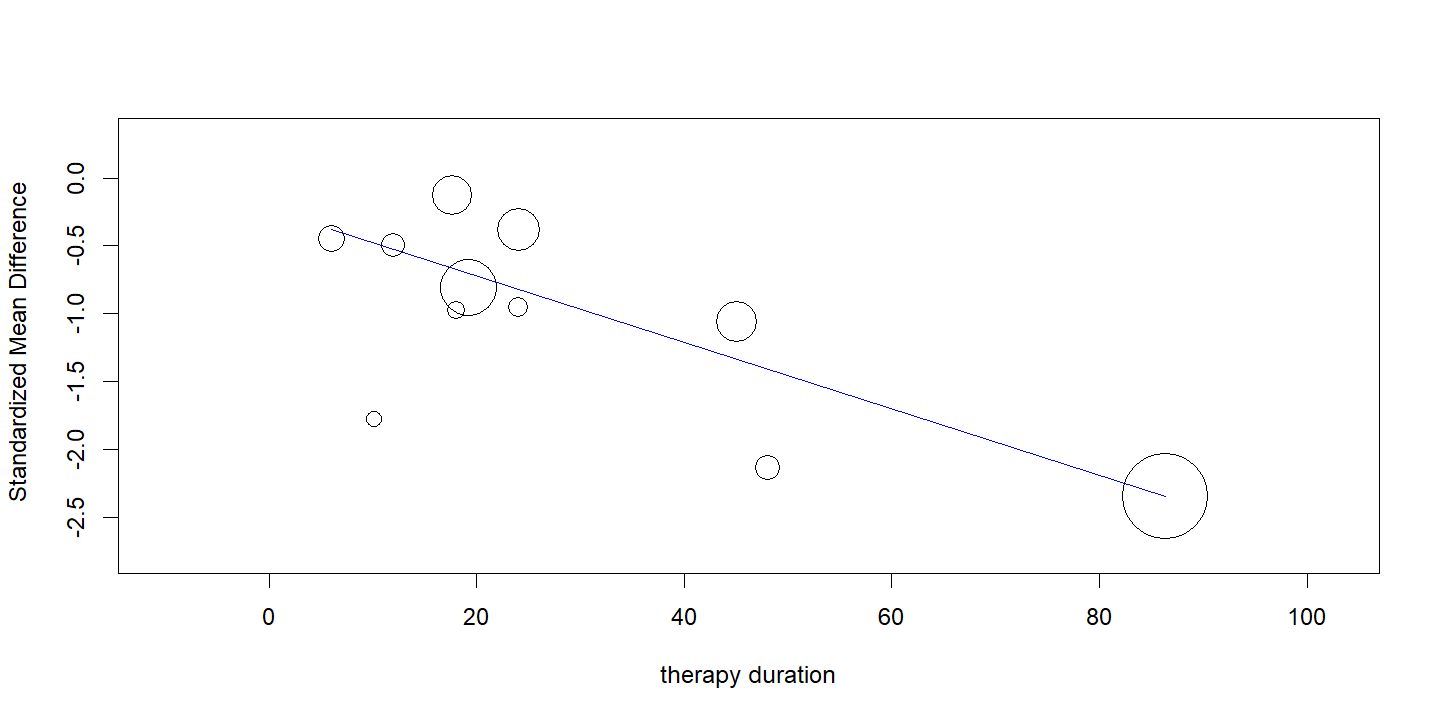


1. **For albuminuria in SCD patients**


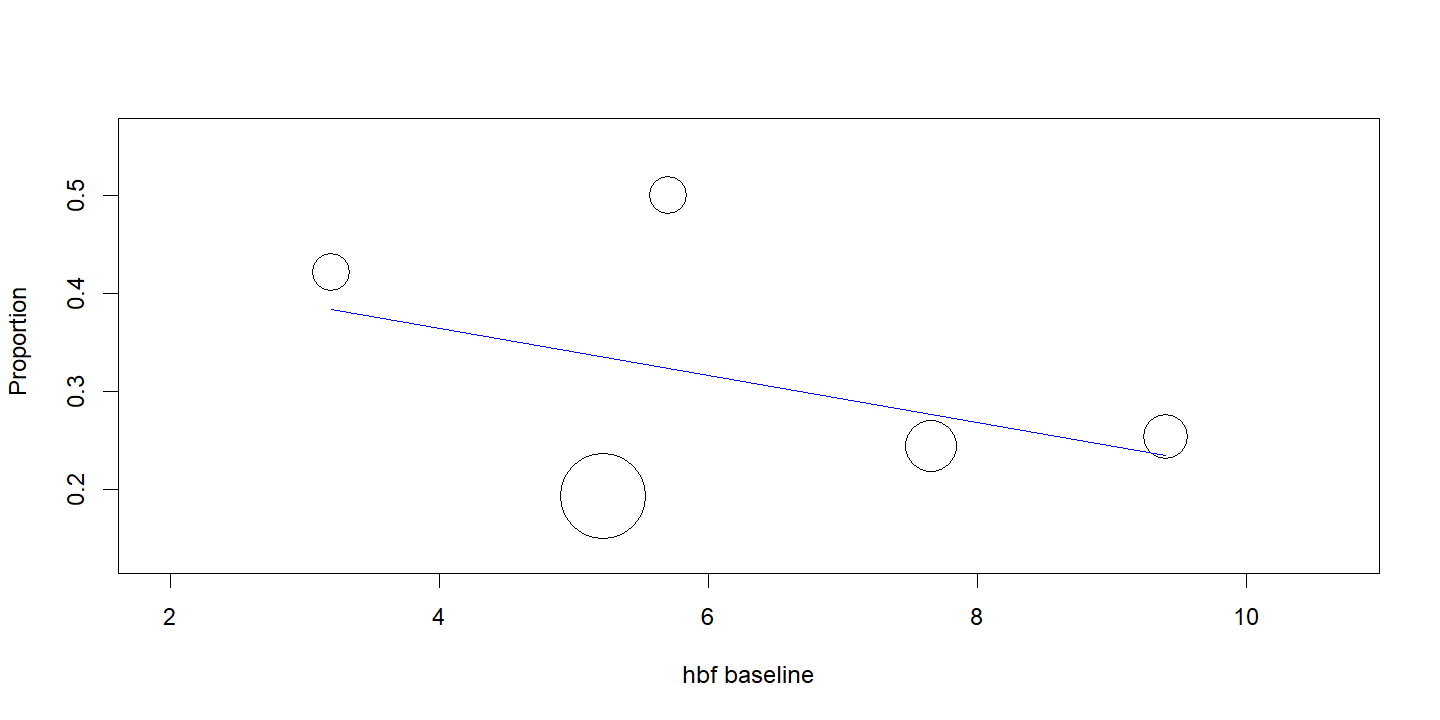

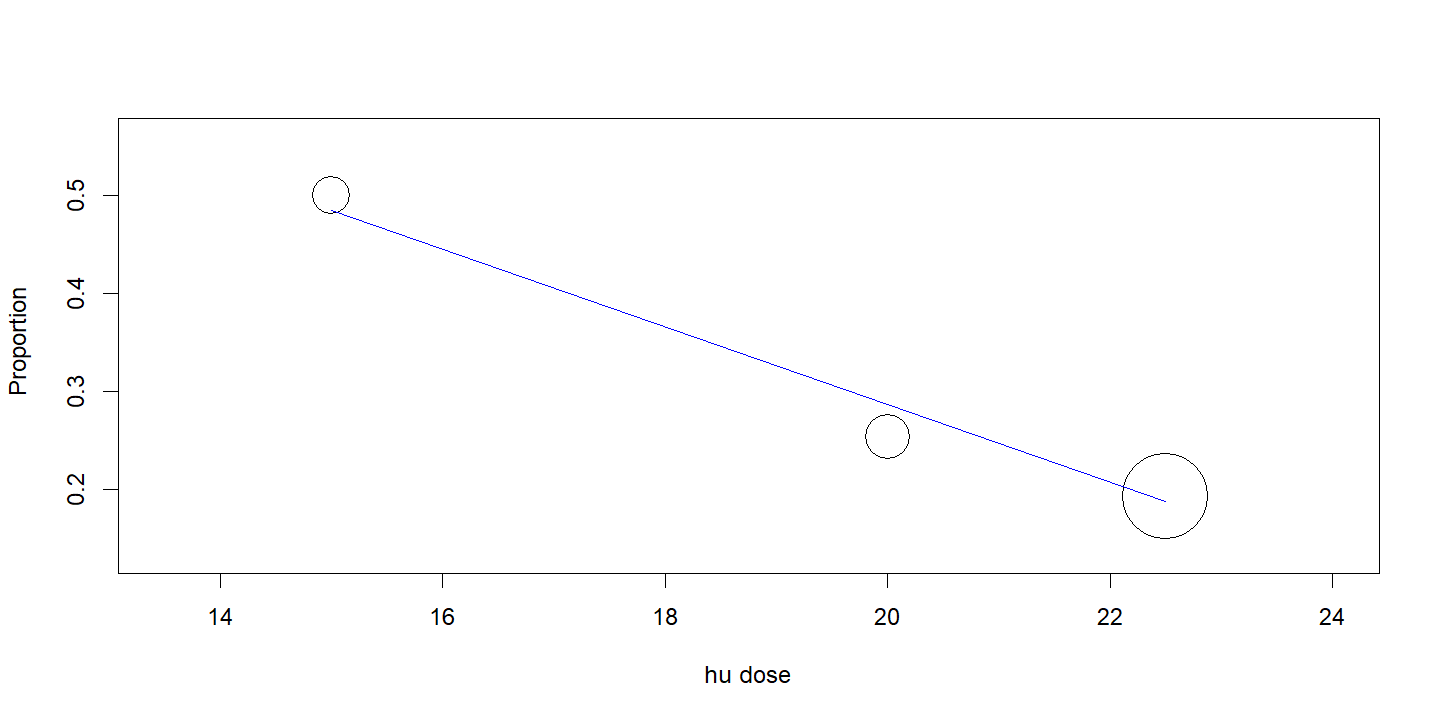

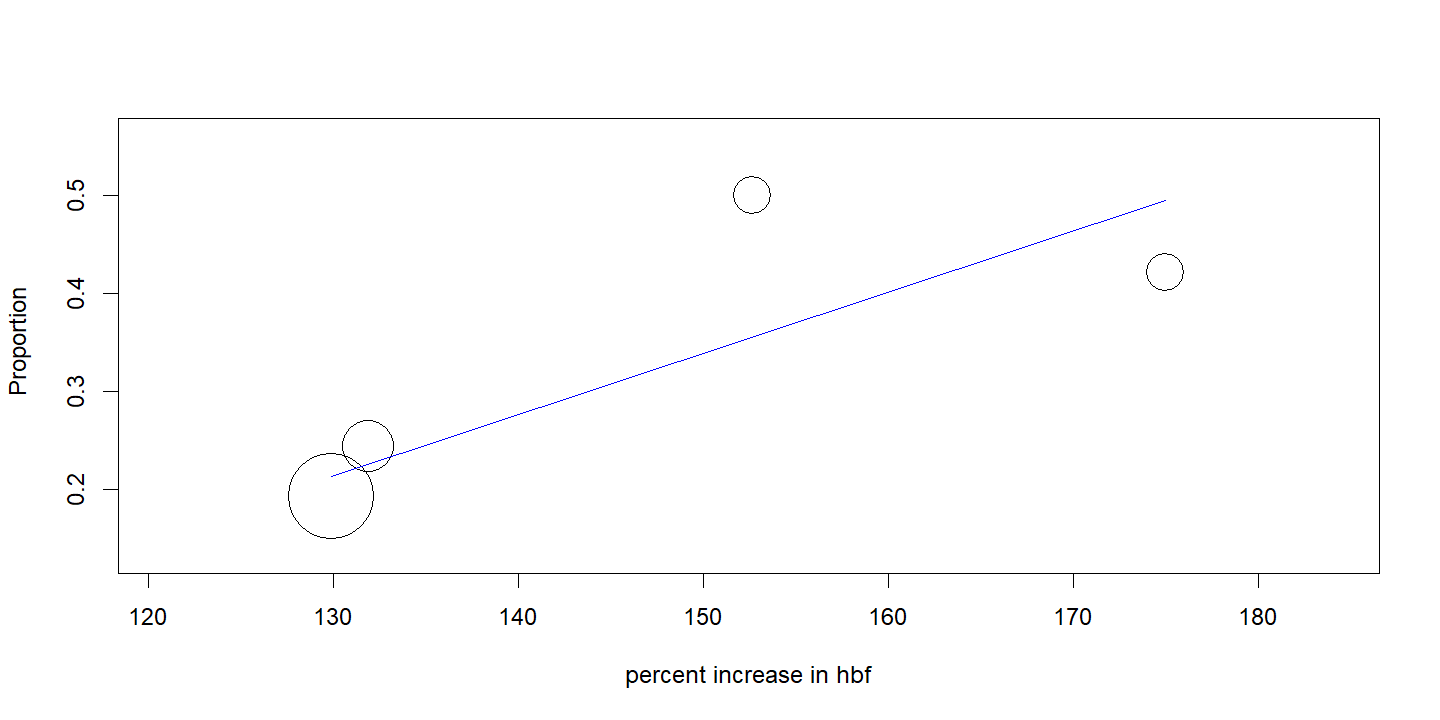

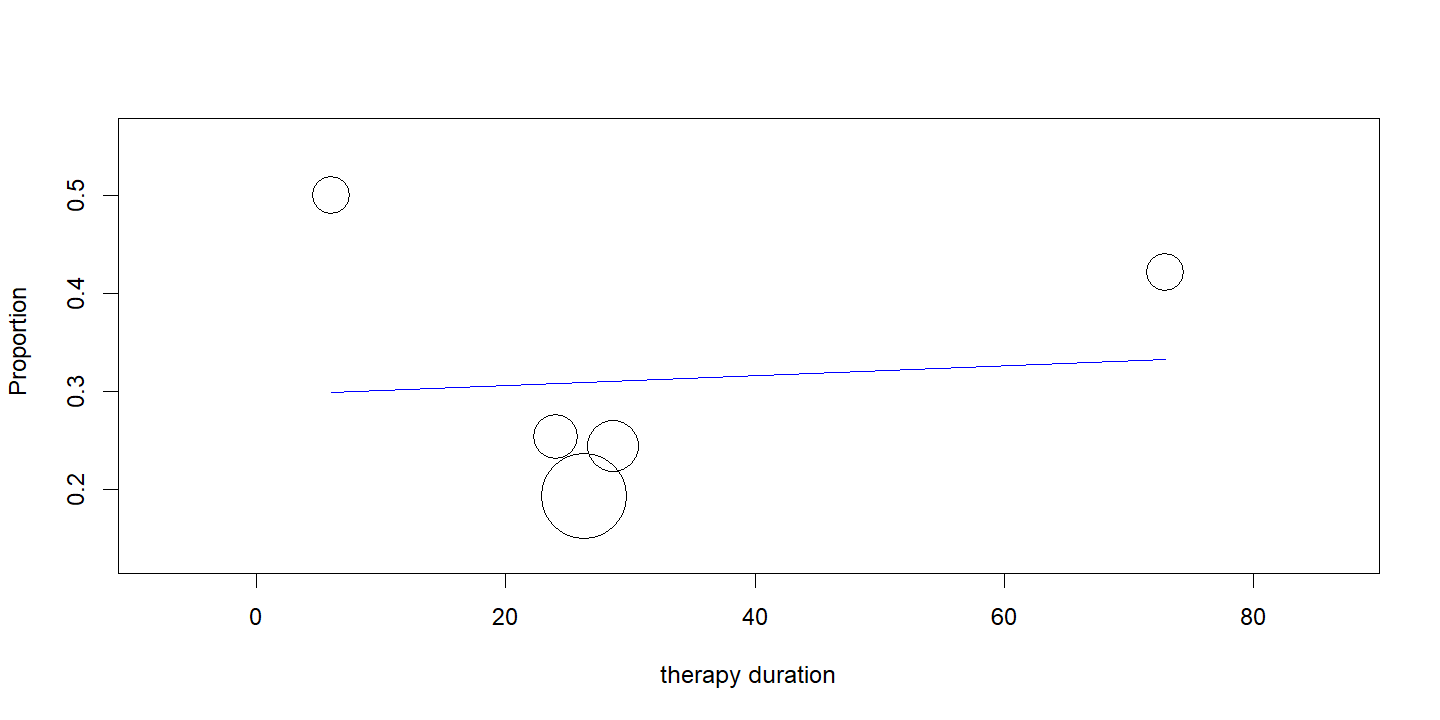


1. **For Tricuspid Regurgitant Velocity in SCD patients**


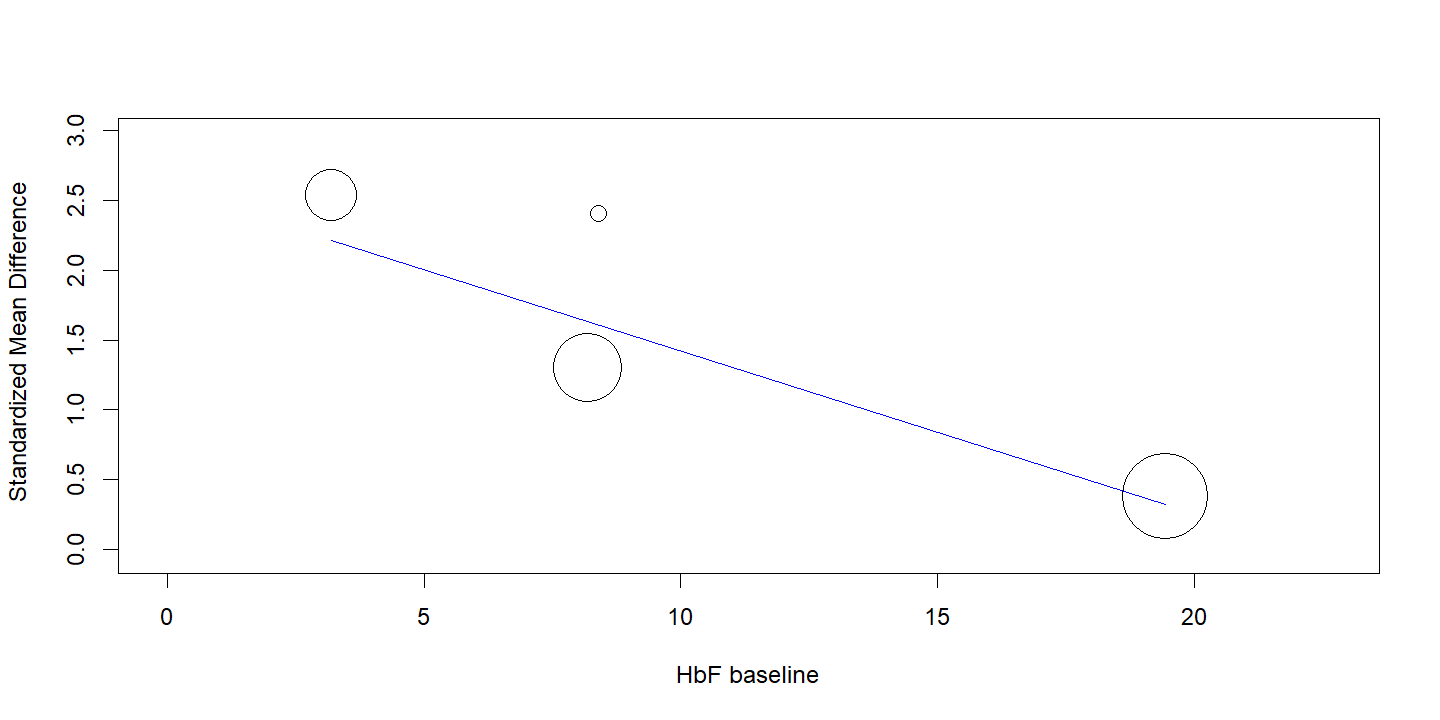

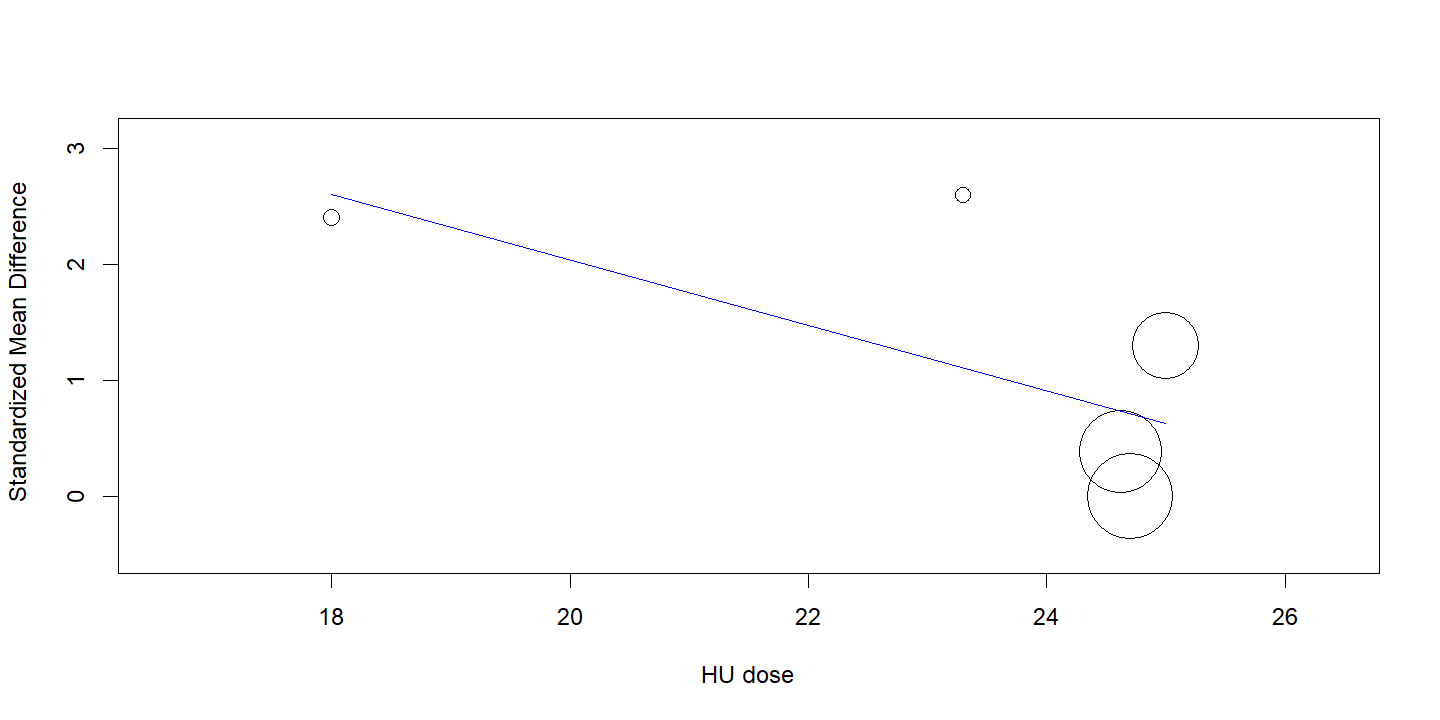

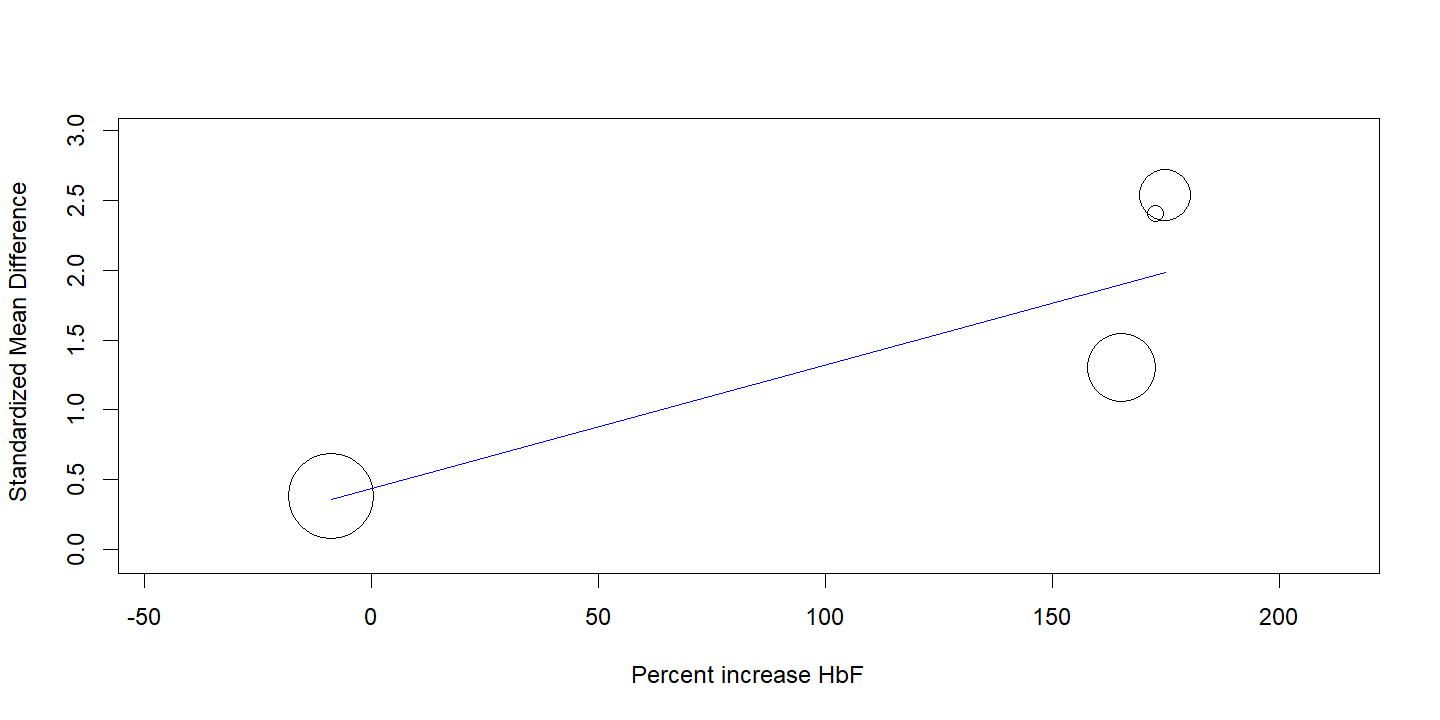

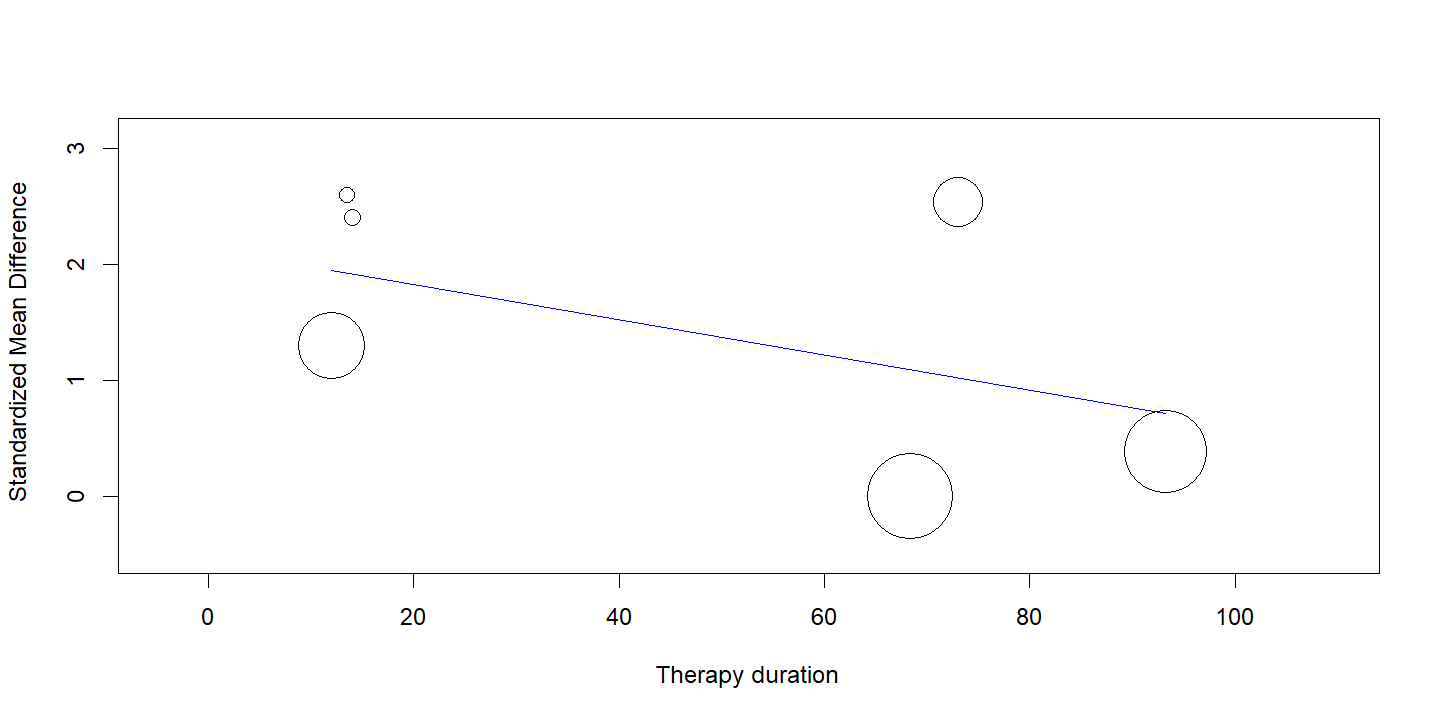


**Appendix V:Funnel Plots and Sensitivity analysis**

1. **For TCD velocity in SCD patients****^1^**^–^**^12^**


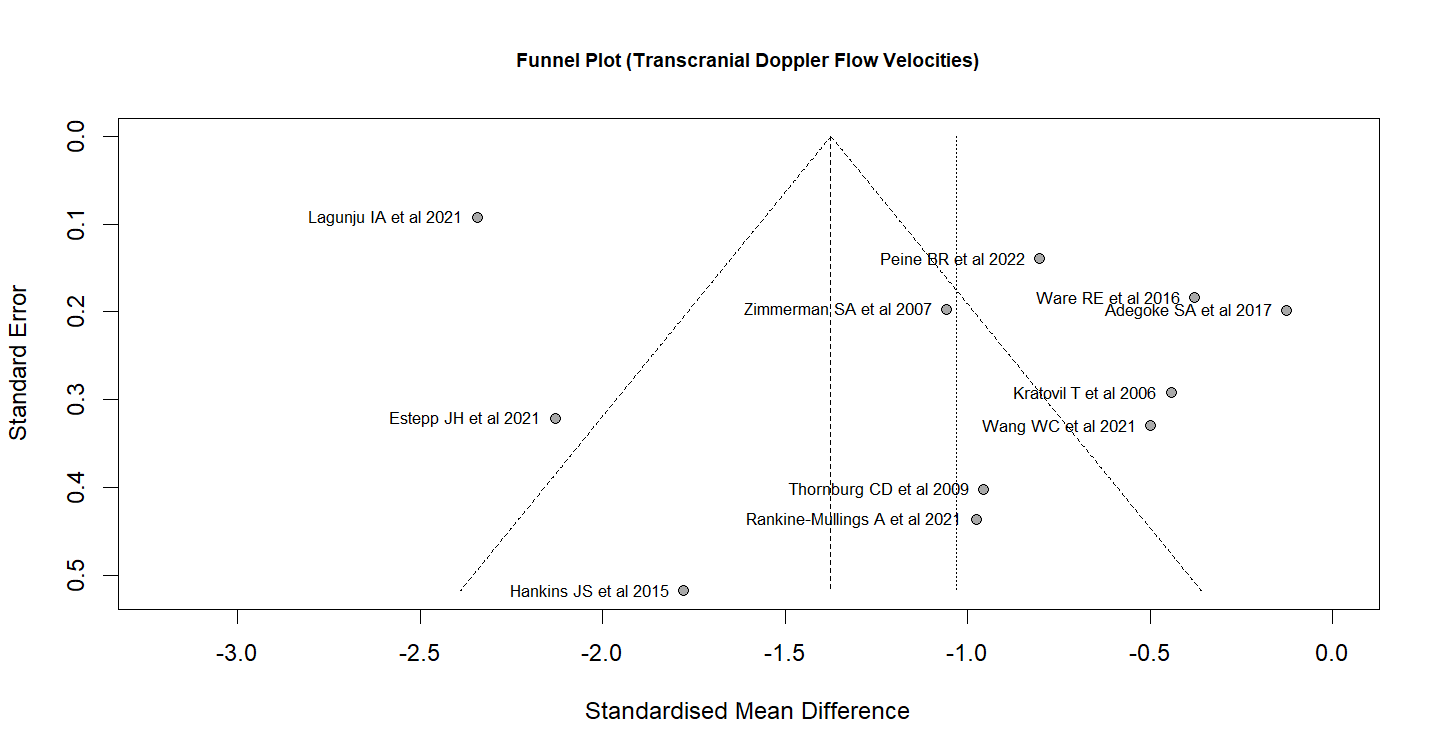


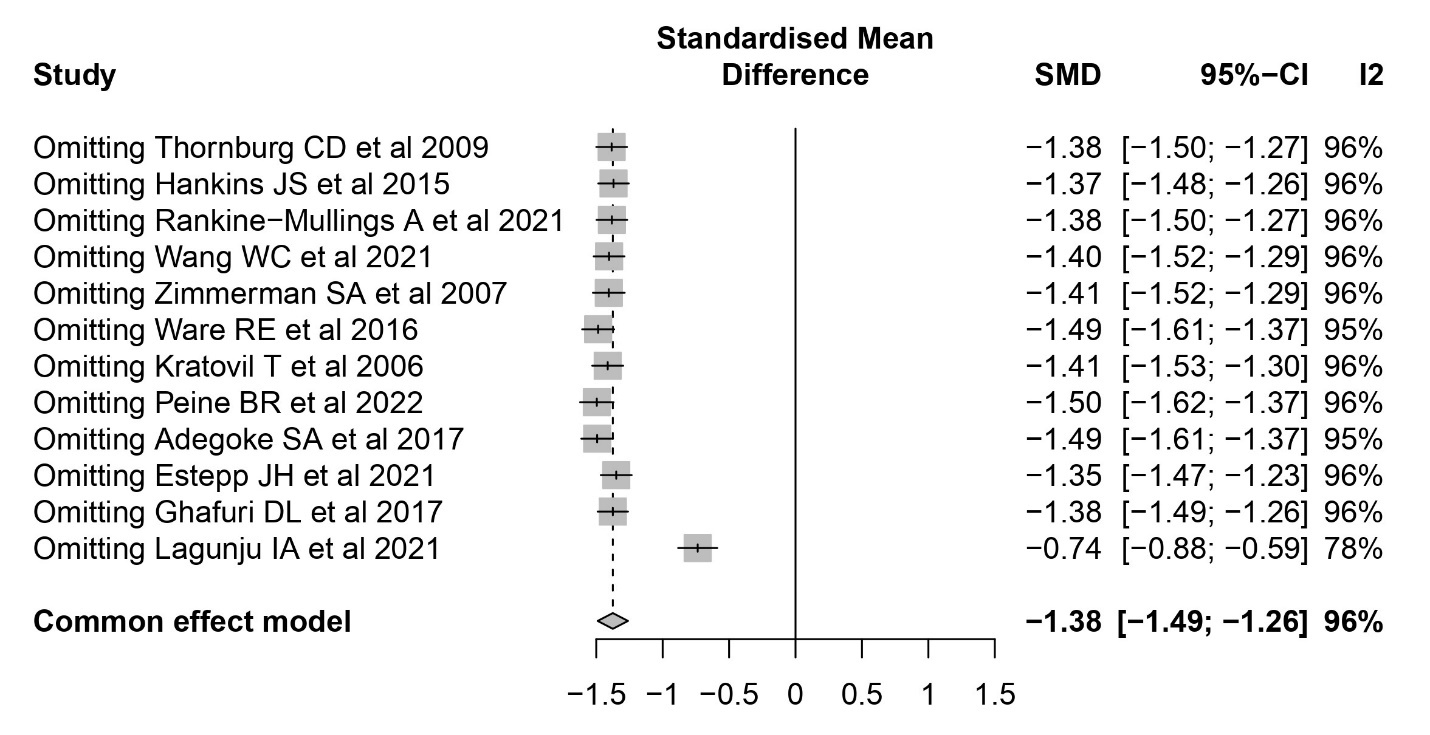


1. **For TRV in SCD patients****^13^**^–^**^19^**


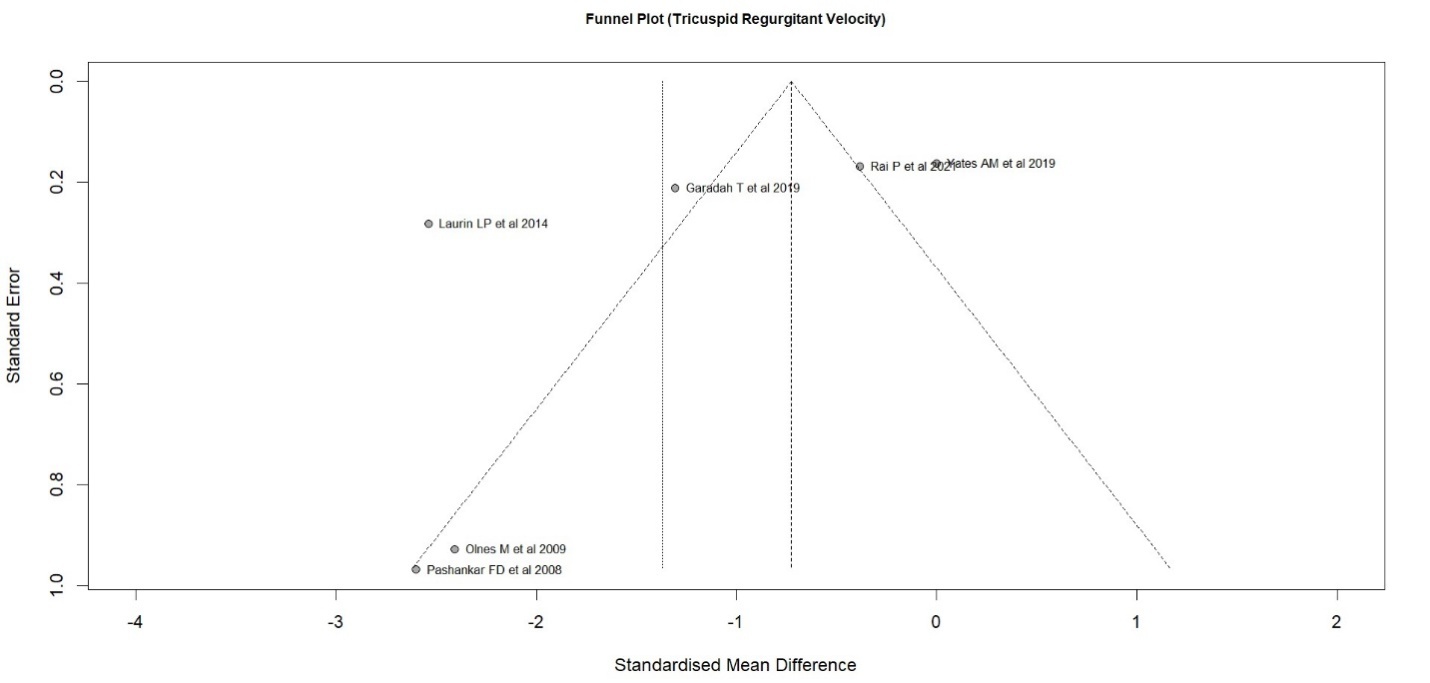


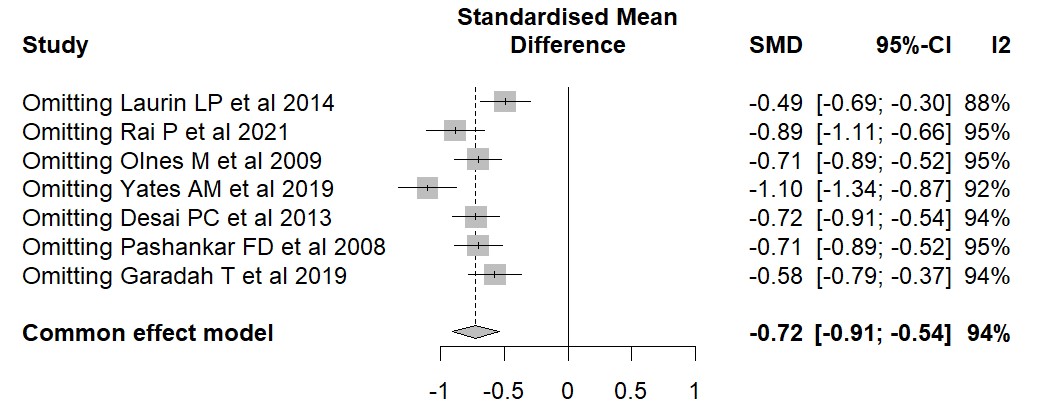


1. **For Serum creatinine levels in SCD patients****^13, 20^**^–^**^26^**


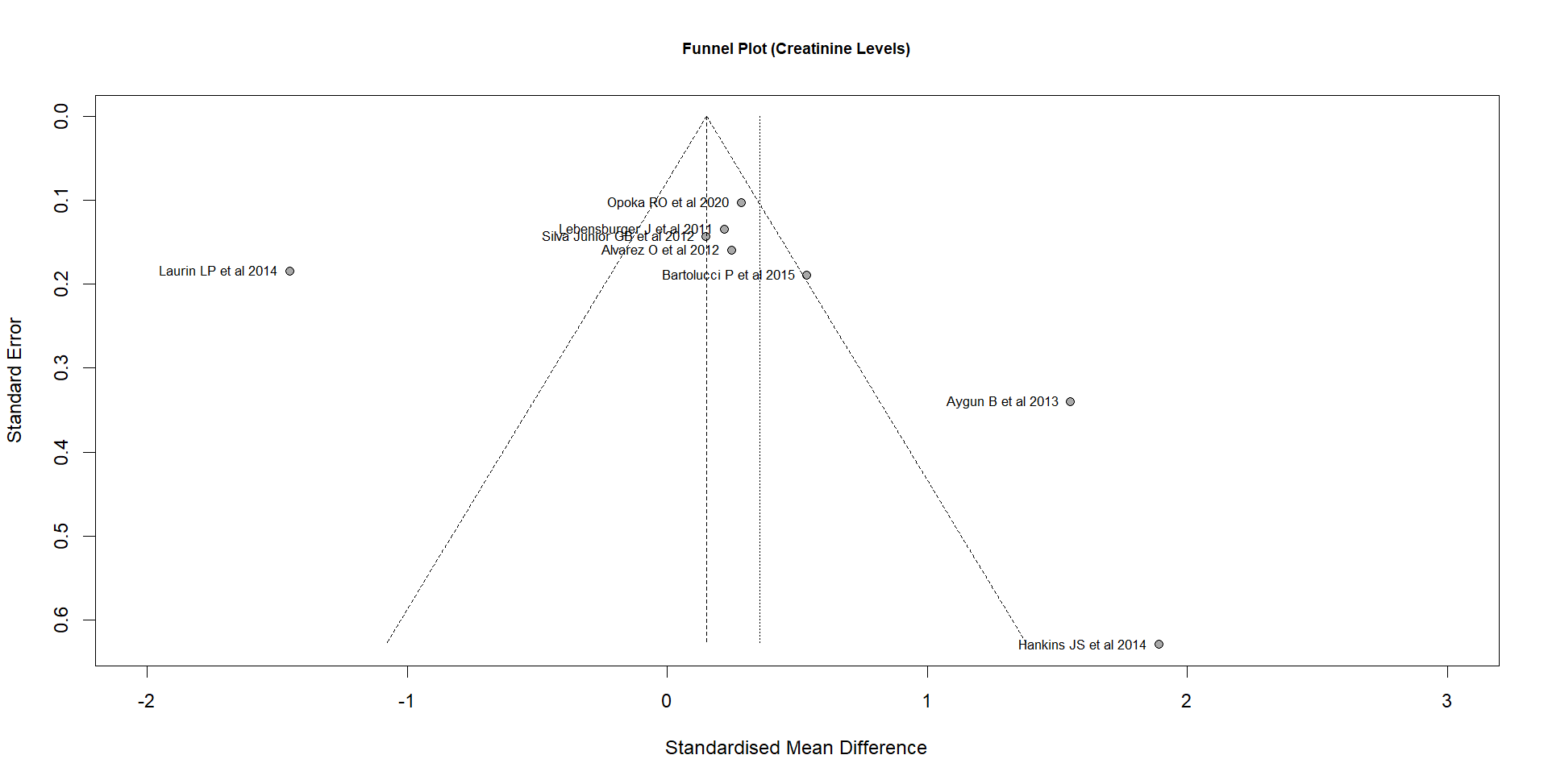


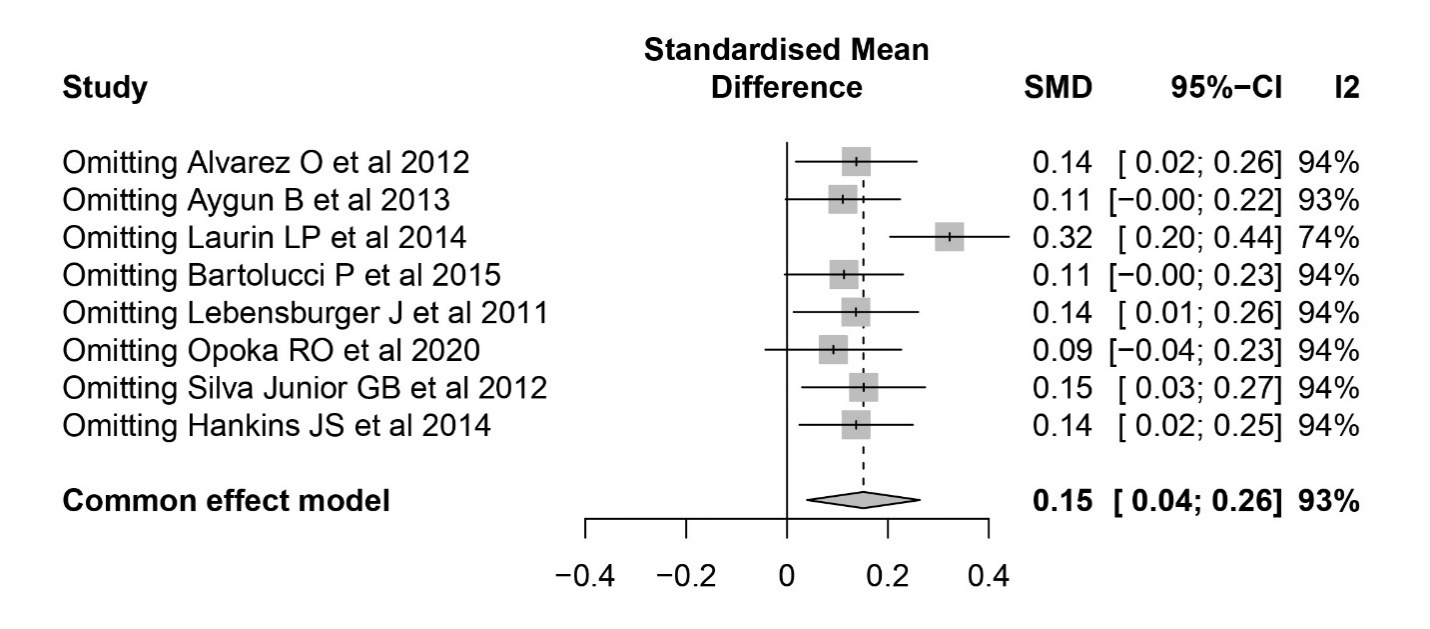


1. **For Urinary Albumin levels in SCD patients****^13, 24, 25, 27^**^–^**^29^**


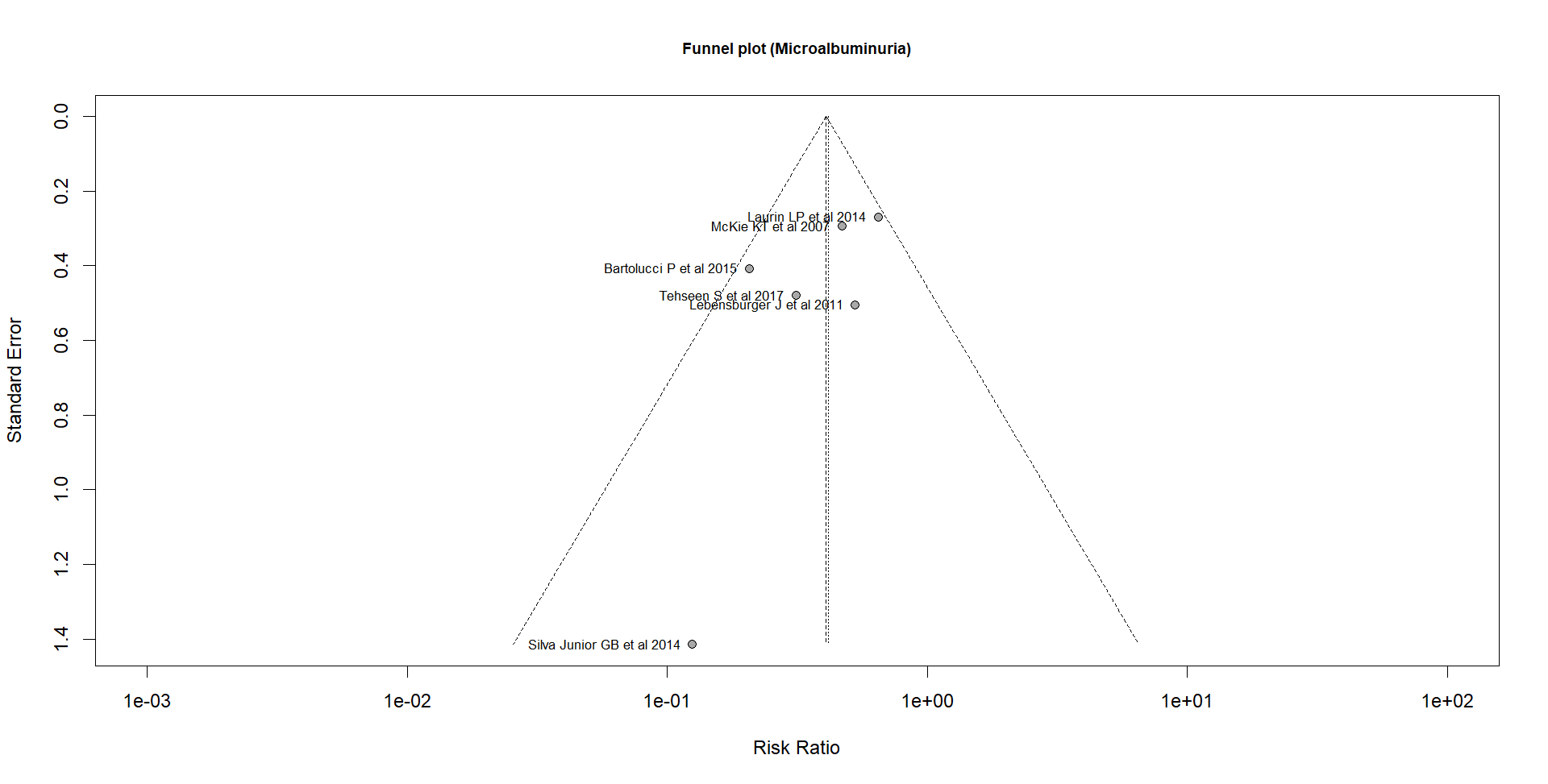


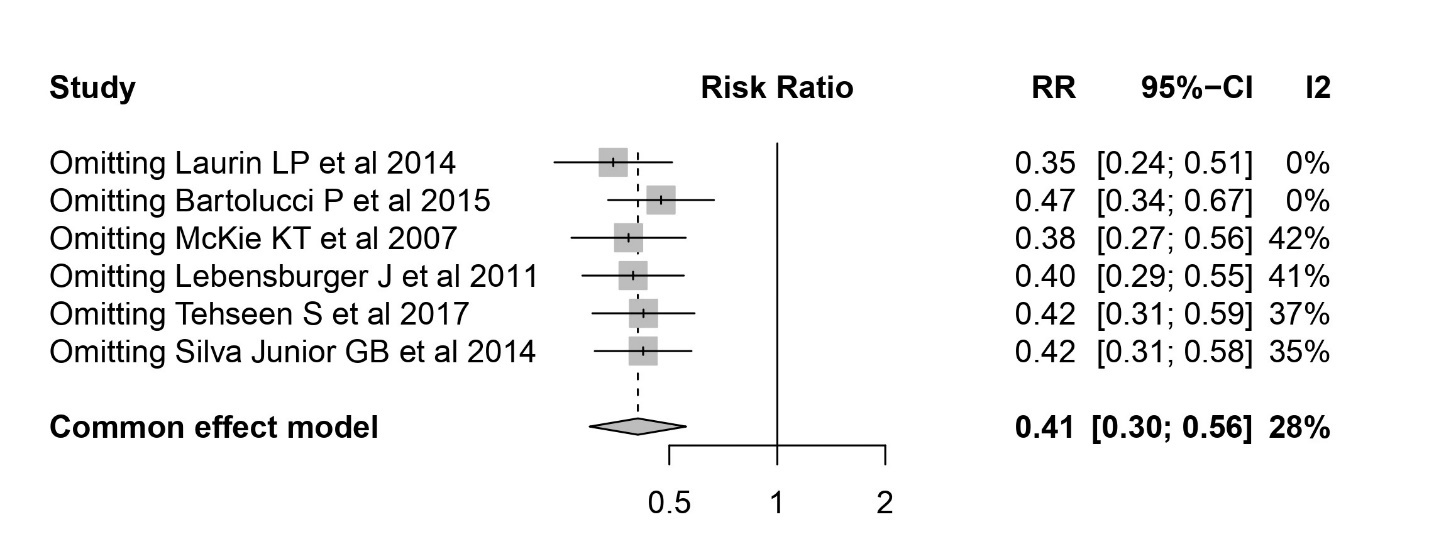


**Appendix VI:Sub Group Analysis:**

1. **For TCD^1^**^–^**^12^**

| **Estimate** | **Lower bound** | **Upper bound** | **Z** | **p-Value** |
| --- | --- | --- | --- | --- |
| -1·032 | -1·4872 | -0·5767 | -4·44 | < 0·0001 |
| **Heterogeneity** | | | | |
| **τ^2^** | **Q(df=10)** | **Het. p-Value** | **I^2^** |  |
| 0·5066 | 223·57 | < 0·001 | 95·50% |  |

**
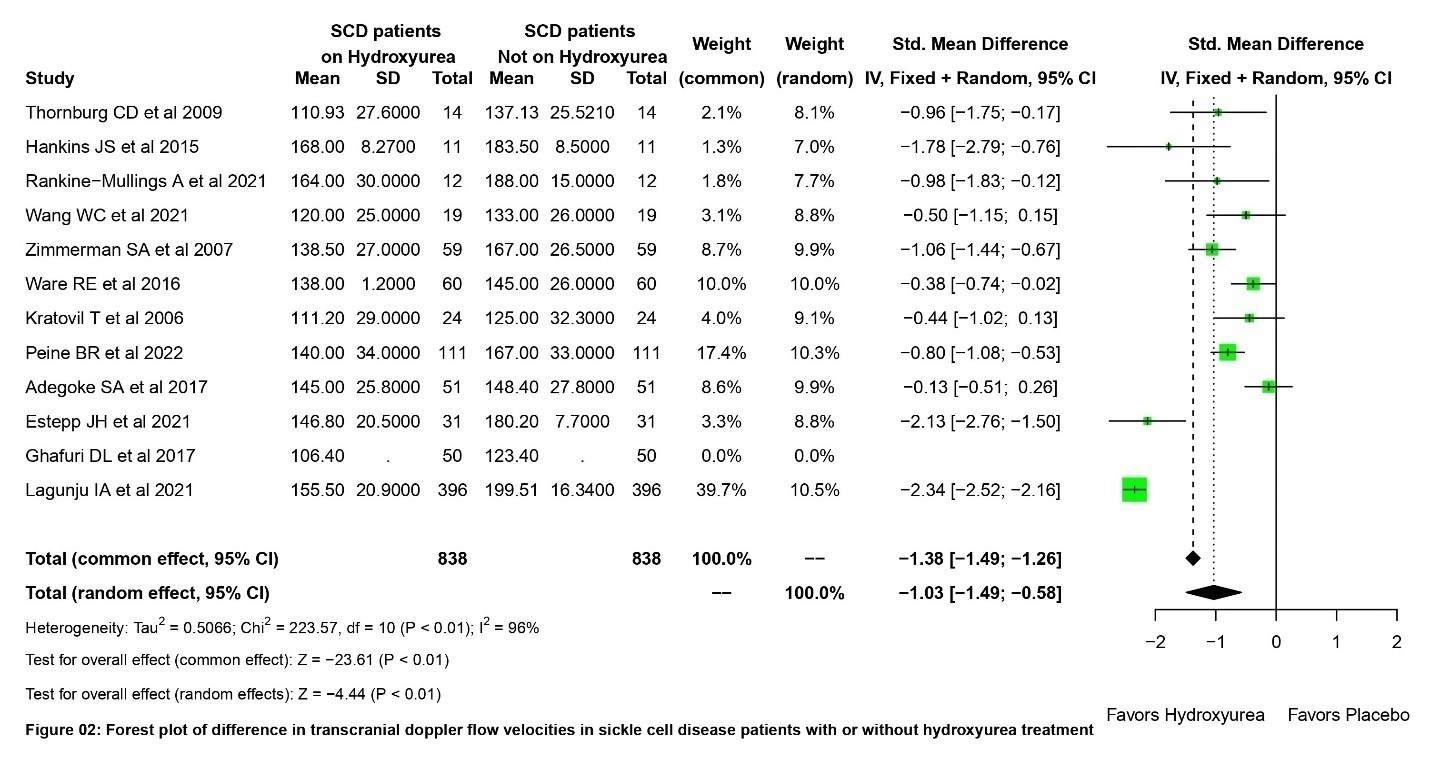
**

**HbF Subgroup Analysis for TCD**

Continuous Random-Effects Model

Metric: Standardized Mean Difference

Model Results

| **Subgroup** | **Estimate** | **Lower bound** | **Upper bound** | **τ^2^** | **p-Value** |
| --- | --- | --- | --- | --- | --- |
| **HbF>10%** | -1·2955 | -1·8602 | -0·7309 | 0·2191 | - |
| **HbF<10 %** | -0·5944 | -0·9972 | -0·1917 | 0·0755 | - |
| **Overall** | -0·9824 | -1·4245 | -0·5403 | 0·2955 | <0·0001 |
| **Heterogeneity** | | **Q** | **df** | **Het. p-Value** | |
| **Difference between groups** | | 3·92 | 1 | 0·0476 | |


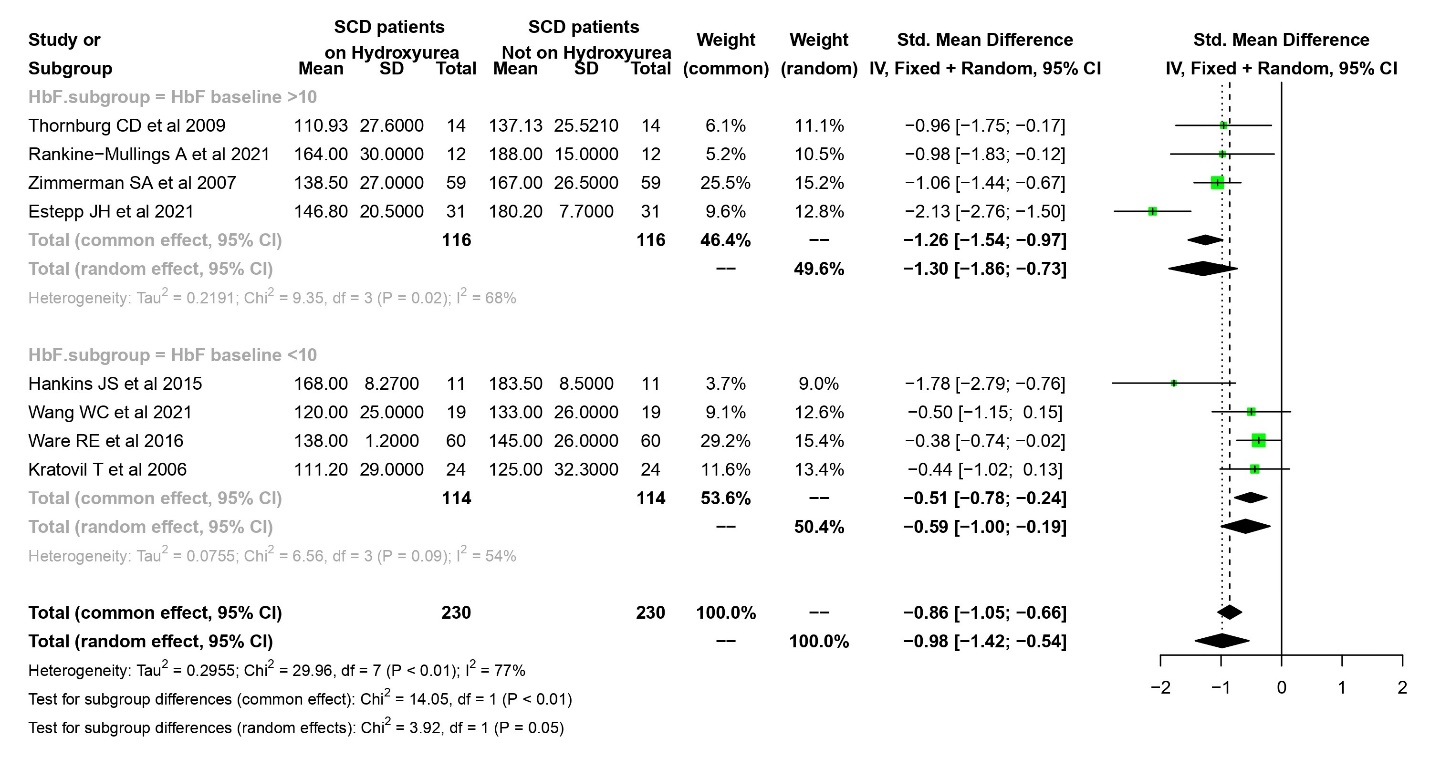


**HU Therapy Duration Subgroup Analysis for TCD**

Continuous Random-Effects Model

Metric: Standardized Mean Difference

Model Results

| **Subgroup** | **Estimate** | **Lower bound** | **Upper bound** | **τ^2^** | **p-Value** |
| --- | --- | --- | --- | --- | --- |
| **HU >18months** | -1·2792 | -1·9208 | -0·6377 | 0·5861 | - |
| **HU <18months** | -0·6353 | -1·1213 | -0·1493 | 0·1879 | - |
| **Overall** | -1·032 | -1·4872 | -0·5767 | 0·5066 | <0·0001 |
| **Heterogeneity** | | **Q** | **df** | **Het. p-Value** | |
| **Difference between groups** | | 2·46 | 1 | 0·1169 | |


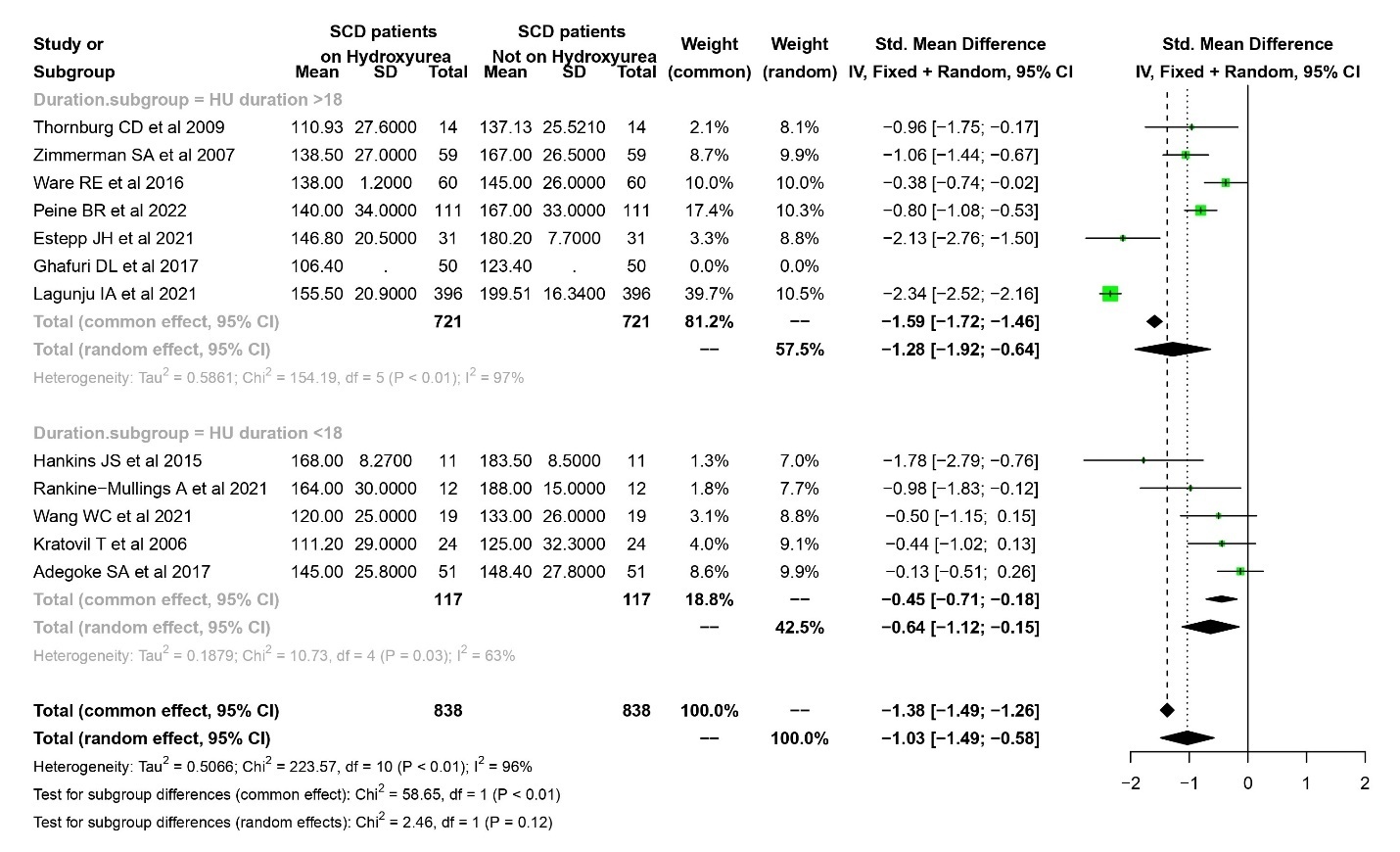


1. **For TRV^13^**^–^**^19^**

| **Estimate** | **Lower bound** | **Upper bound** | **Z** | **p-Value** |
| --- | --- | --- | --- | --- |
| -1·3702 | -2·3142 | -0·4261 | -2·84 | 0·0044 |
| **Heterogeneity** | | | | |
| **τ^2^** | **Q(df=5)** | **Het. p-Value** | **I^2^** |  |
| 1·1544 | 70·85 | <0·0001 | 93·70% |  |


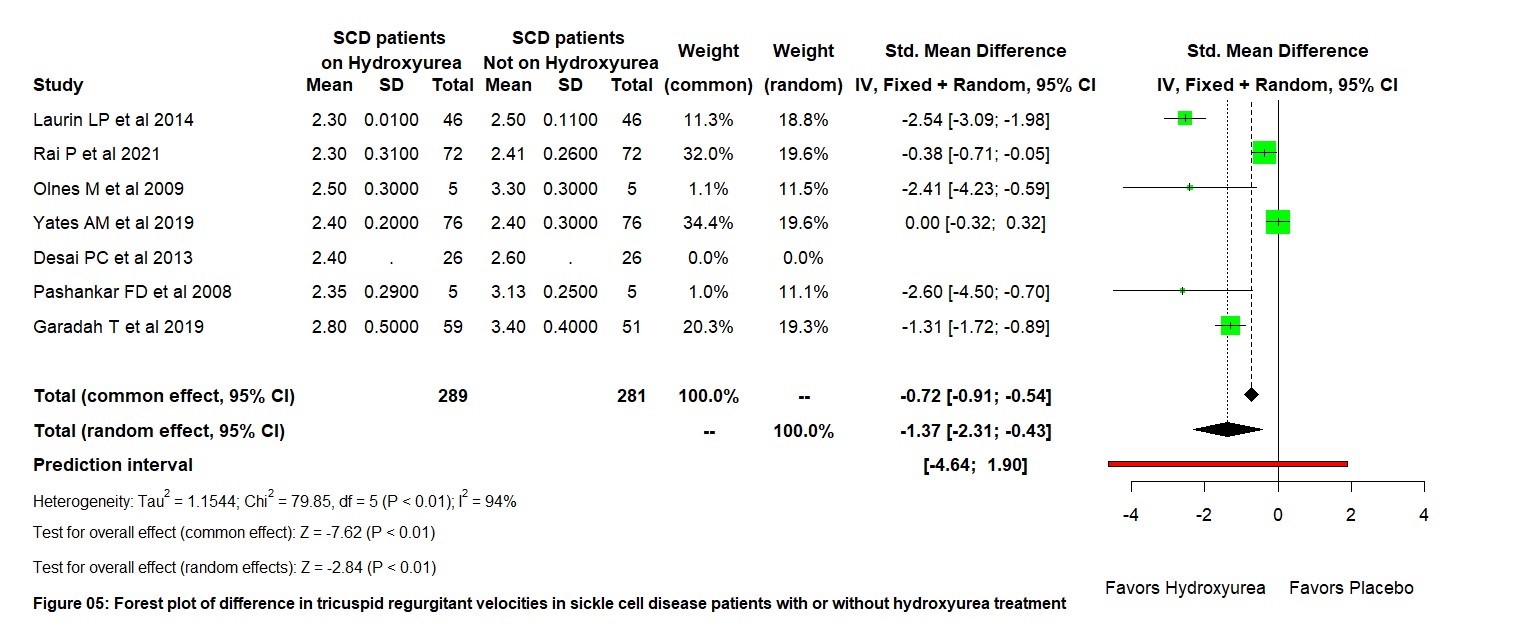


**HU Therapy Duration Subgroup Analysis for TRV**

Continuous Random-Effects Model

Metric: Standardized Mean Difference

Model Results

| **Subgroup** | **Estimate** | **Lower bound** | **Upper bound** | **τ^2^** | **p-Value** |
| --- | --- | --- | --- | --- | --- |
| **HU >18months** | -0·9591 | -2·4942 | 0·5761 | 1·7958 | - |
| **HU <18months** | -1·7405 | -2·6551 | -0·8259 | 0·2978 | - |
| **Overall** | -1·3702 | -2·3142 | -0·4261 | 1·1544 | 0·0044 |
| **Heterogeneity** | | **Q** | **df** | **Het. p-Value** | |
| **Difference between groups** | | 79·85 | 5 | <0·0001 | |


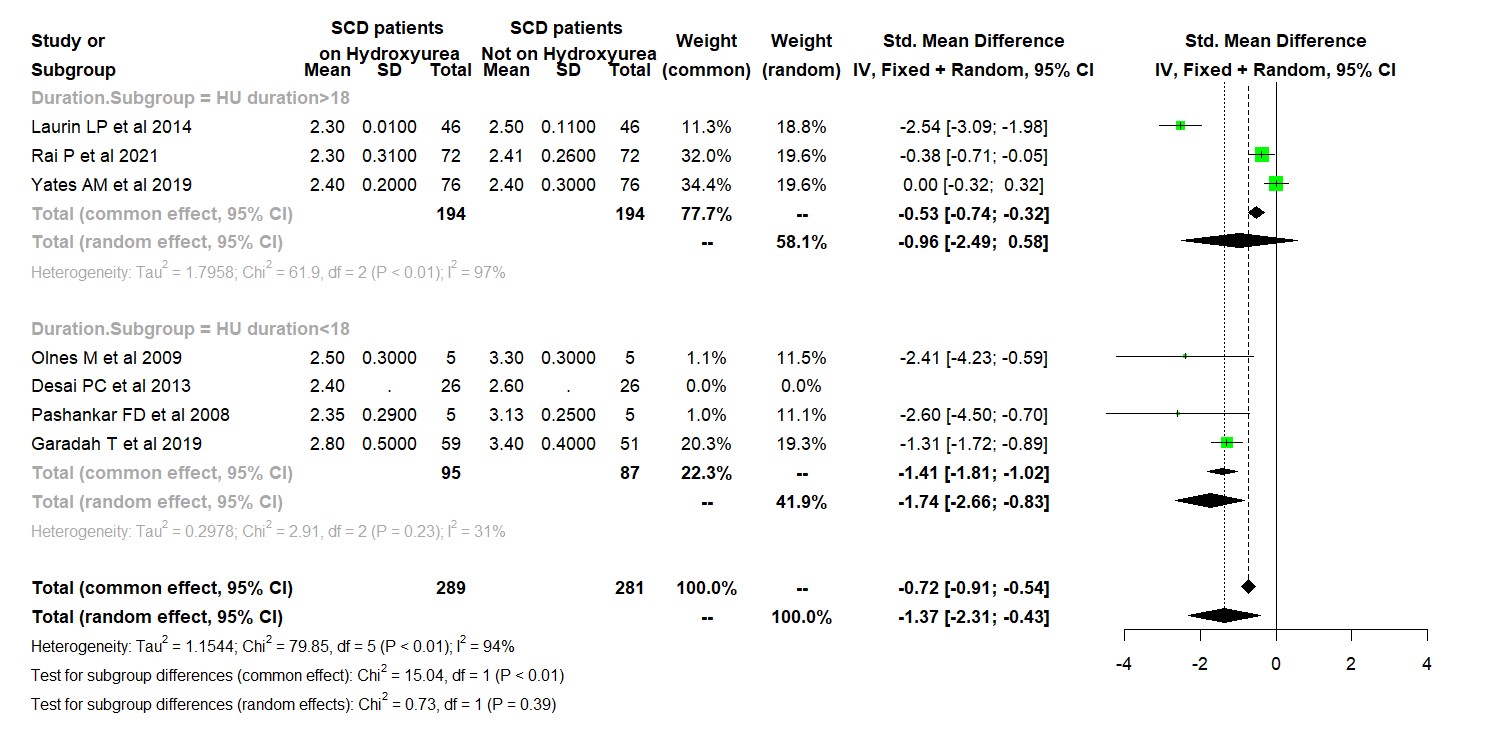


1. **For Serum Creatinine Levels^13, 20^**^–^**^26^**

| **Estimate** | **Lower bound** | **Upper bound** | **Z** | **p-Value** |
| --- | --- | --- | --- | --- |
| 0·3572 | -0·2963 | 1·0108 | 1·07 | 0·284 |
| **Heterogeneity** | | | | |
| **τ^2^** | **Q(df=7)** | **Het· p-Value** | **I^2^** |  |
| 0·8206 | 106·98 | <0·0001 | 93·50% |  |


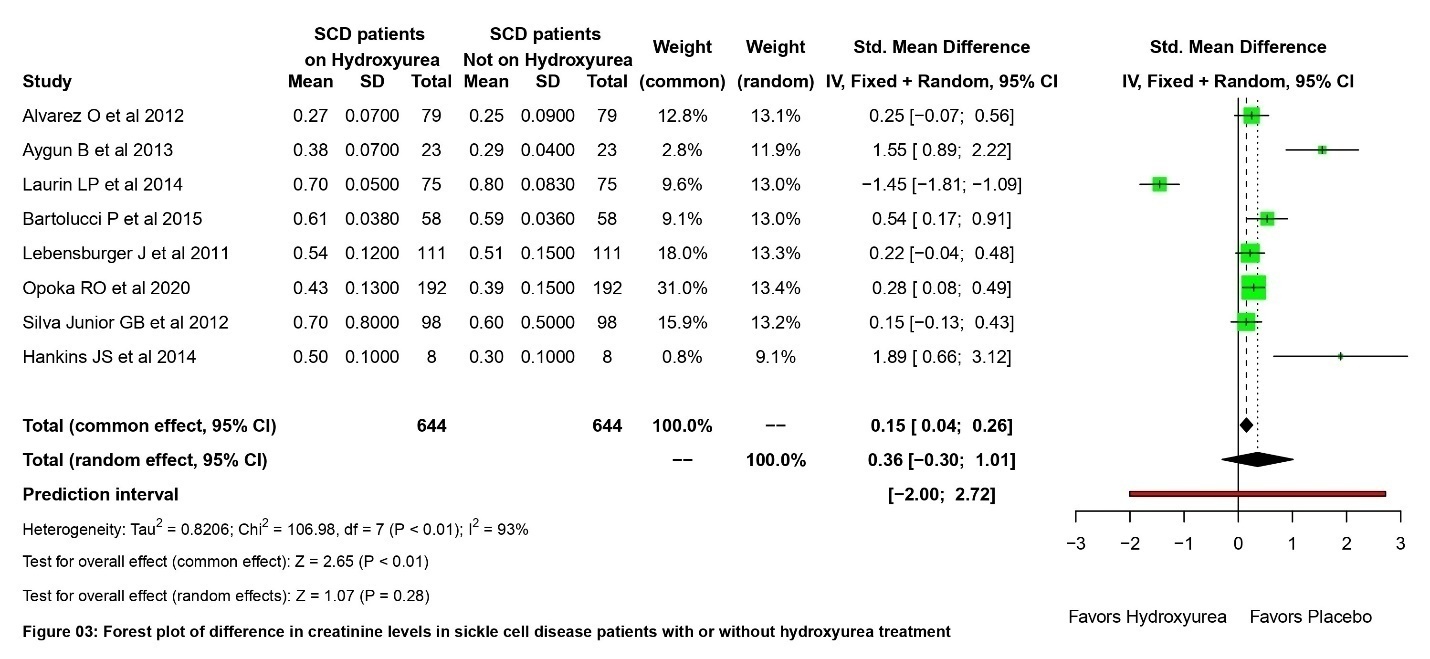


**HbF Subgroup Analysis for Creatinine**

Continuous Random-Effects Model

Metric: Standardized Mean Difference

Model Results

| **Subgroup** | **Estimate** | **Lower bound** | **Upper bound** | **τ^2^** | **p-Value** |
| --- | --- | --- | --- | --- | --- |
| **HbF>10%** | 0·8651 | 0·074 | 1·6561 | 0·5441 | - |
| **HbF<10 %** | -0·2301 | -1·4354 | 0·9753 | 1·1054 | - |
| **Overall** | 0·3995 | -0·3655 | 1·1644 | 0·9882 | 0·3061 |
| **Heterogeneity** | | **Q** | **df** | **Het. p-Value** | |
| **Difference between groups** | | 2·22 | 1 | 0·1365 | |


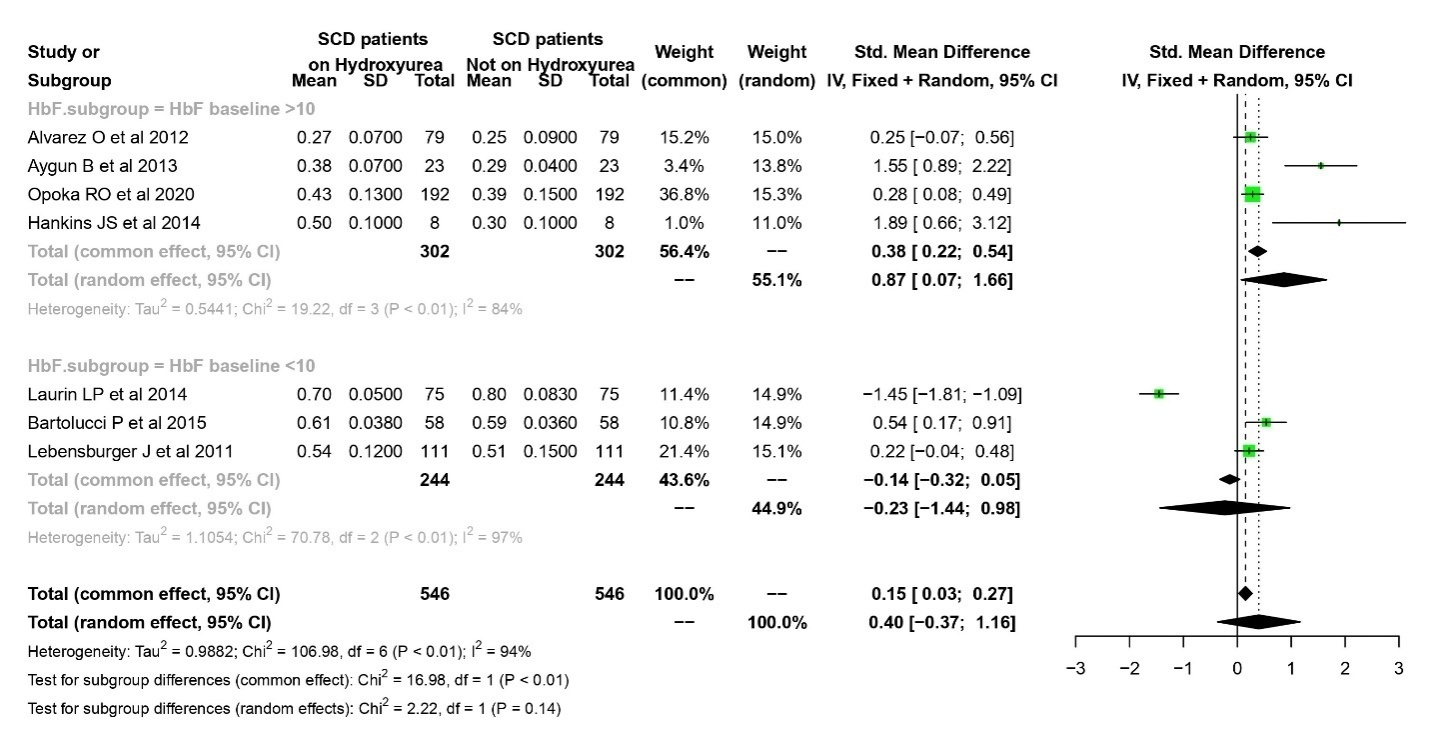


1. **For Urinary Albumin Levels^13, 24, 25, 27^**^–^**^29^**

| **Risk Ratio** | **Lower bound** | **Upper bound** | **Z** | **p-Value** |
| --- | --- | --- | --- | --- |
| 0·4156 | 0·276 | 0·6258 | -4·2 | <0·0001 |
| **Heterogeneity** | | | | |
| **τ^2^** | **Q(df=5)** | **Het. p-Value** | **I^2^** |  |
| 0·0832 | 6·96 | 0·2238 | 28·10% |  |


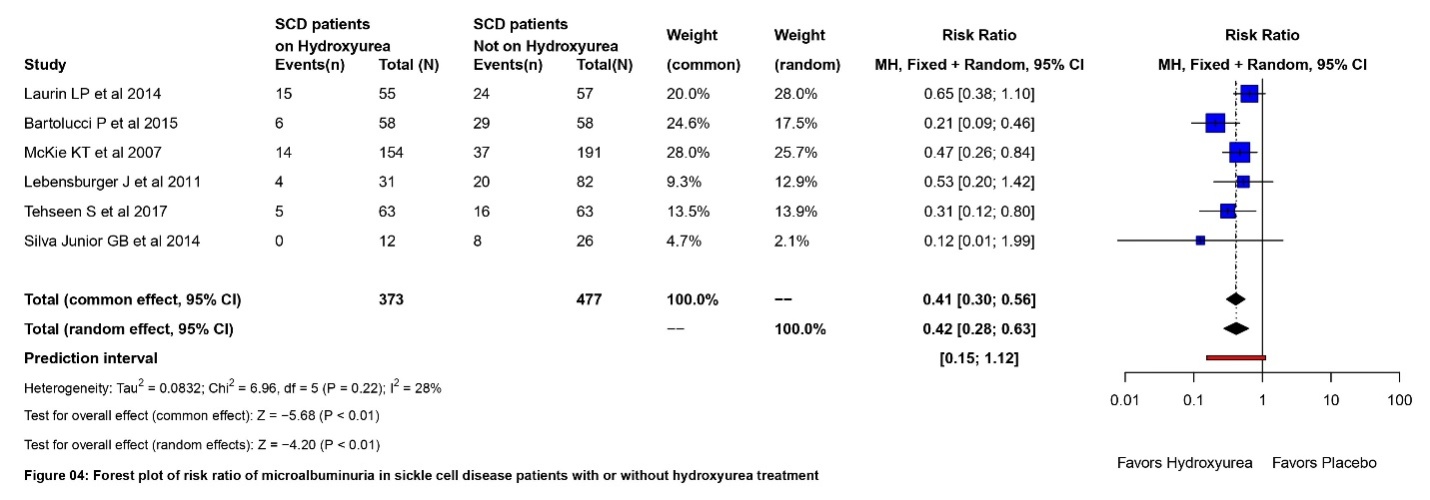


**Age Subgroup Analysis for Albumin**

Binary Random-Effects Model

Metric: Odds Ratio

Model Results

| **Subgroup** | **Risk Ratio** | **Lower bound** | **Upper bound** | **τ^2^** | **p-Value** |
| --- | --- | --- | --- | --- | --- |
| **Adults** | 0·3412 | 0·1266 | 0·9196 | 0·4557 | - |
| **Children** | 0·4396 | 0·2829 | 0·6830 | 0 | - |
| **Overall** | 0·4156 | 0·276 | 0·6258 | 0·0832 | <0·0001 |
| **Heterogeneity** | | **Q** | **df** | **Het. p-Value** | |
| **Difference between groups** | | 0·21 | 1 | 0·6471 | |


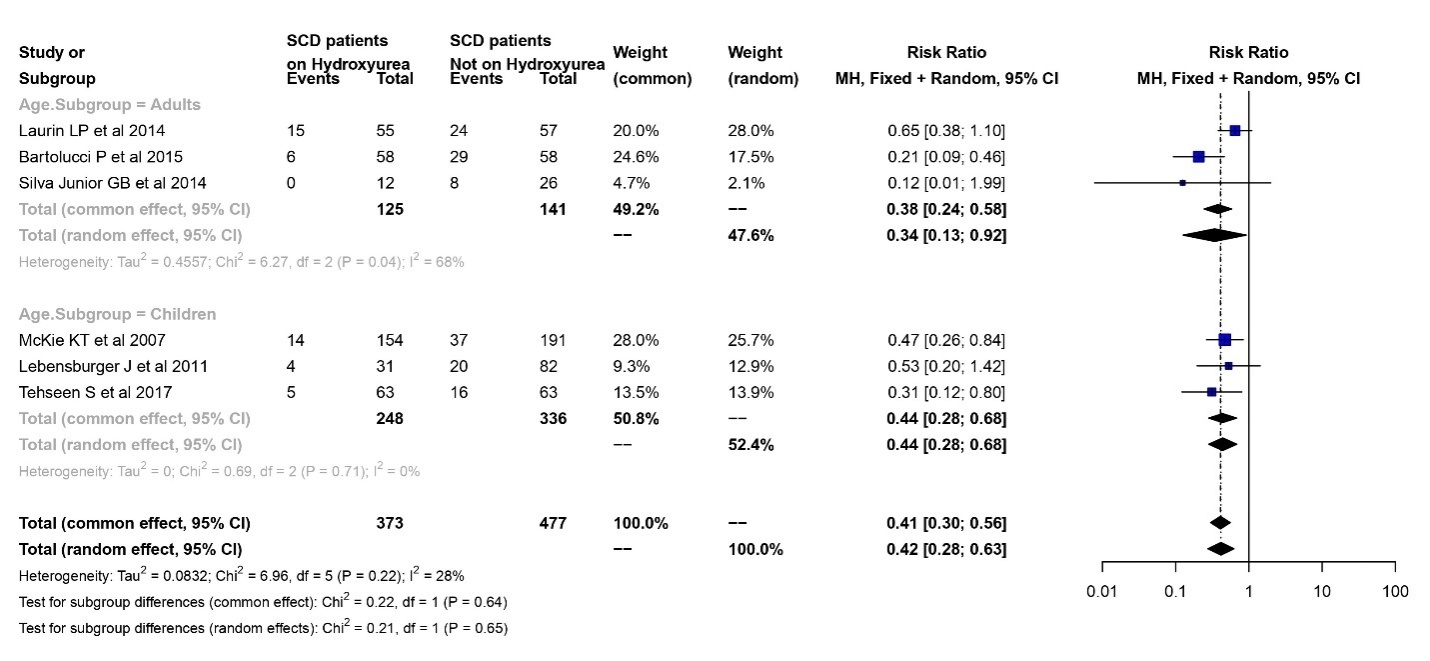


**Appendix VII: Full length studies excluded with reasons for exclusion**

| **Sr.No.** | **Author and Year** | **Country** | **Study Design** | **Number of cases screened** | **Justification for excluding the study** |
| --- | --- | --- | --- | --- | --- |
| 1 | Al-Jam'a AH et al, 2002^46^ | Saudi Arabia | Randomized controlled trial | 36 | Effect of HU on vaso-occlusive crises was assessed but not organ dysfunction. |
| 2 | Alkhunaizi AM et al, 2018^47^ | Saudi Arabia | Prospective study | 72 | This was a prospective cross-sectional study that measured the prevalence of microalbuminuria in adult SCD patients; there was no mention of microalbuminuria in SCD patients who were on HU therapy and not on HU therapy. |
| 3 | Ataga KI et al, 2022^48^ | USA | Retrospective study | 610 | This study doesn’t mention the GFR values in SCD patients who were on HU therapy and not on HU therapy. |
| 4 | Aygun B et al, 2012^49^ | USA | Prospective study | 340 | The study is on chronic transfusion to prevent primary stroke in SCD patients and with abnormal TCD. |
| 5 | Ballas SK et al, 2014^50^ | USA | Case report study | 1 case | This is a case report of a SCD patient having primary stroke treated with HU, but the effect of HU on stroke prevention is not clearly mentioned. |
| 6 | Bernaudin F et al, 2016^51^ | France | Prospective study | 309 | No mention of effect of HU on TCD velocity and incidence of stroke in the article. |
| 7 | Brousse V et al, 2012^52^ | Paris, UK | Retrospective study | 7 | No mention of effect of HU on TCD velocity and incidence of stroke in the article. |
| 8 | Daak AA et al, 2018^53^ | USA | Randomized controlled trial | 75 | Measurement of organ dysfunction was not the outcome of the study and not mentioned in the study. |
| 9 | Dedeken L et al,2014^54^ | Belgium | Cross-sectional study | 46 | No mention of effect of HU on TCD velocity and incidence of stroke in the article. |
| 10 | Derebail VK et al,2019^55^ | USA | Retrospective study | 427 | Observational study mentions the decrease in GFR in SCD patients over time, with no mention of role of HU in the reduction of GFR. |
| 11 | Galadanci NA et al, 2015^56^ | Nigeria | Randomized placebo trial | 250 | This is a feasibility study for primary prevention of stroke in Nigerian SCD patients. The study didn’t mention our required outcomes. |
| 12 | Galadanci NA et al, 2022^57^ | UK | Cross sectional study | 1409 | The required outcome for organ dysfunction was not mentioned in the article. |
| 13 | Gulbis B et al, 2004^58^ | Belgium | Prospective study | 109 | Incidence of stroke in SCD patients not on HU is not mentioned in the study. |
| 14 | Helton KJ et al, 2014^59^ | USA | Prospective study | 161 | No mention of effect of HU on TCD velocity and incidence of stroke in the article. |
| 16 | Lagunju I et al,2018^60^ | Nigeria | Prospective observational study | 104 | This was a prospective cohort study, and the final results were published in 2021, which is included in our analysis. |
| 17 | Lagunju I et al, 2015^61^ | Nigeria | Prospective observational study | 265 | This was a prospective cohort study, and the final results were published in 2021, which is included in our analysis. |
| 18 | Lebensburger JD et al, 2018^62^ | England | Prospective study | 185 | This study doesn’t mention the effect of HU therapy on GFR and microalbuminuria. |
| 19 | Olivieri NF et al, 1998^63^ | USA, Canada | Cohort study | 17 | Pitted RBC is measured pre and post HU therapy, whereas our analysis included splenic abnormality based on liver spleen scan |
| 20 | Rankine-Mullings AE et al, 2016^64^ | Jamaica | Prospective study | 43 | There is no mention of effect of HU on TCD velocity and incidence of stroke |
| 21 | Rigano P et al, 2013^65^ | Italy | Prospective study | 104 | This study is only on cerebrovascular events in sickle cell-beta thalassemia treated with HU therapy. |
| 22 | Sabarense AP et al, 2021^66^ | Brazil | Retrospective study | 718 | There is no mention of effect of HU on TCD velocity and incidence of stroke |
| 23 | Sachdev V et al, 2017^67^ | USA | Prospective study | 57 | The required outcome for organ dysfunction was not mentioned in the article. |
| 24 | Saraf SL et al, 2019^68^ | USA | Cohort study | 270 | There is no mention of effect of HU on Kidney dysfunction in this article. |
| 25 | Zimmerman SA et al, 2004^69^ | USA | Prospective study | 13 | Exit GFR post HU therapy is not mentioned in the study |
| 26 | Smart LR et al, 2022^70^ | Tanzania | Prospective study | 202 | Prospective study with no mention of post HU effect on TCD velocity and incidence of stroke. |
| 27 | Stallworth JR et al, 2011^71^ | USA | Population based study | 2194 | The study mentions the prevalence of renal dysunction in SCD patients, but there was no mention of the effect of HU therapy on renal dysfunction. |
| 28 | Voskaridou E et al, 2004 ^72^ | Greece | Prospective study | 57 | The required outcome for organ dysfunction was not mentioned in the article. |
| 29 | Yan JH et al, 2005^73^ | USA | Non-randomized trial | 17 | The required outcome for organ dysfunction was not mentioned in the article. |
| 30 | Yu T et al, 2016^74^ | USA | Retrospective study | 174 | There is no mention of Incidence of AVN in SCD patients who are on HU therapy and not on HU therapy. |
| 31 | Zahr RS et al, 2019^75^ | USA | Prospective study | 88 | This Study doesn’t mention the prevalence of albuminuria in SCD patients who are not on HU therapy. |

**Appendix VIII: Data Quality score of the studies Included by Modified Downs and Black criteria.**

|  | **Claster S et al^1^ 1996** | **Wang WC et^2^ al 2001** | **Santos A et al^3^ 2002** | **Hankin JS et al^4^ 200** |
| --- | --- | --- | --- | --- |
| **Reporting** |  |  |  |  |
| Is the hypothesis/aim/objective of the study clearly described? | 1 | 1 | 1 | 1 |
| Are the main outcomes to be measured clearly described in the Introduction or Methods section? | 1 | 1 | 1 | 1 |
| Are the characteristics of the patients included in the study clearly described? | 1 | 1 | 1 | 1 |
| Are the interventions of interest clearly described? | 0 | 1 | 1 | 1 |
| Are the distributions of principal confounders in each group of subjects to be compared clearly described? | 0 | 0 | 0 | 0 |
| Are the main findings of the study clearly described? | 1 | 1 | 1 | 1 |
| Does the study provide estimates of the random variability in the data for the main outcomes? | 0 | 0 | 0 | 1 |
| Have all important adverse events that may be a consequence of the intervention been reported? | 1 | 1 | 1 | 1 |
| Have the characteristics of patients lost to follow-up been described? | 0 | 1 | 0 | 1 |
| Have actual probability values been reported (e.g., 0·035 rather than <0·05) for the main outcomes except where the probability value is less than 0·001? | 0 | 1 | 0 | 0 |
| **External validity** |  |  |  |  |
| Were the subjects asked to participate in the study representative of the entire population from which they were recruited? | 1 | 1 | 1 | 1 |
| Were those subjects who were prepared to participate representative of the entire population from which they were recruited? | 1 | 1 | 1 | 1 |
| Were the staff, places, and facilities where the patients were treated, representative of the treatment the majority of patients receive? | 0 | 1 | 1 | 1 |
| Was an attempt made to blind study subjects to the intervention they have received? | 0 | 1 | 0 | 1 |
| Was an attempt made to blind those measuring the main outcomes of the intervention? | 0 | 0 | 0 | 1 |
| If any of the results of the study were based on “data dredging”, was this made clear? | 0 | 0 | 0 | 0 |
| In trials and cohort studies, do the analyses adjust for different lengths of follow-up of patients, or in case-control studies, is the time period between the intervention and outcome the same for cases and controls? | 0 | 1 | 0 | 1 |
| Were the statistical tests used to assess the main outcomes appropriate? | 0 | 1 | 0 | 1 |
| Was compliance with the intervention/s reliable? | 0 |  | 0 | 1 |
| "Were the main outcome measures used | 1 | 1 | 1 | 1 |
| **Internal validity** |  |  |  |  |
| Were the patients in different intervention groups (trials and cohort studies) or were the cases and controls (case-control studies) recruited from the same population? | 0 | 0 | 1 | 1 |
| Were study subjects in different intervention groups (trials and cohort studies) or were the cases and controls (case-control studies) recruited over the same period of time? | 0 | 0 | 1 | 1 |
| Were study subjects randomised to intervention groups? | 0 | 0 | 0 | 1 |
| Was the randomized intervention assignment concealed from both patients and health care staff until recruitment was complete and irrevocable? | 0 | 0 | 0 | 1 |
| Was there adequate adjustment for confounding in the analyses from which the main findings were drawn? | 0 | 1 | 0 | 1 |
| Were losses of patients to follow-up taken into account? | 0 | 1 | 0 | 1 |
| **Power** |  |  |  |  |
| Did the study have sufficient power to detect a clinically important effect where the probability value for a difference being due to chance is less than 5%? | 0 | 0 | 0 | 0 |
| **Total** | 8 | 17 | 12 | 23 |

|  | **Tavakkoli F et al^5^ 2006** | **Kratovil T et al^6^ 2006** | **Zimmerman SA et al^7^ 2007** | **Puffer et al^8^ 2007** |
| --- | --- | --- | --- | --- |
| **Reporting** |  |  |  |  |
| Is the hypothesis/aim/objective of the study clearly described? | 1 | 1 | 1 | 1 |
| Are the main outcomes to be measured clearly described in the Introduction or Methods section? | 1 | 1 | 1 | 1 |
| Are the characteristics of the patients included in the study clearly described? | 1 | 1 | 1 | 1 |
| Are the interventions of interest clearly described? | 1 | 1 | 1 | 0 |
| Are the distributions of principal confounders in each group of subjects to be compared clearly described? | 1 | 0 | 0 | 0 |
| Are the main findings of the study clearly described? | 1 | 1 | 1 | 1 |
| Does the study provide estimates of the random variability in the data for the main outcomes? | 0 | 0 | 0 | 0 |
| Have all important adverse events that may be a consequence of the intervention been reported? | 0 | 0 | 1 | 1 |
| Have the characteristics of patients lost to follow-up been described? | 0 | 0 | 1 | 0 |
| Have actual probability values been reported (e.g., 0·035 rather than <0·05) for the main outcomes except where the probability value is less than 0·001? | 1 | 1 | 1 | 0 |
| **External validity** |  |  |  |  |
| Were the subjects asked to participate in the study representative of the entire population from which they were recruited? | 1 | 1 | 1 | 1 |
| Were those subjects who were prepared to participate representative of the entire population from which they were recruited? | 1 | 1 | 1 | 1 |
| Were the staff, places, and facilities where the patients were treated, representative of the treatment the majority of patients receive? | 1 | 1 | 1 | 1 |
| Was an attempt made to blind study subjects to the intervention they have received? | 1 | 0 | 0 | 0 |
| Was an attempt made to blind those measuring the main outcomes of the intervention? | 1 | 0 | 0 | 0 |
| If any of the results of the study were based on “data dredging”, was this made clear? | 0 | 0 | 0 | 0 |
| In trials and cohort studies, do the analyses adjust for different lengths of follow-up of patients, or in case-control studies, is the time period between the intervention and outcome the same for cases and controls? | 1 | 1 | 1 | 1 |
| Were the statistical tests used to assess the main outcomes appropriate? | 1 | 1 | 1 | 1 |
| Was compliance with the intervention/s reliable? | 0 | 0 | 1 | 0 |
| "Were the main outcome measures used | 1 | 1 | 1 | 1 |
| **Internal validity** |  |  |  |  |
| Were the patients in different intervention groups (trials and cohort studies) or were the cases and controls (case-control studies) recruited from the same population? | 1 | 1 | 1 | 1 |
| Were study subjects in different intervention groups (trials and cohort studies) or were the cases and controls (case-control studies) recruited over the same period of time? | 1 | 1 | 1 | 1 |
| Were study subjects randomised to intervention groups? | 1 | 0 | 0 | 0 |
| Was the randomized intervention assignment concealed from both patients and health care staff until recruitment was complete and irrevocable? | 1 | 0 | 0 | 0 |
| Was there adequate adjustment for confounding in the analyses from which the main findings were drawn? | 1 | 1 | 0 | 0 |
| Were losses of patients to follow-up taken into account? | 0 | 0 | 1 | 0 |
| **Power** |  |  |  |  |
| Did the study have sufficient power to detect a clinically important effect where the probability value for a difference being due to chance is less than 5%? | 1 | 0 | 4 | 0 |
| **Total** | 21 | 15 | 22 | 13 |

|  | **McKie KT et al^9^ 2007** | **Pashankar FD et al^10^ 2008** | **Hankins JS et al^11^ 2008** | **Olnes M et al^12^ 2009** |
| --- | --- | --- | --- | --- |
| **Reporting** |  |  |  |  |
| Is the hypothesis/aim/objective of the study clearly described? | 1 | 1 | 1 | 1 |
| Are the main outcomes to be measured clearly described in the Introduction or Methods section? | 1 | 1 | 1 | 1 |
| Are the characteristics of the patients included in the study clearly described? | 1 | 1 | 1 | 1 |
| Are the interventions of interest clearly described? | 1 | 0 | 0 | 1 |
| Are the distributions of principal confounders in each group of subjects to be compared clearly described? | 0 | 0 | 1 | 0 |
| Are the main findings of the study clearly described? | 1 | 1 | 1 | 1 |
| Does the study provide estimates of the random variability in the data for the main outcomes? | 0 | 0 | 0 | 0 |
| Have all important adverse events that may be a consequence of the intervention been reported? | 0 | 0 | 1 | 0 |
| Have the characteristics of patients lost to follow-up been described? | 0 | 1 | 0 | 0 |
| Have actual probability values been reported (e.g., 0·035 rather than <0·05) for the main outcomes except where the probability value is less than 0·001? | 1 | 1 | 1 | 1 |
| **External validity** |  |  |  |  |
| Were the subjects asked to participate in the study representative of the entire population from which they were recruited? | 1 | 1 | 1 | 1 |
| Were those subjects who were prepared to participate representative of the entire population from which they were recruited? | 1 | 1 | 1 | 1 |
| Were the staff, places, and facilities where the patients were treated, representative of the treatment the majority of patients receive? | 1 | 1 | 1 | 1 |
| Was an attempt made to blind study subjects to the intervention they have received? | 0 | 0 | 0 | 0 |
| Was an attempt made to blind those measuring the main outcomes of the intervention? | 0 | 0 | 0 | 0 |
| If any of the results of the study were based on “data dredging”, was this made clear? | 0 | 0 | 0 | 0 |
| In trials and cohort studies, do the analyses adjust for different lengths of follow-up of patients, or in case-control studies, is the time period between the intervention and outcome the same for cases and controls? | 1 | 1 | 0 | 0 |
| Were the statistical tests used to assess the main outcomes appropriate? | 1 | 1 | 1 | 1 |
| Was compliance with the intervention/s reliable? | 0 | 1 | 0 | 0 |
| "Were the main outcome measures used | 1 | 1 | 1 | 1 |
| **Internal validity** |  |  |  |  |
| Were the patients in different intervention groups (trials and cohort studies) or were the cases and controls (case-control studies) recruited from the same population? | 1 | 1 | 1 | 1 |
| Were study subjects in different intervention groups (trials and cohort studies) or were the cases and controls (case-control studies) recruited over the same period of time? | 1 | 1 | 1 | 1 |
| Were study subjects randomised to intervention groups? | 0 | 0 | 0 | 0 |
| Was the randomized intervention assignment concealed from both patients and health care staff until recruitment was complete and irrevocable? | 0 | 0 | 0 | 0 |
| Was there adequate adjustment for confounding in the analyses from which the main findings were drawn? | 1 | 0 | 1 | 1 |
| Were losses of patients to follow-up taken into account? | 0 | 0 | 0 | 0 |
| **Power** |  |  |  |  |
| Did the study have sufficient power to detect a clinically important effect where the probability value for a difference being due to chance is less than 5%? | 3 | 3 | 1 | 0 |
| **Total** | 18 | 18 | 16 | 14 |

|  | **Thornburg CD et al^13^ 2009** | **Grace RF et al^14^ 2010** | **Mahadeo KM et al^15^ 2011** | **Lebensburger J et al^16^ 2011** |
| --- | --- | --- | --- | --- |
| **Reporting** |  |  |  |  |
| Is the hypothesis/aim/objective of the study clearly described? | 1 | 1 | 1 | 1 |
| Are the main outcomes to be measured clearly described in the Introduction or Methods section? | 1 | 1 | 1 | 1 |
| Are the characteristics of the patients included in the study clearly described? | 1 | 1 | 1 | 1 |
| Are the interventions of interest clearly described? | 1 | 1 | 1 | 1 |
| Are the distributions of principal confounders in each group of subjects to be compared clearly described? | 0 | 1 | 1 | 1 |
| Are the main findings of the study clearly described? | 1 | 1 | 1 | 1 |
| Does the study provide estimates of the random variability in the data for the main outcomes? | 0 | 0 | 1 | 0 |
| Have all important adverse events that may be a consequence of the intervention been reported? | 1 | 0 | 0 | 0 |
| Have the characteristics of patients lost to follow-up been described? | 0 | 0 | 0 | 0 |
| Have actual probability values been reported (e.g., 0·035 rather than <0·05) for the main outcomes except where the probability value is less than 0·001? | 1 | 0 | 1 | 1 |
| **External validity** |  |  |  |  |
| Were the subjects asked to participate in the study representative of the entire population from which they were recruited? | 1 | 1 | 1 | 1 |
| Were those subjects who were prepared to participate representative of the entire population from which they were recruited? | 1 | 1 | 1 | 1 |
| Were the staff, places, and facilities where the patients were treated, representative of the treatment the majority of patients receive? | 1 | 1 | 1 | 1 |
| Was an attempt made to blind study subjects to the intervention they have received? | 0 | 0 | 0 | 0 |
| Was an attempt made to blind those measuring the main outcomes of the intervention? | 0 | 0 | 0 | 0 |
| If any of the results of the study were based on “data dredging”, was this made clear? | 0 | 0 | 0 | 0 |
| In trials and cohort studies, do the analyses adjust for different lengths of follow-up of patients, or in case-control studies, is the time period between the intervention and outcome the same for cases and controls? | 1 | 1 | 1 | 1 |
| Were the statistical tests used to assess the main outcomes appropriate? | 1 | 0 | 1 | 1 |
| Was compliance with the intervention/s reliable? | 0 | 0 | 0 | 0 |
| "Were the main outcome measures used | 1 | 1 | 1 | 1 |
| **Internal validity** |  |  |  |  |
| Were the patients in different intervention groups (trials and cohort studies) or were the cases and controls (case-control studies) recruited from the same population? | 1 | 1 | 1 | 1 |
| Were study subjects in different intervention groups (trials and cohort studies) or were the cases and controls (case-control studies) recruited over the same period of time? | 1 | 1 | 1 | 1 |
| Were study subjects randomized to intervention groups? | 0 | 0 | 0 | 0 |
| Was the randomized intervention assignment concealed from both patients and health care staff until recruitment was complete and irrevocable? | 0 | 0 | 0 | 0 |
| Was there adequate adjustment for confounding in the analyses from which the main findings were drawn? | 1 | 0 | 1 | 1 |
| Were losses of patients to follow-up taken into account? | 0 | 0 | 0 | 0 |
| **Power** |  |  |  |  |
| Did the study have sufficient power to detect a clinically important effect where the probability value for a difference being due to chance is less than 5%? | 0 | 0 | 5 | 1 |
| **Total** | 16 | 13 | 22 | 17 |

|  | **Wang WC et al^17^ 2011** | **Alvarez O et al^18^ 2012** | **Silva Junior GB et al^19^ 2012** | **Desai PC et al^20^ 2013** |
| --- | --- | --- | --- | --- |
| **Reporting** |  |  |  |  |
| Is the hypothesis/aim/objective of the study clearly described? | 1 | 1 | 1 | 1 |
| Are the main outcomes to be measured clearly described in the Introduction or Methods section? | 1 | 1 | 1 | 1 |
| Are the characteristics of the patients included in the study clearly described? | 1 | 1 | 1 | 1 |
| Are the interventions of interest clearly described? | 1 | 1 | 1 | 1 |
| Are the distributions of principal confounders in each group of subjects to be compared clearly described? | 0 | 1 | 0 | 0 |
| Are the main findings of the study clearly described? | 1 | 1 | 1 | 1 |
| Does the study provide estimates of the random variability in the data for the main outcomes? | 1 | 0 | 0 | 0 |
| Have all important adverse events that may be a consequence of the intervention been reported? | 1 | 0 | 0 | 0 |
| Have the characteristics of patients lost to follow-up been described? | 1 | 0 | 1 | 0 |
| Have actual probability values been reported (e.g., 0·035 rather than <0·05) for the main outcomes except where the probability value is less than 0·001? | 1 | 1 | 0 | 1 |
| **External validity** |  |  |  |  |
| Were the subjects asked to participate in the study representative of the entire population from which they were recruited? | 0 | 0 | 1 | 1 |
| Were those subjects who were prepared to participate representative of the entire population from which they were recruited? | 0 | 1 | 1 | 1 |
| Were the staff, places, and facilities where the patients were treated, representative of the treatment the majority of patients receive? | 1 | 1 | 1 | 1 |
| Was an attempt made to blind study subjects to the intervention they have received? | 1 | 1 | 0 | 0 |
| Was an attempt made to blind those measuring the main outcomes of the intervention? | 1 | 1 | 0 | 0 |
| If any of the results of the study were based on “data dredging”, was this made clear? | 0 | 0 | 0 | 0 |
| In trials and cohort studies, do the analyses adjust for different lengths of follow-up of patients, or in case-control studies, is the time period between the intervention and outcome the same for cases and controls? | 1 | 1 | 1 | 0 |
| Were the statistical tests used to assess the main outcomes appropriate? | 1 | 1 | 1 | 1 |
| Was compliance with the intervention/s reliable? | 0 | 0 | 0 | 0 |
| "Were the main outcome measures used | 1 | 1 | 1 | 1 |
| **Internal validity** |  |  |  |  |
| Were the patients in different intervention groups (trials and cohort studies) or were the cases and controls (case-control studies) recruited from the same population? | 1 | 1 | 1 | 1 |
| Were study subjects in different intervention groups (trials and cohort studies) or were the cases and controls (case-control studies) recruited over the same period of time? | 0 | 1 | 1 | 1 |
| Were study subjects randomized to intervention groups? | 1 | 1 | 0 | 0 |
| Was the randomized intervention assignment concealed from both patients and health care staff until recruitment was complete and irrevocable? | 1 | 1 | 0 | 0 |
| Was there adequate adjustment for confounding in the analyses from which the main findings were drawn? | 1 | 1 | 0 | 0 |
| Were losses of patients to follow-up taken into account? | 1 | 0 | 0 | 0 |
| **Power** |  |  |  |  |
| Did the study have sufficient power to detect a clinically important effect where the probability value for a difference being due to chance is less than 5%? | 4 | 5 | 1 | 3 |
| **Total** | 24 | 24 | 15 | 16 |

|  | **Aygun B et al^21^ 2013** | **Estepp JH et al^22^ 2013** | **Laurin LP et al^23^ 2014** | **Silva Junior GB et al^24^ 2014** |
| --- | --- | --- | --- | --- |
| **Reporting** |  |  |  |  |
| Is the hypothesis/aim/objective of the study clearly described? | 1 | 1 | 1 | 1 |
| Are the main outcomes to be measured clearly described in the Introduction or Methods section? | 1 | 1 | 1 | 1 |
| Are the characteristics of the patients included in the study clearly described? | 1 | 1 | 1 | 1 |
| Are the interventions of interest clearly described? | 0 | 1 | 1 | 1 |
| Are the distributions of principal confounders in each group of subjects to be compared clearly described? | 0 | 1 | 0 | 0 |
| Are the main findings of the study clearly described? | 1 | 1 | 1 | 1 |
| Does the study provide estimates of the random variability in the data for the main outcomes? | 0 | 0 | 0 | 1 |
| Have all important adverse events that may be a consequence of the intervention been reported? | 0 | 0 | 0 | 0 |
| Have the characteristics of patients lost to follow-up been described? | 0 | 0 | 0 |  |
| Have actual probability values been reported (e.g., 0·035 rather than <0·05) for the main outcomes except where the probability value is less than 0·001? | 1 | 1 | 0 | 1 |
| **External validity** |  |  |  |  |
| Were the subjects asked to participate in the study representative of the entire population from which they were recruited? | 1 | 1 | 0 | 1 |
| Were those subjects who were prepared to participate representative of the entire population from which they were recruited? | 1 | 1 | 1 | 1 |
| Were the staff, places, and facilities where the patients were treated, representative of the treatment the majority of patients receive? | 1 | 1 | 1 | 1 |
| Was an attempt made to blind study subjects to the intervention they have received? | 0 | 0 | 0 | 0 |
| Was an attempt made to blind those measuring the main outcomes of the intervention? | 0 | 0 | 0 | 0 |
| If any of the results of the study were based on “data dredging”, was this made clear? | 0 | 0 | 0 | 0 |
| In trials and cohort studies, do the analyses adjust for different lengths of follow-up of patients, or in case-control studies, is the time period between the intervention and outcome the same for cases and controls? | 0 | 0 | 0 | 0 |
| Were the statistical tests used to assess the main outcomes appropriate? | 1 | 1 | 1 | 1 |
| Was compliance with the intervention/s reliable? | 0 | 0 | 1 | 0 |
| "Were the main outcome measures used | 0 | 1 | 1 | 1 |
| **Internal validity** |  |  |  |  |
| Were the patients in different intervention groups (trials and cohort studies) or were the cases and controls (case-control studies) recruited from the same population? | 1 | 1 | 1 | 1 |
| Were study subjects in different intervention groups (trials and cohort studies) or were the cases and controls (case-control studies) recruited over the same period of time? | 1 | 1 | 1 | 1 |
| Were study subjects randomized to intervention groups? | 0 | 0 | 0 | 0 |
| Was the randomized intervention assignment concealed from both patients and health care staff until recruitment was complete and irrevocable? | 0 | 0 | 0 | 0 |
| Was there adequate adjustment for confounding in the analyses from which the main findings were drawn? | 0 | 0 | 0 | 1 |
| Were losses of patients to follow-up taken into account? | 0 | 0 | 0 | 0 |
| **Power** |  |  |  |  |
| Did the study have sufficient power to detect a clinically important effect where the probability value for a difference being due to chance is less than 5%? | 0 | 2 | 2 | 0 |
| **Total** | 11 | 16 | 14 | 15 |

|  | **Hankins JS et al^25^ 2014** | **Nottage KA et al^26^ 2014** | **Hankins JS et al^27^ 2015** | **Bartolucci P et al^28^ 2016** |
| --- | --- | --- | --- | --- |
| **Reporting** |  |  |  |  |
| Is the hypothesis/aim/objective of the study clearly described? | 1 | 1 | 1 | 1 |
| Are the main outcomes to be measured clearly described in the Introduction or Methods section? | 1 | 1 | 1 | 1 |
| Are the characteristics of the patients included in the study clearly described? | 1 | 1 | 1 | 1 |
| Are the interventions of interest clearly described? | 1 | 1 | 1 | 0 |
| Are the distributions of principal confounders in each group of subjects to be compared clearly described? | 1 | 0 | 0 | 0 |
| Are the main findings of the study clearly described? | 1 | 1 | 1 | 1 |
| Does the study provide estimates of the random variability in the data for the main outcomes? | 0 | 0 | 1 | 1 |
| Have all important adverse events that may be a consequence of the intervention been reported? | 1 | 0 | 1 | 0 |
| Have the characteristics of patients lost to follow-up been described? | 1 | 1 | 0 | 0 |
| Have actual probability values been reported (e.g., 0·035 rather than <0·05) for the main outcomes except where the probability value is less than 0·001? | 0 | 1 | 0 | 0 |
| **External validity** |  |  |  |  |
| Were the subjects asked to participate in the study representative of the entire population from which they were recruited? | 1 | 1 | 0 | 0 |
| Were those subjects who were prepared to participate representative of the entire population from which they were recruited? | 1 | 1 | 1 | 0 |
| Were the staff, places, and facilities where the patients were treated, representative of the treatment the majority of patients receive? | 1 | 1 | 1 | 0 |
| Was an attempt made to blind study subjects to the intervention they have received? | 0 | 0 | 0 | 0 |
| Was an attempt made to blind those measuring the main outcomes of the intervention? | 0 | 0 | 0 | 0 |
| If any of the results of the study were based on “data dredging”, was this made clear? | 0 | 0 | 0 | 0 |
| In trials and cohort studies, do the analyses adjust for different lengths of follow-up of patients, or in case-control studies, is the time period between the intervention and outcome the same for cases and controls? | 1 | 1 | 1 | 0 |
| Were the statistical tests used to assess the main outcomes appropriate? | 1 | 1 | 1 | 1 |
| Was compliance with the intervention/s reliable? | 0 | 0 | 0 | 0 |
| "Were the main outcome measures used | 1 | 1 | 1 | 1 |
| **Internal validity** |  |  |  |  |
| Were the patients in different intervention groups (trials and cohort studies) or were the cases and controls (case-control studies) recruited from the same population? | 1 | 1 | 0 | 1 |
| Were study subjects in different intervention groups (trials and cohort studies) or were the cases and controls (case-control studies) recruited over the same period of time? | 1 | 1 | 0 | 1 |
| Were study subjects randomized to intervention groups? | 1 | 0 | 1 | 0 |
| Was the randomized intervention assignment concealed from both patients and health care staff until recruitment was complete and irrevocable? | 0 | 0 | 1 | 0 |
| Was there adequate adjustment for confounding in the analyses from which the main findings were drawn? | 0 | 1 | 0 | 1 |
| Were losses of patients to follow-up taken into account? | 1 | 1 | 0 | 1 |
| **Power** |  |  |  |  |
| Did the study have sufficient power to detect a clinically important effect where the probability value for a difference being due to chance is less than 5%? | 0 | 1 | 0 | 3 |
| **Total** | 18 | 18 | 14 | 14 |

|  | **Ware RE et al^29^ 2016** | **Nottage KA et al^30^ 2016** | **Tehseen S et al^31^ 2017** | **Adegoke SA et al^32^ 2017** |
| --- | --- | --- | --- | --- |
| **Reporting** |  |  |  |  |
| Is the hypothesis/aim/objective of the study clearly described? | 1 | 1 | 1 | 1 |
| Are the main outcomes to be measured clearly described in the Introduction or Methods section? | 1 | 1 | 1 | 1 |
| Are the characteristics of the patients included in the study clearly described? | 1 | 1 | 1 | 1 |
| Are the interventions of interest clearly described? | 1 | 0 | 1 | 1 |
| Are the distributions of principal confounders in each group of subjects to be compared clearly described? | 0 | 0 | 0 | 1 |
| Are the main findings of the study clearly described? | 1 | 1 | 1 | 1 |
| Does the study provide estimates of the random variability in the data for the main outcomes? | 0 | 1 | 1 | 1 |
| Have all important adverse events that may be a consequence of the intervention been reported? | 1 | 0 | 0 | 0 |
| Have the characteristics of patients lost to follow-up been described? | 1 | 0 | 0 | 1 |
| Have actual probability values been reported (e.g., 0·035 rather than <0·05) for the main outcomes except where the probability value is less than 0·001? | 1 | 0 | 1 | 1 |
| **External validity** |  |  |  |  |
| Were the subjects asked to participate in the study representative of the entire population from which they were recruited? | 1 | 0 | 0 | 1 |
| Were those subjects who were prepared to participate representative of the entire population from which they were recruited? | 1 | 0 | 0 | 1 |
| Were the staff, places, and facilities where the patients were treated, representative of the treatment the majority of patients receive? | 1 | 0 | 1 | 1 |
| Was an attempt made to blind study subjects to the intervention they have received? | 1 | 0 | 0 | 0 |
| Was an attempt made to blind those measuring the main outcomes of the intervention? | 1 | 0 | 0 | 0 |
| If any of the results of the study were based on “data dredging”, was this made clear? | 0 | 0 | 0 | 0 |
| In trials and cohort studies, do the analyses adjust for different lengths of follow-up of patients, or in case-control studies, is the time period between the intervention and outcome the same for cases and controls? | 1 | 0 | 1 | 1 |
| Were the statistical tests used to assess the main outcomes appropriate? | 1 | 1 | 1 | 1 |
| Was compliance with the intervention/s reliable? | 1 | 0 | 0 | 1 |
| "Were the main outcome measures used | 1 | 1 | 1 | 1 |
| **Internal validity** |  |  |  |  |
| Were the patients in different intervention groups (trials and cohort studies) or were the cases and controls (case-control studies) recruited from the same population? | 1 | 1 | 1 | 1 |
| Were study subjects in different intervention groups (trials and cohort studies) or were the cases and controls (case-control studies) recruited over the same period of time? | 1 | 1 | 1 | 1 |
| Were study subjects randomized to intervention groups? | 1 | 0 | 0 | 1 |
| Was the randomized intervention assignment concealed from both patients and health care staff until recruitment was complete and irrevocable? | 1 | 0 | 0 | 1 |
| Was there adequate adjustment for confounding in the analyses from which the main findings were drawn? | 1 | 1 | 0 | 0 |
| Were losses of patients to follow-up taken into account? | 1 | 1 | 0 | 1 |
| **Power** |  |  |  |  |
| Did the study have sufficient power to detect a clinically important effect where the probability value for a difference being due to chance is less than 5%? | 3 | 2 | 4 | 2 |
| **Total** | 26 | 13 | 17 | 23 |

|  | **Ghafuri DL et al^33^ 2017** | **Adekile AD et al^34^ 2019** | **Yates AM et al^35^ 2019** | **Garadah T et al^36^ 2019** |
| --- | --- | --- | --- | --- |
| **Reporting** |  |  |  |  |
| Is the hypothesis/aim/objective of the study clearly described? | 1 | 1 | 1 | 1 |
| Are the main outcomes to be measured clearly described in the Introduction or Methods section? | 1 | 1 | 1 | 1 |
| Are the characteristics of the patients included in the study clearly described? | 1 | 1 | 1 | 1 |
| Are the interventions of interest clearly described? | 1 | 1 | 1 | 1 |
| Are the distributions of principal confounders in each group of subjects to be compared clearly described? | 0 | 0 | 1 | 0 |
| Are the main findings of the study clearly described? | 1 | 1 |  | 1 |
| Does the study provide estimates of the random variability in the data for the main outcomes? | 0 | 0 | 0 | 0 |
| Have all important adverse events that may be a consequence of the intervention been reported? | 0 | 1 | 1 | 0 |
| Have the characteristics of patients lost to follow-up been described? | 0 | 1 | 1 | 1 |
| Have actual probability values been reported (e.g., 0·035 rather than <0·05) for the main outcomes except where the probability value is less than 0·001? | 0 | 1 | 1 | 1 |
| **External validity** |  |  |  |  |
| Were the subjects asked to participate in the study representative of the entire population from which they were recruited? | 1 | 1 | 1 | 1 |
| Were those subjects who were prepared to participate representative of the entire population from which they were recruited? | 1 | 1 | 1 | 1 |
| Were the staff, places, and facilities where the patients were treated, representative of the treatment the majority of patients receive? | 1 | 1 | 1 | 1 |
| Was an attempt made to blind study subjects to the intervention they have received? | 0 | 0 | 1 | 0 |
| Was an attempt made to blind those measuring the main outcomes of the intervention? | 0 | 0 | 1 | 0 |
| If any of the results of the study were based on “data dredging”, was this made clear? | 0 | 0 | 0 | 1 |
| In trials and cohort studies, do the analyses adjust for different lengths of follow-up of patients, or in case-control studies, is the time period between the intervention and outcome the same for cases and controls? | 1 | 1 | 1 | 1 |
| Were the statistical tests used to assess the main outcomes appropriate? | 1 | 1 | 1 | 1 |
| Was compliance with the intervention/s reliable? | 0 | 1 | 1 | 1 |
| "Were the main outcome measures used | 1 | 1 | 1 | 1 |
| **Internal validity** |  |  |  |  |
| Were the patients in different intervention groups (trials and cohort studies) or were the cases and controls (case-control studies) recruited from the same population? | 1 | 1 | 1 | 1 |
| Were study subjects in different intervention groups (trials and cohort studies) or were the cases and controls (case-control studies) recruited over the same period of time? | 1 | 1 | 1 | 1 |
| Were study subjects randomized to intervention groups? | 0 | 0 | 0 | 1 |
| Was the randomized intervention assignment concealed from both patients and health care staff until recruitment was complete and irrevocable? | 0 | 0 | 0 | 0 |
| Was there adequate adjustment for confounding in the analyses from which the main findings were drawn? | 0 | 0 | 1 | 0 |
| Were losses of patients to follow-up taken into account? | 0 | 1 | 1 | 0 |
| **Power** |  |  |  |  |
| Did the study have sufficient power to detect a clinically important effect where the probability value for a difference being due to chance is less than 5%? | 5 | 1 | 5 | 2 |
| **Total** | 18 | 19 | 26 | 20 |

|  | **Kapustin D et al^37^ 2019** | **Opoka RO et al^38^ 2020** | **Lagunju lA et al^39^ 2021** | **Rai P et al^40^ 2021** |
| --- | --- | --- | --- | --- |
| **Reporting** |  |  |  |  |
| Is the hypothesis/aim/objective of the study clearly described? | 1 | 1 | 1 | 1 |
| Are the main outcomes to be measured clearly described in the Introduction or Methods section? | 1 | 1 | 1 | 1 |
| Are the characteristics of the patients included in the study clearly described? | 1 | 1 | 1 | 1 |
| Are the interventions of interest clearly described? | 1 | 1 | 1 | 1 |
| Are the distributions of principal confounders in each group of subjects to be compared clearly described? | 0 | 1 | 1 | 1 |
| Are the main findings of the study clearly described? | 1 | 1 | 1 | 1 |
| Does the study provide estimates of the random variability in the data for the main outcomes? | 1 | 0 | 0 | 0 |
| Have all important adverse events that may be a consequence of the intervention been reported? | 0 | 1 | 1 | 1 |
| Have the characteristics of patients lost to follow-up been described? | 0 | 0 | 1 | 1 |
| Have actual probability values been reported (e.g., 0·035 rather than <0·05) for the main outcomes except where the probability value is less than 0·001? | 0 | 1 | 0 | 1 |
| **External validity** |  |  |  |  |
| Were the subjects asked to participate in the study representative of the entire population from which they were recruited? | 1 | 1 | 1 | 1 |
| Were those subjects who were prepared to participate representative of the entire population from which they were recruited? | 1 | 1 | 1 | 1 |
| Were the staff, places, and facilities where the patients were treated, representative of the treatment the majority of patients receive? | 1 | 1 | 1 | 1 |
| Was an attempt made to blind study subjects to the intervention they have received? | 1 | 1 | 1 | 1 |
| Was an attempt made to blind those measuring the main outcomes of the intervention? | 0 | 1 | 1 | 1 |
| If any of the results of the study were based on “data dredging”, was this made clear? | 0 | 0 | 0 | 0 |
| In trials and cohort studies, do the analyses adjust for different lengths of follow-up of patients, or in case-control studies, is the time period between the intervention and outcome the same for cases and controls? | 1 | 1 | 1 | 1 |
| Were the statistical tests used to assess the main outcomes appropriate? | 1 | 1 | 1 | 1 |
| Was compliance with the intervention/s reliable? | 0 | 1 | 1 | 1 |
| "Were the main outcome measures used | 1 | 1 | 1 | 1 |
| **Internal validity** |  |  |  |  |
| Were the patients in different intervention groups (trials and cohort studies) or were the cases and controls (case-control studies) recruited from the same population? | 1 | 1 | 1 | 1 |
| Were study subjects in different intervention groups (trials and cohort studies) or were the cases and controls (case-control studies) recruited over the same period of time? | 1 | 1 | 1 | 1 |
| Were study subjects randomized to intervention groups? | 0 | 1 | 0 | 0 |
| Was the randomized intervention assignment concealed from both patients and health care staff until recruitment was complete and irrevocable? | 0 | 1 | 0 | 0 |
| Was there adequate adjustment for confounding in the analyses from which the main findings were drawn? | 0 | 1 | 1 | 1 |
| Were losses of patients to follow-up taken into account? | 0 | 0 | 1 | 1 |
| **Power** |  |  |  |  |
| Did the study have sufficient power to detect a clinically important effect where the probability value for a difference being due to chance is less than 5%? | 1 | 5 | 4 | 4 |
| **Total** | 16 | 27 | 26 | 26 |

|  | **Rankine-Mullings A et al^41^ 2021** | **Wang WC et al^42^ 2021** | **Estepp JH et al^43^ 2021** | **Karkoska K et al^44^ 2021** | **Peine BR et al^45^ 2022** |
| --- | --- | --- | --- | --- | --- |
| **Reporting** |  |  |  |  |  |
| Is the hypothesis/aim/objective of the study clearly described? | 1 | 1 | 1 | 1 | 1 |
| Are the main outcomes to be measured clearly described in the Introduction or Methods section? | 1 | 1 | 1 | 1 | 1 |
| Are the characteristics of the patients included in the study clearly described? | 1 | 1 | 1 | 1 | 1 |
| Are the interventions of interest clearly described? | 1 | 1 | 1 | 0 | 1 |
| Are the distributions of principal confounders in each group of subjects to be compared clearly described? | 1 | 1 | 1 | 0 | 1 |
| Are the main findings of the study clearly described? | 1 | 1 | 1 | 1 | 1 |
| Does the study provide estimates of the random variability in the data for the main outcomes? | 0 | 0 | 0 | 0 | 1 |
| Have all important adverse events that may be a consequence of the intervention been reported? | 1 | 0 | 0 | 0 | 1 |
| Have the characteristics of patients lost to follow-up been described? | 1 | 1 | 1 | 1 | 0 |
| Have actual probability values been reported (e.g., 0·035 rather than <0·05) for the main outcomes except where the probability value is less than 0·001? | 1 | 1 | 1 | 0 | 0 |
| **External validity** |  |  |  |  |  |
| Were the subjects asked to participate in the study representative of the entire population from which they were recruited? | 1 | 1 | 1 | 1 | 1 |
| Were those subjects who were prepared to participate representative of the entire population from which they were recruited? | 1 | 1 | 1 | 1 | 1 |
| Were the staff, places, and facilities where the patients were treated, representative of the treatment the majority of patients receive? | 1 | 1 | 0 | 1 | 1 |
| Was an attempt made to blind study subjects to the intervention they have received? | 1 | 0 | 0 | 0 | 0 |
| Was an attempt made to blind those measuring the main outcomes of the intervention? | 1 | 0 | 0 | 0 | 0 |
| If any of the results of the study were based on “data dredging”, was this made clear? | 0 | 0 | 0 | 0 | 0 |
| In trials and cohort studies, do the analyses adjust for different lengths of follow-up of patients, or in case-control studies, is the time period between the intervention and outcome the same for cases and controls? | 1 | 1 | 1 | 1 | 1 |
| Were the statistical tests used to assess the main outcomes appropriate? | 1 | 1 | 1 | 1 | 1 |
| Was compliance with the intervention/s reliable? | 1 | 0 | 1 | 0 | 1 |
| "Were the main outcome measures used | 1 | 1 | 1 | 1 | 1 |
| **Internal validity** |  |  |  |  |  |
| Were the patients in different intervention groups (trials and cohort studies) or were the cases and controls (case-control studies) recruited from the same population? | 1 | 1 | 1 | 1 | 1 |
| Were study subjects in different intervention groups (trials and cohort studies) or were the cases and controls (case-control studies) recruited over the same period of time? | 1 | 1 | 1 | 1 | 1 |
| Were study subjects randomized to intervention groups? | 0 | 0 | 0 | 0 | 0 |
| Was the randomized intervention assignment concealed from both patients and health care staff until recruitment was complete and irrevocable? | 0 | 0 | 0 | 0 | 0 |
| Was there adequate adjustment for confounding in the analyses from which the main findings were drawn? | 0 | 1 | 1 | 0 | 1 |
| Were losses of patients to follow-up taken into account? | 1 | 1 | 1 | 0 | 1 |
| **Power** |  |  |  |  |  |
| Did the study have sufficient power to detect a clinically important effect where the probability value for a difference being due to chance is less than 5%? | 0 | 0 | 0 | 1 | 6 |
| **Total** | 21 | 18 | 18 | 14 | 25 |

**Appendix IX: PRISMA2020Checklist**

| **Section and Topic** | **Item#** | **Checklist item** | **Location where item**  **Is reported** |
| --- | --- | --- | --- |
| **TITLE** | | |  |
| Title | 1 | Identify the report as a systematic review. | Page no 1, Line no 1 |
| **ABSTRACT** | | |  |
| Abstract | 2 | See the PRISMA 2020 for Abstracts checklist. | Page no 4, Line no 42 |
| **INTRODUCTION** | | |  |
| Rationale | 3 | Describe the rationale for the review in the context of existing knowledge. | Page no 7, Line no 121 |
| Objectives | 4 | Provide an explicit statement of the objective(s) or question(s) the review addresses. | Page no 7, Line no 123 |
| **METHODS** | | |  |
| Eligibility criteria | 5 | Specify the inclusion and exclusion criteria for the review and how studies were grouped for the syntheses. | Page no 8, Line no 132 |
| Information sources | 6 | Specify all databases, registers, websites, organisations, reference lists and other sources searched or consulted to identify studies. Specify the date when each source was last searched or consulted. | Page no 8, Line no 129 |
| Search strategy | 7 | Present the full search strategies for all databases, registers and websites, including any filters and limits used. | Supplementary File, Appendix I, Page no 2-3 |
| Selection process | 8 | Specify the methods used to decide whether a study met the inclusion criteria of the review, including how many reviewers screened each record and each report retrieved, whether they worked independently, and if applicable, details of automation tools used in the process. | Page no 8, Line no 140 |
| Data collection process | 9 | Specify the methods used to collect data from reports, including how many reviewers collected data from each report, whether they worked independently, any processes for obtaining or confirming data from study investigators, and if applicable, details of automation tools used in the process. | Page no 8, Line no 145 |
| Data items | 10a | List and define all outcomes for which data were sought. Specify whether all results that were compatible with each outcome domain in each study were sought(e.g. for all measures, timepoints, analyses),and if not ,the methods used to decide which results to collect. | Page no 8, Line no 146 |
|  | 10b | List and define all other variables for which data were sought(e.g. participant and intervention characteristics, funding sources).Describe any assumptions made about any missing or unclear information. | Page no 8, Line no 146 |
| Study risk of bias assessment | 11 | Specify the methods used to assess risk of bias in the included studies, including details- of the tool(s) used, how many reviewers assessed each study and whether they worked independently, and if applicable, details of automation tools used in the process. | Page no 8, Line no 140 |
| Effect measures | 12 | Specify for each outcome the effect measure(s)(e.g.risk ratio,mean difference) used in the synthesis or presentation of results. | Page no 9, Line no 153 |
| Synthesis methods | 13a | Describe the processes used to decide which studies were eligible for each synthesis (e.g. tabulating the study intervention characteristics and comparing against the planned groups for each synthesis(item#5)). | Page no 8, Line no 132 |
|  | 13b | Describe any methods required to prepare the data for presentation or synthesis, such as handling of missing summary statistics, or data conversions. | NA |
|  | 13c | Describe any methods used to tabulate or visually display results of individual studies and syntheses. | Page no 8, Line no 151 |
|  | 13d | Describe any methods used to synthesize results and provide a rationale for the choice(s).If meta-analysis was performed,describe the model(s),method(s) to identify the presence and extent of statistical heterogeneity ,and software package(s)used. | Page no 8, Line no 150 |
|  | 13e | Describeanymethodsusedtoexplorepossiblecausesofheterogeneityamongstudyresults(e.g.subgroupanalysis,meta-regression). | Page no 9, Line no 155 |
|  | 13f | Describe any sensitivity analyses conducted to assess robustness of the synthesized results. | Supplementary File, Appendix IV, Page no 9-12 |
| Reporting bias assessment | 14 | Describe any methods used to assess risk of bias due to missing results in a synthesis(arising from reporting biases). | NA |
| Certainty assessment | 15 | Describe any methods used to assess certainty (or confidence) in the body of evidence for an outcome. | Page no 9, Line no 155 |

| **Section andTopic** | **Item#** | **Checklistitem** | **Locationwhereitem**  **isreported** |
| --- | --- | --- | --- |
| **RESULTS** | | |  |
| Study selection | 16a | Describe the results of the search and selection process,from the number of records identified in the search to the number of studies included in the review, ideally using a flow diagram. | Figure 1  Page no 10, Line no 167 |
|  | 16b | Cite studies that might appear to meet the inclusion criteria, but which were excluded, and explain why they were excluded. | Supplementary File, Appendix VII, Page no 24-33 |
| Study characteristics | 17 | Cite each included study and present its characteristics. | Supplementary File, Appendix II, Page no 4-5 |
| Risk of bias in studies | 18 | Present assessments of risk of bias for each included study. | Supplementary File, Appendix IV, Page no 9-12 |
| Results of individual studies | 19 | For all outcomes, present, for each study: (a) summary statistics for each group (where appropriate) and (b) an effect estimate and its precision (e.g. confidence/credible interval), ideally using structured tables or plots. | Figure 2(a-d)  Page 10, Line 173; Page no 11 Line 193, 197; Page no 12, Line no 224 |
| Results of syntheses | 20a | For each synthesis, briefly summarise the characteristics and risk of bias among contributing studies. | Figure 2(a-d)  Page no 10, Line 173; Page no 11 Line no 193, 197; Page no 12, Line no 224 |
|  | 20b | Present results of all statistical syntheses conducted. If meta-analysis was done, present for each the summary estimate and its precision (e.g.confidence/credible interval) and measures of statistical heterogeneity.If comparing groups, describe the direction of the effect. | Figure 2(a-d)  Page no 10, Line 173; Page no 11 Line no 193, 197; Page no 12, Line no 224 |
|  | 20c | Present results of all investigations of possible causes of heterogeneity among study results. | Supplementary File, Appendix V, Line no 13-21 |
|  | 20d | Present results of all sensitivity analyses conducted to assess the robustness of the synthesized results. | Supplementary File, Appendix IV, Page no 9-12 |
| Reporting biases | 21 | Present assessments of risk of bias due to missing results(arising from reporting biases) for each synthesis assessed. | NA |
| Certainty of evidence | 22 | Present assessments of certainty(or confidence) in the body of evidence for each outcome assessed. | Supplementary File, Appendix IV, Page no 9-12 |
| **DISCUSSION** | | |  |
| Discussion | 23a | Provide a general interpretation of the results in the context of other evidence. | Page no 14, Line no 247 |
|  | 23b | Discuss any limitations of the evidence included in the review. | Page no 17, Line no 321 |
|  | 23c | Discuss any limitations of the review processes used. | Page no 17, Line no 321 |
|  | 23d | Discuss implications of the results for practice, policy, and future research. | Page no 17, Line no 328 |
| **OTHERINFORMATION** | | |  |
| Registration and protocol | 24a | Provide registration information for the review,including register name and registration number,or state that there view was not registered. | Page no 9, Line no 158 |
|  | 24b | Indicate where the review protocol can be accessed,or state that a protocol was not prepared. | Page no 9, Line no 159 |
|  | 24c | Describe and explain any amendments to information provided at registration or in the protocol. | NA |
| Support | 25 | Describe sources of financial or non-financial support for the review,and the role of the funders or sponsors in the review. | Page no 9, Line no 163 |
| Competing interests | 26 | Declare any competing interests of review authors. | Page no 18, Line no 348 |
| Availability of data, code and other materials | 27 | Report which of the following are publicly available and where they can be found: template data collection forms; data extracted from included studies; data used for all analyses; analytic code; any other materials used in the review. | Page no 18, Line no 351 |

**References:**

- 1. Claster S, Vichinsky E. First report of reversal of organ dysfunction in sickle cell anemia by the use of hydroxyurea: splenic regeneration. *Blood* 1996; **88**: 1951–3.
  2. Wang WC, Wynn LW, Rogers ZR, Scott JP, Lane PA, Ware RE. A two-year pilot trial of hydroxyurea in very young children with sickle-cell anemia. *J Pediatr* 2001; **139**: 790–6.
  3. Santos A, Pinheiro V, Anjos C, *et al.*Scintigraphic follow-up of the effects of therapy with hydroxyurea on splenic function in patients with sickle cell disease. *Eur J Nucl Med Mol Imaging* 2002; **29**: 536–41.
  4. Hankins JS, Ware RE, Rogers ZR, *et al.* Long-term hydroxyurea therapy for infants with sickle cell anemia: the HUSOFT extension study. *Blood* 2005; **106**: 2269–75.
  5. Tavakkoli F, Nahavandi M, Wyche MQ, Castro O. Effects of hydroxyurea treatment on cerebral oxygenation in adult patients with sickle cell disease: an open-label pilot study. *Clinical Therapeutics* 2005; **27**: 1083–8.
  6. Kratovil T, Bulas D, Driscoll MC, Speller-Brown B, McCarter R, Minniti CP. Hydroxyurea therapy lowers TCD velocities in children with sickle cell disease. *Pediatr Blood Cancer* 2006; **47**: 894–900.
  7. Zimmerman SA, Schultz WH, Burgett S, Mortier NA, Ware RE. Hydroxyurea therapy lowers transcranial Doppler flow velocities in children with sickle cell anemia. *Blood* 2007; **110**: 1043–7.
  8. Puffer E, Schatz J, Roberts CW. The association of oral hydroxyurea therapy with improved cognitive functioning in sickle cell disease. Child Neuropsychol. 2007 Mar;13(2):142-54.
  9. McKie KT, Hanevold CD, Hernandez C, Waller JL, Ortiz L, McKie KM. Prevalence, prevention, and treatment of microalbuminuria and proteinuria in children with sickle cell disease. *J PediatrHematol Oncol* 2007; **29**: 140–4.
  10. Pashankar FD, Carbonella J, Bazzy-Asaad A, Friedman A. Longitudinal follow up of elevated pulmonary artery pressures in children with sickle cell disease. *Br J Haematol* 2009; **144**: 736–41.
  11. Hankins JS, Helton KJ, McCarville MB, Li C-S, Wang WC, Ware RE. Preservation of spleen and brain function in children with sickle cell anemia treated with hydroxyurea. *Pediatr Blood Cancer* 2008; **50**: 293–7.
  12. Olnes M, Chi A, Haney C, *et al.* Improvement in hemolysis and pulmonary arterial systolic pressure in adult patients with sickle cell disease during treatment with hydroxyurea. *Am J Hematol* 2009; **84**: 530–2.
  13. Grace RF, Su H, Sena L, Poussaint TY, Heeney MM, Gutierrez A. Resolution of cerebral artery stenosis in a child with sickle cell anemia treated with hydroxyurea. Am J Hematol. 2010 Feb;85(2):135-7
  14. Thornburg CD, Dixon N, Burgett S, *et al.* A pilot study of hydroxyurea to prevent chronic organ damage in young children with sickle cell anemia. *Pediatr Blood Cancer* 2009; **52**: 609–15.
  15. Mahadeo KM, Oyeku S, Taragin B, *et al.* Increased prevalence of osteonecrosis of the femoral head in children and adolescents with sickle-cell disease. *Am J Hematol* 2011; **86**: 806–8.
  16. Lebensburger J, Johnson SM, Askenazi DJ, Rozario NL, Howard TH, Hilliard LM. Protective role of hemoglobin and fetalhemoglobin in early kidney disease for children with sickle cell anemia. *Am J Hematol* 2011; **86**: 430–2.
  17. Wang WC, Ware RE, Miller ST, *et al.* Hydroxycarbamide in very young children with sickle-cell anaemia: a multicentre, randomised, controlled trial (BABY HUG). *Lancet* 2011; **377**: 1663–72.
  18. Alvarez O, Miller ST, Wang WC, *et al.* Effect of hydroxyurea treatment on renal function parameters: results from the multi-center placebo-controlled BABY HUG clinical trial for infants with sickle cell anemia. *Pediatr Blood Cancer* 2012; **59**: 668–74.
  19. Silva Junior GB, Libório AB, Vieira APF, *et al.* Evaluation of renal function in sickle cell disease patients in Brazil. *Braz J Med Biol Res* 2012; **45**: 652–5.
  20. Desai PC, May RC, Jones SK, *et al.* Longitudinal study of echocardiography-derived tricuspid regurgitant jet velocity in sickle cell disease. *Br J Haematol* 2013; **162**: 836–41.
  21. Aygun B, Mortier NA, Smeltzer MP, Shulkin BL, Hankins JS, Ware RE. Hydroxyurea treatment decreases glomerular hyperfiltration in children with sickle cell anemia. Am J Hematol. 2013 Feb;88(2):116-9.
  22. Estepp JH, Smeltzer MP, Wang WC, Hoehn ME, Hankins JS, Aygun B. Protection from sickle cell retinopathy is associated with elevated HbF levels and hydroxycarbamide use in children. *Br J Haematol* 2013; **161**: 402–5.
  23. Laurin L-P, Nachman PH, Desai PC, Ataga KI, Derebail VK. Hydroxyurea is associated with lower prevalence of albuminuria in adults with sickle cell disease. *Nephrol Dial Transplant* 2014; **29**: 1211–8.
  24. Silva Junior GB, Vieira APF, Couto Bem AX, *et al.* Proteinuria in adults with sickle-cell disease: the role of hydroxycarbamide(hydroxyurea) as a protective agent. *Int J Clin Pharm* 2014; **36**: 766–70.
  25. Hankins JS, Aygun B, Nottage K, *et al.* From infancy to adolescence: fifteen years of continuous treatment with hydroxyurea in sickle cell anemia. *Medicine (Baltimore)* 2014; **93**: e215.
  26. Nottage KA, Ware RE, Winter B, *et al.* Predictors of splenic function preservation in children with sickle cell anemia treated with hydroxyurea. *Eur J Haematol* 2014; **93**: 377–83.
  27. Hankins JS, McCarville MB, Rankine-Mullings A, *et al.* Prevention of conversion to abnormal transcranial Doppler with hydroxyurea in sickle cell anemia: A Phase III international randomized clinical trial. *Am J Hematol* 2015; **90**: 1099–105.
  28. Bartolucci P, Habibi A, Stehlé T, *et al.* Six Months of Hydroxyurea Reduces Albuminuria in Patients with Sickle Cell Disease. *J Am Soc Nephrol* 2016; **27**: 1847–53.
  29. Ware RE, Davis BR, Schultz WH, *et al.* Hydroxycarbamide versus chronic transfusion for maintenance of transcranial doppler flow velocities in children with sickle cell anaemia-TCD With Transfusions Changing to Hydroxyurea (TWiTCH): a multicentre, open-label, phase 3, non-inferiority trial. *Lancet* 2016; **387**: 661–70.
  30. Nottage KA, Ware RE, Aygun B, *et al.* Hydroxycarbamide treatment and brain MRI/MRA findings in children with sickle cell anaemia. *Br J Haematol* 2016; **175**: 331–8.
  31. Tehseen S, Joiner CH, Lane PA, Yee ME. Changes in urine albumin to creatinine ratio with the initiation of hydroxyurea therapy among children and adolescents with sickle cell disease. *Pediatr Blood Cancer* 2017; **64**. DOI:10.1002/pbc.26665.
  32. Adegoke SA, Macedo-Campos R de S, Braga JAP, Figueiredo MS, Silva GS. Changes in Transcranial Doppler Flow Velocities in Children with Sickle Cell Disease: The Impact of Hydroxyurea Therapy. *J Stroke Cerebrovasc Dis* 2018; **27**: 425–31.
  33. Ghafuri DL, Chaturvedi S, Rodeghier M, *et al.* Secondary benefit of maintaining normal transcranial Doppler velocities when using hydroxyurea for prevention of severe sickle cell anemia. *Pediatr Blood Cancer* 2017; **64**.
  34. Adekile AD, Gupta R, Al-Khayat A, Mohammed A, Atyani S, Thomas D. Risk of avascular necrosis of the femoral head in children with sickle cell disease on hydroxyurea: MRI evaluation. *Pediatr Blood Cancer* 2019; **66**: e27503.
  35. Yates AM, Joshi VM, Aygun B, *et al.* Elevated tricuspid regurgitation velocity in congenital hemolyticanemias: Prevalence and laboratory correlates. *Pediatr Blood Cancer* 2019; **66**: e27717.
  36. Garadah T, Mandeel F, Jaradat A, Bin Thani K. The Effects of Hydroxyurea Therapy on the Six-Minute Walk Distance in Patients with Adult Sickle Cell Anemia: An Echocardiographic Study. *J Blood Med* 2019; **10**: 443–52.
  37. Kapustin D, Leung J, Odame I, Williams S, Shroff M, Kassner A. Hydroxycarbamide treatment in children with Sickle Cell Anaemia is associated with more intact white matter integrity: a quantitative MRI study. *Br J Haematol* 2019; **187**: 238–45.
  38. Opoka RO, Hume HA, Latham TS, *et al.* Hydroxyurea to lower transcranial Doppler velocities and prevent primary stroke: the Uganda NOHARM sickle cell anemia cohort. *Haematologica* 2020; **105**: e272–5.
  39. Lagunju IA, Labaeka A, Ibeh JN, Orimadegun AE, Brown BJ, Sodeinde OO. Transcranial Doppler screening in Nigerian children with sickle cell disease: A 10-year longitudinal study on the SPPIBA cohort. *Pediatr Blood Cancer* 2021; **68**: e28906.
  40. Rai P, Joshi VM, Goldberg JF, *et al.* Longitudinal effect of disease-modifying therapy on tricuspid regurgitant velocity in children with sickle cell anemia. *Blood Adv* 2021; **5**: 89–98.
  41. Rankine-Mullings A, Reid M, Soares D, *et al.* Hydroxycarbamide treatment reduces transcranial Doppler velocity in the absence of transfusion support in children with sickle cell anaemia, elevated transcranial Doppler velocity, and cerebral vasculopathy: the EXTEND trial. *Br J Haematol* 2021; **195**: 612–20.
  42. Wang WC, Zou P, Hwang SN, *et al.* Effects of hydroxyurea on brain function in children with sickle cell anemia. *Pediatr Blood Cancer* 2021; **68**: e29254.
  43. Estepp JH, Cong Z, Agodoa I, *et al.* What drives transcranial Doppler velocity improvement in paediatric sickle cell anaemia: analysis from the Sickle Cell Clinical Research and Intervention Program (SCCRIP) longitudinal cohort study. *Br J Haematol* 2021; **194**: 463–8.
  44. Karkoska K, Quinn CT, Niss O, Pfeiffer A, Dong M, Vinks AA, McGann PT. Hydroyxurea improves cerebral oxygen saturation in children with sickle cell anemia. Am J Hematol. 2021 May 1;96(5):538-544.
  45. Peine BR, Callaghan MU, Callaghan JH, Glaros AK. Prophylactic Hydroxyurea Treatment Is Associated with Improved Cerebral Hemodynamics as a Surrogate Marker of Stroke Risk in Sickle Cell Disease: A Retrospective Comparative Analysis. *J Clin Med* 2022; **11**: 3491.
  46. Kratovil T, Bulas D, Driscoll MC, Speller-Brown B, McCarter R, Minniti CP. Hydroxyurea therapy lowers TCD velocities in children with sickle cell disease. *Pediatr Blood Cancer* 2006; **47**: 894–900.
  47. Al-Jam’a AH, Al-Dabbous IA. Hydroxyurea in sickle cell disease patients from Eastern Saudi Arabia. *Saudi Med J* 2002; **23**: 277–81.
  48. Alkhunaizi AM, Al-Khatti AA, Alkhunaizi MA. Prevalence of Microalbuminuria in Adult Patients with Sickle Cell Disease in Eastern Saudi Arabia. *Int J Nephrol* 2018; **2018**: 5015764.
  49. Ataga KI, Zhou Q, Saraf SL, *et al.* Longitudinal study of glomerular hyperfiltration in adults with sickle cell anemia: a multicenter pooled analysis. *Blood Adv* 2022; **6**: 4461–70.
  50. Aygun B, Wruck LM, Schultz WH, *et al.* Chronic transfusion practices for prevention of primary stroke in children with sickle cell anemia and abnormal TCD velocities. *Am J Hematol* 2012; **87**: 428–30.
  51. Ballas SK, Martinez U, Savage M. Primary stroke in a woman with sickle cell anemia responsive to hydroxyurea therapy. *Hemoglobin* 2014; **38**: 373–5.
  52. Bernaudin F, Verlhac S, Arnaud C, *et al.* Long-term treatment follow-up of children with sickle cell disease monitored with abnormal transcranial Doppler velocities. *Blood* 2016; **127**: 1814–22.
  53. Brousse V, Gandhi S, De Montalembert M, *et al.* Combined blood transfusion and hydroxycarbamide in children with sickle cell anaemia. *Br J Haematol* 2013; **160**: 259–61.
  54. Daak AA, Dampier CD, Fuh B, *et al.* Double-blind, randomized, multicenter phase 2 study of SC411 in children with sickle cell disease (SCOT trial). *Blood Advances* 2018; **2**: 1969–79.
  55. Dedeken L, Chapusette R, Lê PQ, *et al.* Reduction of the Six-Minute Walk Distance in Children with Sickle Cell Disease Is Correlated with Silent Infarct: Results from a Cross-Sectional Evaluation in a Single Center in Belgium. *PLoS ONE* 2014; **9**: e108922.
  56. Derebail VK, Ciccone EJ, Zhou Q, Kilgore RR, Cai J, Ataga KI. Progressive Decline in Estimated GFR in Patients With Sickle Cell Disease: An Observational Cohort Study. *American Journal of Kidney Diseases* 2019; **74**: 47–55.
  57. Galadanci NA, Abdullahi SU, Tabari MA, *et al.* Primary stroke prevention in Nigerian children with sickle cell disease (SPIN): Challenges of conducting a feasibility trial: Sickle Cell Stroke Prevention Trial in Africa. *Pediatr Blood Cancer* 2015; **62**: 395–401.
  58. Galadanci NA, Johnson W, Carson A, Hellemann G, Howard V, Kanter J. Factors associated with left ventricular hypertrophy in children with sickle cell disease: results from the DISPLACE study. *haematol* 2022; **107**: 2466–73.
  59. Gulbis B. Hydroxyurea for sickle cell disease in children and for prevention of cerebrovascular events: the Belgian experience. *Blood* 2005; **105**: 2685–90.
  60. Helton KJ, Adams RJ, Kesler KL, *et al.* Magnetic resonance imaging/angiography and transcranial Doppler velocities in sickle cell anemia: results from the SWiTCH trial. *Blood* 2014; **124**: 891–8.
  61. Lagunju I, Brown BJ, Oyinlade AO, *et al.* Annual stroke incidence in Nigerian children with sickle cell disease and elevated TCD velocities treated with hydroxyurea. *Pediatr Blood Cancer* 2019; **66**: e27252.
  62. Lagunju I, Brown BJ, Sodeinde O. Hydroxyurea lowers transcranial Doppler flow velocities in children with sickle cell anaemia in a Nigerian cohort: Hydroxyurea Lowers Transcranial Doppler Flow Velocities. *Pediatr Blood Cancer* 2015; **62**: 1587–91.
  63. Lebensburger JD, Aban I, Pernell B, *et al.* Hyperfiltration during early childhood precedes albuminuria in pediatric sickle cell nephropathy. *Am J Hematol* 2019; **94**: 417–23.
  64. Olivieri NF, Vichinsky EP. Hydroxyurea in Children with Sickle Cell Disease: Impact on Splenic Function and Compliance with Therapy. *Journal of PediatricHematology/Oncology* 1998; **20**: 26–31.
  65. Rankine-Mullings AE, Little CR, Reid ME, *et al.*EXpanding Treatment for Existing Neurological Disease (EXTEND): An Open-Label Phase II Clinical Trial of Hydroxyurea Treatment in Sickle Cell Anemia. *JMIR Res Protoc* 2016; **5**: e185.
  66. Rigano P, Pecoraro A, Calvaruso G, Steinberg MH, Iannello S, Maggio A. Cerebrovascular events in sickle cell‐beta thalassemia treated with hydroxyurea: A single center prospective survey in adult Italians. *American J Hematol* 2013; **88**. DOI:10.1002/ajh.23531.
  67. Sabarense AP, Silva CM, Muniz MBDSR, Viana MB. Follow-up of children with sickle cell anemia screened with transcranial Doppler and enrolled in a primary prevention program of ischemic stroke. *Hematology, Transfusion and Cell Therapy* 2022; **44**: 478–84.
  68. Sachdev V, Sidenko S, Wu MD, *et al.* Skeletal and myocardial microvascular blood flow in hydroxycarbamide-treated patients with sickle cell disease. *Br J Haematol* 2017; **179**: 648–56.
  69. Saraf SL, Derebail VK, Zhang X, Gladwin MT, Gordeuk VR, Little JA. Manifestations of Reduced Kidney Function Occur at a Higher Estimated Glomerular Filtration Rate in Sickle Cell Anemia. *Blood* 2019; **134**: 2268–2268.
  70. Zimmerman SA, Davis JS, Mortier NA, Ware RE. Elevated Glomerular Filtration Rate (GFR) in Young Patients with Sickle Cell Anemia. *Blood* 2004; **104**: 3745–3745.
  71. Smart LR, Ambrose EE, Balyorugulu G, *et al.* Stroke Prevention with Hydroxyurea Enabled through Research and Education: A Phase 2 Primary Stroke Prevention Trial in Sub-Saharan Africa. *Acta Haematol* 2023; **146**: 95–105.
  72. Stallworth JR, Tripathi A, Jerrell JM. Prevalence, Treatment, and Outcomes of Renal Conditions in Pediatric Sickle Cell Disease: *Southern Medical Journal* 2011; **104**: 752–6.
  73. Voskaridou E, Terpos E, Margeli A, *et al.* Long Term Treatment with Hydroxyurea Does Not Prevent Development of Renal Dysfunction and Osteodystrophy in Patients with Sickle Cell Disease. *Blood* 2004; **104**: 1675–1675.
  74. Yan J-H, Ataga K, Kaul S, *et al.* The Influence of Renal Function on Hydroxyurea Pharmacokinetics in Adults With Sickle Cell Disease. *The Journal of Clinical Pharmacology* 2005; **45**: 434–45.
  75. Yu T, Campbell T, Ciuffetelli I, *et al.* Symptomatic Avascular Necrosis: An Understudied Risk Factor for Acute Care Utilization by Patients with SCD. *Southern Medical Journal* 2016; **109**: 519–24.
  76. Zahr RS, Hankins JS, Kang G, *et al.* Hydroxyurea prevents onset and progression of albuminuria in children with sickle cell anemia. *Am J Hematol* 2019; **94**: E27–9.
